# The “RCT augmentation”: a novel simulation method to add patient heterogeneity into phase III trials

**DOI:** 10.1186/s12874-018-0534-6

**Published:** 2018-07-06

**Authors:** Helene Karcher, Shuai Fu, Jie Meng, Mikkel Zöllner Ankarfeldt, Orestis Efthimiou, Mark Belger, Josep Maria Haro, Lucien Abenhaim, Clementine Nordon, on behalf of the GetReal Consortium Work Package 2

**Affiliations:** 1 Analytica Laser, Audrey House, 16-20 Ely Place, London, EC1N 6SN UK; 2 Analytica Laser, Loerrach, Germany; 3 Novo Nordisk A/S, Soeborg, Denmark; 4 Julius Center for Health Sciences and Primary Care, University Medical Center Utrecht, Utrecht, The Netherlands; 5 Optimed, Clinical Research Centre, Copenhagen University Hospital, Hvidovre, Denmark; 6 Department of Hygiene and Epidemiology, University of Ioannina School of Medicine, Ioannina, Greece; 7 Institute of Social and Preventive Medicine, University of Bern, Bern, Switzerland; 8 Eli Lilly and Company, Lilly Research Centre, Windlesham, UK; 9 Parc Sanitari Sant Joan de Déu, CIBERSAM, Universitat de Barcelona, Sant Boi de Llobregat, Barcelona, Spain; 10 LASER Core, Paris, France; 11 INSERM U1178 CESP Maison Blanche Public Hospital, Paris, France

**Keywords:** Patient heterogeneity, External validity, Real-world, Pragmatic trials, Clinical drug development, Optimal trial design, Effectiveness, Predictive modeling, Modeling and simulation study, Comparative effectiveness

## Abstract

**Background:**

Phase III randomized controlled trials (RCT) typically exclude certain patient subgroups, thereby potentially jeopardizing estimation of a drug’s effects when prescribed to wider populations and under routine care (“effectiveness”). Conversely, enrolling heterogeneous populations in RCTs can increase endpoint variability and compromise detection of a drug’s effect. We developed the “RCT augmentation” method to quantitatively support RCT design in the identification of exclusion criteria to relax to address both of these considerations. In the present manuscript, we describe the method and a case study in schizophrenia.

**Methods:**

We applied typical RCT exclusion criteria in a real-world dataset (cohort) of schizophrenia patients to define the “RCT population” subgroup, and assessed the impact of re-including each of the following patient subgroups: (1) illness duration 1–3 years; (2) suicide attempt; (3) alcohol abuse; (4) substance abuse; and (5) private practice management. Predictive models were built using data from different “augmented RCT populations” (i.e., subgroups where patients with one or two of such characteristics were re-included) to estimate the absolute effectiveness of the two most prevalent antipsychotics against real-world results from the entire cohort. Concurrently, the impact on RCT results of relaxing exclusion criteria was evaluated by calculating the comparative efficacy of those two antipsychotics in virtual RCTs drawing on different “augmented RCT populations”.

**Results:**

Data from the “RCT population”, which was defined with typical exclusion criteria, allowed for a prediction of effectiveness with a bias < 2% and mean squared error (MSE) = 5.8–6.8%. Compared to this typical RCT, RCTs using augmented populations provided improved effectiveness predictions (bias < 2%, MSE = 5.3–6.7%), while returning more variable comparative effects. The impact of augmentation depended on the exclusion criterion relaxed. Furthermore, half of the benefit of relaxing each criterion was gained from re-including the first 10–20% of patients with the corresponding real-world characteristic.

**Conclusions:**

Simulating the inclusion of real-world subpopulations into an RCT before running it allows for quantification of the impact of each re-inclusion upon effect detection (statistical power) and generalizability of trial results, thereby explicating this trade-off and enabling a controlled increase in population heterogeneity in the RCT design.

**Electronic supplementary material:**

The online version of this article (10.1186/s12874-018-0534-6) contains supplementary material, which is available to authorized users.

## Background

Patient populations recruited in phase III randomized-controlled trials (RCT) tend to be more homogeneous compared to those likely to be prescribed the drug under investigation in real-world clinical practice. Such homogeneity is the result of excluding patients with certain characteristics, such as comorbidities, risk factors or co-medications. This practice may compromise the generalizability of results to real-world patient populations [1, 2]. For example, Heng et al. [3] found that more than a third of real-world patients with metastatic renal cell carcinoma are ineligible for clinical trials, showing poorer outcomes than patients enrolled in such clinical trials. In schizophrenia, an even higher proportion of screened patients (73–93%) has been reported as not participating in clinical trials [4–7]. It is often unknown how trial exclusion criteria impact estimations of drug effects, possibly exaggerating or underestimating them [8, 9] and, in general, there is no evidence at time of launch to support treatment guidance for (real-world) patients who were excluded from trials conducted as part of the process of drug approval [10]. Predictive models can be used to extrapolate the drug effect from RCT into effectiveness in the broader real-world population [11]. However, such model predictions are only as good as the underlying data (or assumptions) on the way the new drug effect is modulated by different real-world patient characteristics. In particular, predictions of drug effects on patient subgroups with a characteristic of a categorical nature, such as having a comorbidity or not, can be poor whenever the subgroup has been fully excluded from RCTs, implying that no data exist on the interaction of the drug and the patient subgroup. Pressler and Kaizar [12] have developed a method relying on real world data to estimate the gap between RCT efficacy and real world effectiveness, which they term “RCT generalizability bias”. However, considerations of RCT design, such as constant sample sizes and the necessary trade-off between minimizing this “RCT generalizability bias” and retaining enough RCT statistical power remains unexplored.

While it is essential for all stakeholders - drug manufacturers, payers, clinical practitioners, and patients - that drug development trials provide as much information as possible on real-world effectiveness, fully representative populations are not currently included in trials due to concerns about patient safety, ethics, and the ability to detect a drug effect from a relatively small and controlled sample of patients. A more heterogeneous trial population could indeed add variability to the trial endpoint(s) and/or lead to smaller average treatment differences between arms, that is, to smaller effect sizes, which require larger populations to attain the same statistical power [13–15]. While larger trials are costlier, endpoints with a higher variability may translate in trials that take longer to read out [16]. In addition, recruiting certain types of population may require additional efforts [17]. Ultimately, trialists need to strike a high-stake compromise between representativeness, that is, the RCT’s external validity, and confidence in obtaining the desired effect sizes from a trial enrolling a set number of patients (internal validity).

Considering this, we developed a novel modeling and simulation method, the “RCT augmentation” method, to support the design of RCTs. The method uses real-world data to simulate the impact of including certain patient populations typically excluded from phase III RCT on: (1) the ability to detect the investigational drug efficacy, i.e., the RCT’s statistical power, and (2) the accuracy of predictions of the drug’s effectiveness, i.e., how accurately the data to be collected in the RCT will enable subsequent prediction of the drug effects in routine care.

The objective of our work was to test and validate this method through implementation of a case study in schizophrenia. Namely, we quantified the impact of re-including certain real-world patients typically excluded from Phase III trials in schizophrenia (i.e., by broadening inclusion criteria) upon both the assessment of antipsychotic drug effects and the prediction of their effectiveness.

## Methods

### The “RCT augmentation” method

#### General methodology

The “RCT augmentation” method aims at supporting RCT design by quantifying the trade-off between partially relaxing certain exclusion criteria to collect the most informative data to predict treatment’s real-world effects, while preserving the trial’s statistical power. The method requires real-world data (e.g., observational cohort, disease registry, electronic healthcare records, claims databases) on patient characteristics and treatment outcome in the indication of interest. Several scenarios (virtual RCTs, augmented or not) are tested through simulations using these real-world data to quantify the impact of using different population eligibility criteria for the planned RCT. All virtual RCTs, augmented or not, have an identical target sample size. Operationally, augmented RCTs correspond to trials where the protocol allows for inclusion of the subset of patients that meets the relaxed criterion or criteria, while keeping all other, remaining exclusion criteria. The main results of the augmented RCTs are obtained as for regular RCTs, i.e., by calculating the primary (and secondary) endpoints on all enrolled patients.

#### Terminology

The subset of patients susceptible to take the drug of interest, once it becomes available on the market, is termed hereafter “real-world population”. Applying typical RCT exclusion criteria to this population returns the “RCT population” subset. Replacing a part of patients from the RCT population by patients meeting relaxed exclusion criteria creates “augmented RCT populations”. The target estimand of effectiveness prediction models is the drug effectiveness in the real world population, while the (augmented) RCT estimand is the drug efficacy in the (augmented) RCT population.

#### Step-by-step implementation

After selection of an appropriate real-world data source, the “RCT population” is first generated by applying exclusion criteria traditionally used in RCTs for the indication of interest to the “real-world population”. The representativeness of a typical RCT (i.e., using traditional inclusion/exclusion criteria) can be first judged at this stage by comparing the distribution of patient characteristics and outcomes between the “RCT population” and the “real-world population”.

Second, the exclusion criteria that could potentially be relaxed, at least partially, are identified and listed. By contrast, exclusion criteria that are necessary for safety or ethical reasons are not considered.

Third, virtual RCT scenarios are tested, in which various patients subgroups are “re-included” in a controlled proportion by relaxing exclusion criteria one by one or two by two. These “re-inclusions” are performed at constant RCT size, that is, by replacement, to keep subsets of identical patient size and keeping other exclusion criteria fixed. This broadens the RCT population and creates a range of “augmented RCT population” subsets. The augmentation is done by replacement to keep subsets of identical patient size and facilitate their comparison. For each RCT scenario, predictive models of the outcome of interest (endpoint for the trial or a proxy for it) are built successively on data from the “RCT population” or the different “augmented RCT” populations and predictions compared to the outcome in the full real-world population. The method is agnostic to the type of model chosen for effectiveness prediction, which can range from simple multivariate regression to more advanced models. This comparison of predicted versus observed outcomes enables a thorough appraisal of the representativeness of each (augmented) RCT population and of its expected usefulness in accurately predicting the new drug’s effectiveness (external validity).

At the same time, virtual RCTs with the envisaged sample size are built using patients from either “RCT population” or the different “augmented RCT populations”. They enable to calculate the distribution of possible trial results for each of these source populations (efficacy or comparative efficacy), which in turns allows for calculation of the trial’s statistical power.

Ultimately, the method provides trialists with both the increase in representativeness, if any, and the change in statistical power (equals the ability to detect a given effect size) for each augmentation in a particular type of real-world patients. In other words, the method quantifies the impact of amending the trial protocol to relax one or two exclusion criteria, possibly partially only, on representativeness and trial statistical power. The trade-off between these two goals of Phase III RCTs can then be chosen in a controlled and informed manner.

### Case study: Population selection for phase 3 RCTs in schizophrenia

#### Data source

The present case study used observational patient-level data from the Schizophrenia Outpatients Health Outcomes (SOHO) study, [18, 19] a prospective cohort study addressing symptom improvement, associated with the introduction or switch of antipsychotic drugs in schizophrenia outpatients, that is, patients managed in ambulatory or community settings. The SOHO study followed 10,218 adults with schizophrenia from across ten European countries over a 3-year period. All the participants initiated or switched antipsychotic drug therapy at baseline. Use of concomitant medications was allowed during the study, but not reported by physicians. Data on demographics, clinical status, functioning, use of other psychotropic drugs and adherence to the previously-used drug were collected during routine visits at baseline, 3, 6, and 12 months. For the present case study, due to confidentiality agreements, the names of antipsychotic drugs were anonymized by assigning each a letter (drug D1, drug D2, drug D3, etc.). In addition, in an effort to avoid drug identification through given doses, these were converted into the equivalent defined daily dose (DDDeq) by dividing the prescribed dose by the World Health Organization (WHO)’s defined daily dose [20].

The SOHO cohort was assumed to be representative of schizophrenia patients as managed in routine care, and change in symptoms severity was assumed to reflect the real-world effect of the newly-initiated antipsychotic drug for each patient. In our study, baseline and 3-month data were used. Any change in the Clinical Global Impression – Severity score (CGI-S, using a 7-point score) at 3 months after the last adjustment of antipsychotic treatment (new treatment or switch at baseline) was registered as a change in symptoms (ΔCGI-S, change in symptoms hereafter). This change in symptoms was assumed to represent the real-world effect of the newly-initiated antipsychotic drug. The earliest available time point (3 months) of the outcome was selected to facilitate the comparison of results with previously-published RCTs, the majority of which do not report data beyond this period [21]. In addition, only patients starting on one of the two most-frequently used antipsychotic drugs (D1 or D2) in SOHO at baseline (*N* = 5591 for D1 and 2188 for D2) were selected to generate two treatment groups. Patients were grouped by type of newly-initiated drug (D1 or D2). The methods employed below were identical for drug D1 and D2.

#### Exclusion criteria of interest and construction of the “RCT population”

First, exclusion criteria traditionally used in Phase III RCTs in schizophrenia were identified using a meta-analysis of more than 200 antipsychotic clinical trials in schizophrenia [22] and a literature research on pivotal Phase III RCTs across ClinicalTrials.gov, EU Clinical Trials Register, WHO International Clinical Trials Registry Platform (WHO ITCRP), and PubMed websites, which returned 30 trials on eight different second-generation antipsychotic drugs marketed since 1991 [23]. The strictest inclusion thresholds were used to define the criteria most commonly used. The identified exclusion criteria were: (1) Illness duration < 3 years; (2) History of suicide attempt (a proxy for risk of suicide attempt and for depression); (3) History of alcohol abuse; (4) History of drug abuse; (5) Non-compliance according to the physician and (6) Patients followed by a psychiatrist who practices only in a private setting (“private practice patients” hereafter) The last exclusion criterion listed above, while not an explicit exclusion criterion, reflected the fact that physicians enrolling patients for RCTs usually practice at least partially in a public setting, such as a university hospital. In the end, the “RCT population” for each drug group resulted from applying the six exclusion criteria above to the SOHO population. No other exclusion criteria were applied; in particular, criteria related to safety or relying for their definition on variables not present in the SOHO database were not used.

**Fig. 1:**
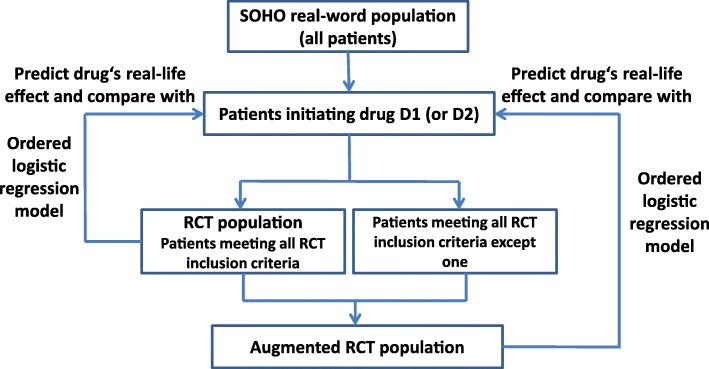
Definition of the different populations used for analysis and predictive modeling. The process was repeated for the most-frequently initiated drugs in SOHO, D1 and D2

Second, we applied these 6 exclusion criteria to the SOHO population to exclude patients for each drug group (starting either D1 or D2) to define a “RCT population” for D1 and D2 (Fig. 1). Change in symptoms in the RCT population carved out from SOHO fell within a similar range to those from six RCT studies identified in the literature (see details in Additional file 1: section 3 “Comparison of CGI-S from the “RCT population” subset with values obtained for RCTs published in the literature”).

#### Analyses, modeling and simulations

Several virtual Phase III RCTs in schizophrenia were simulated by using different exclusion criteria to assess modeling predictions obtained against real-world outcomes from SOHO (Table 1). The “RCT population augmentation” aimed at selectively increasing the population’s heterogeneity, thereby creating a series of “augmented RCT populations”. Namely, a set number of patients from the “RCT population” were randomly replaced by an equal number of originally excluded patients (Fig. 1); all re-included patients fulfilled the six above-listed exclusion criteria except one, which was replaced by one of the following relaxed criteria:

- Patients with illness duration between 1 and 3 years
- Patients with one past suicide attempt
- Private practice patients
- Patients with history of alcohol abuse
- Patients with history of drug abuse

**Table 1:** Relative sizes of patient populations and outcomes across population types

Population type	% increase in RCT-eligible patient pool (95% CI^c^)	Outcome Average ΔCGI-S (95% CI^d^)
Patients initiating drug D1		
“Real-world population” (SOHO cohort)	**–**	−0.88 (− 0.90, − 0.85)
“RCT population” (22.6% of SOHO cohort patients)	0	− 0.78 (− 0.83, − 0.72)
*Augmented RCT populations:*		
Patients with illness duration between 1 and 3 years^a^	17.9% (15.5, 20.5%)	−1.04 (− 1.18, − 0.89)
Patients with one past suicide attempt^a^	18.1% (15.7, 20.8%)	− 0.94 (− 1.08, − 0.81)
Private practice patients^a^	14.7% (12.6, 17.1%)	−1.03 (− 1.15, − 0.90)
Patients with history of alcohol abuse^a^	6.3% (5.0, 7.8%)	−0.60 (− 0.79, − 0.42)
Patients with history of drug abuse^a^	5.6% (4.4, 7.0%)	−0.78 (− 0.99, − 0.57)
Patients with illness duration between 1 and 3 years and/or one past suicide attempt^b^	37.7% (34.0, 41.9%)	−1.00 (− 1.10, − 0.90)
Patients with illness duration between 1 and 3 years and/or private practice patients^b^	35.1% (31.4, 39.0%)	−1.05 (− 1.15, − 0.96)
Patients with illness duration between 1 and 3 years and/or history of alcohol abuse^b^	25.0% (22.1, 28.3%)	− 0.93 (− 1.05, − 0.81)
Patients initiating drug D2		
“Real-world population” (SOHO cohort)	**–**	−0.71 (− 0.75, − 0.67)
“RCT population” (25.7% of SOHO cohort patients)	0	−0.64 (− 0.72, − 0.57)
*Augmented RCT populations:*		
Patients with illness duration of 1–3 years^a^	11.7% (8.9, 15.1%)	−0.77 (− 1.03, − 0.51)
Patients with one past suicide attempt^a^	15.8% (12.6, 19.8%)	−0.57 (− 0.71, − 0.42)
Private practice patients^a^	14.9% (11.7, 18.7%)	−0.74 (− 0.93, − 0.55)
Patients with history of alcohol abuse^a^	8.7% (6.4, 11.6%)	−0.63 (− 0.97, − 0.30)
Patients with history of drug abuse^a^	4.3% (2.8, 6.5%)	−0.50 (− 0.83, − 0.17)
Patients with illness duration between 1 and 3 years and/or one past suicide attempt^b^	29.4% (24.5, 35.0%)	−0.62 (− 0.76, − 0.49)
Patients with illness duration between 1 and 3 years and/or private practice patients^b^	29.6% (24.7, 35.2%)	−0.79 (− 0.94, − 0.64)
Patients with illness duration between 1 and 3 years and/or history of alcohol abuse^b^	20.5% (16.7, 25.1%)	−0.73 (− 0.93, − 0.52)

^a^plus meeting the remaining 5 RCT eligibility criteria. ^b^plus meeting the remaining 4 RCT eligibility criteria; ^c^The Clopper-Pearson interval was used to calculate the 95% confidence interval; *CI* confidence interval; ^d^The confidence interval (CI) was calculated under the assumption that ΔCGI-S had a normal distribution

As a result, a series of five types of augmented RCT populations were obtained. Please note that the exclusion criteria above were relaxed in a “controlled” manner so that re-inclusion of patients meeting the criteria was, a priori, safe and feasible. For example, including all patients who have attempted suicide could be difficult to manage in a trial if allowing the inclusion of patients with 10+ suicide attempts. As a result of controlling exclusion criteria, we selectively opened the trial to patients with a single past suicide attempt only. Similarly, enrolling patients recently diagnosed with schizophrenia into a clinical trial of a new drug is unlikely, given the large number of effective first-line therapies available on the market; therefore, only patients who have experienced the condition for longer than 1 year were considered for re-inclusion. For similar reasons, patients were not enrolled if they were non-compliant.

Besides the five types of augmented RCT populations selected as described above, three additional augmented RCT populations were created by relaxing two selected criteria at the same time:

- Patients with illness duration between 1 and 3 years and/or one past suicide attempt
- Patients with illness duration between 1 and 3 years and/or private practice patients
- Patients with illness duration between 1 and 3 years and/or history of alcohol abuse

Again, simultaneously relaxing two criteria as above was acceptable as it did not compromise safety a priori (which could be the case for example when combining past suicide attempts and drug abuse), while seeming operationally feasible a priori also.

#### Predictive model building and simulation study

Real-world change in symptoms at 3 months was predicted using a model of the symptom score at 3 months, CGI-S, and subtracting the baseline score. The outcome (CGI-S) is an ordered categorical variable, measured on a 7-point scale. It was therefore modeled using a latent variable, to which an ordinal logistic regression model was applied [24]. The regression variables used to fit the model included all of 24 variables available in SOHO: patient characteristics, baseline symptoms, and drug dosages (see subsection 1.3. Predictive model in Additional file 1 for further details). This statistical model was trained using the newly-built data from the “RCT population” or “augmented RCT populations”, that is, the model estimated for each population a different set of cut-offs for the latent variable. The same model structure, including all regression variables, was used across all the analyses; models differed only in terms of the data used to train them (i.e., the RCT or augmented RCT populations used as training sets). This way, differences in model predictions were only attributable to the data used to train it (i.e., data originating from the RCT or the augmented RCT populations) and not its structure. The model was then used to simulate change in symptoms in the SOHO population using only baseline values from SOHO patients. We compared predictions across changes in symptoms observed for the whole real-world population, using the SOHO population as test set for external validation. Specifically, bias and mean squared error (MSE) of predictions were calculated to gauge how re-including patients with specific characteristics could improve real-world outcome predictions made using RCT data only (see equations in Additional file 1: Definition of bias and MSE for model predictions). Bias and MSE tend towards zero as prediction performance improves, that meaning the distribution of predicted changes in symptoms in the real-world population becomes closer to its actual distribution as observed in the full SOHO cohort. For each selected exclusion criterion, we tested a series of augmentation percentages. The number of replaced patients was increased gradually until the percentage of patients with that specific characteristic reached “natural augmentation”. “Natural augmentation” was defined as the proportion of real-world patients in a population including all patients meeting the relaxed criterion and the five remaining exclusion criteria, without stratified randomization at patient enrollment.

#### Comparative efficacy in virtual, augmented RCTs

**Fig. 2:**
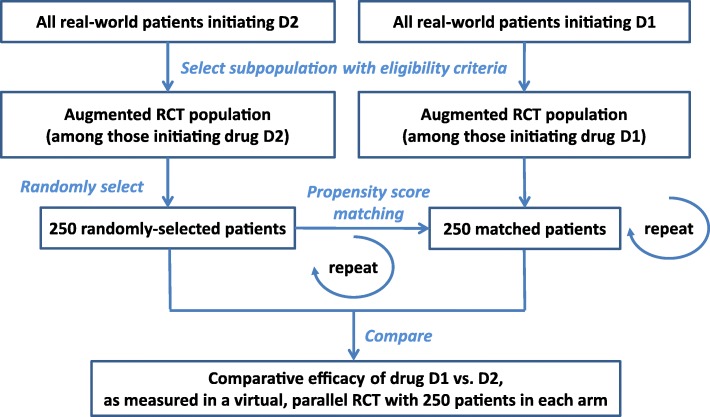
Calculation of comparative efficacy of drug D1 vs. D2 obtained from virtual Phase III RCTs. This calculation is repeated for each type of RCT population augmentation (= each relaxed criterion)

In addition, to assess how population augmentation impacted trial results, the comparative efficacy obtained from virtual Phase III RCTs with two parallel arms in which 500 patients initiated either of the two most prevalent drugs (D1 and D2) was computed (Fig. 2). A robust estimate of comparative efficacy is indeed what trialists rely on to calculate the trial’s statistical power. First, 250 patients from the “augmented RCT population” started on drug D2 (as defined previously) were randomly selected. Next, another 250 patients who started on drug D1 were selected from the equivalent “augmented RCT population” by propensity score matching (see subsection 1.2 for methodological details in Additional file 1). We compared the average change in symptoms between these 250-paired patients, which was termed the comparative efficacy of D1 vs. D2 for the virtual trial. To reduce the effects of random sampling errors, sampling and matching were repeated to evaluate 1000 virtual Phase III RCT samples (bootstrapping) for each relaxed criterion, i.e., for each RCT population augmentation. Comparative efficacy was reported as the distribution of these 1000 sampling results. In addition, the same repeated process of selection and matching was also applied to the entire SOHO population to obtain comparative effectiveness of drug D1 vs. D2 (Fig. 2). Further details on calculation of comparative efficacy can be found in Additional file 1: Section 1.2).

We used R (version 3.1.2) for all analyses [25].

## Results

### Differences between real-world and RCT populations in schizophrenia

Applying all six exclusion criteria typical of Phase III trials to the SOHO real-world population left only 22.6 and 25.5% of patients initiating drug D1 and drug D2, respectively, eligible for inclusion in the “RCT population”. For both drugs, the magnitude of symptoms improvement was greater in the SOHO population compared to the “RCT population” (Drug D1: − 0.88 (− 0.90, − 0.85) vs. -0.78 (− 0.83, − 0.72); Drug D2: − 0.71 (− 0.75, − 0.67) vs. -0.64 (− 0.72, − 0.57), see Table 1).

The size of the subpopulations to be re-included in the “RCT population” differed depending on the exclusion criterion relaxed (Table 2 and Table 3). Namely, opening the trial to patients with shorter illness duration (1–3 years), one past suicide attempt or to private practice patients enabled to tap into a wider patient pool than opening the trial to patients with alcohol or drug abuse, e.g., among patients taking D1, the patient pool was increased by 188 (shorter illness duration), 190 (past suicide attempt) and 159 (private practice) patients compared to 73 (alcohol) and 65 (drug abuse) patients.

**Table 2:** Comparison of the impact of relaxing different eligibility criteria in patients taking drug D1

Re-included subpopulations		Natural augmentation (number of patients re-included when opening the trial to the specific “real-world population” subgroup)	Prediction bias with natural augmentation	Mean squared error (MSE) of prediction with natural augmentation
Relaxed eligibility criteria	Illness duration between 1 and 3 years	188	0.033	0.818
	1 past suicide attempt	190	0.057	0.820
	Private practice	159	0.041	0.830
	Alcohol abuse	73	0.054	0.836
	Drug abuse	65	0.053	0.833
	Illness duration between 1 and 3 years + 1 past suicide attempt	339	0.024	0.803
	Illness duration between 1 and 3 years + private practice	321	0.024	0.814
	Illness duration between 1 and 3 years + alcohol abuse	248	0.037	0.812
RCT population		not applicable	0.054	0.852
SOHO “real-world population”		not applicable	0.000	0.000

Results for the “RCT population” and SOHO “real-world populations” are displayed as benchmark

**Table 3:** Comparison of the impact of relaxing different eligibility criteria in patients taking drug D2

Re-included subpopulations		Natural augmentation (when opening the trial to the specific “real-world population” subgroup)	Prediction bias with natural augmentation	Mean squared error (MSE) of prediction with natural augmentation
Relaxed eligibility criteria	Illness duration between 1 and 3 years	56	0.004	0.733
	1 past suicide attempt	73	0.013	0.734
	Private practice	69	0.003	0.737
	Alcohol abuse	42	−0.008	0.749
	Drug abuse	22	−0.004	0.756
	Illness duration between 1 and 3 years + 1 past suicide attempt	121	0.033	0.716
	Illness duration between 1 and 3 years + private practice	121	0.009	0.719
	Illness duration between 1 and 3 years + alcohol abuse	90	0.011	0.728
RCT population		not applicable	−0.016	0.777
SOHO “real-world population”		not applicable	0.000	0.000

Results for the “RCT population” and SOHO “real-world populations” are displayed as benchmark

Different subpopulations considered for re-inclusion exhibited different outcomes (as measured by the ΔCGI-S). As expected, certain subpopulations tended to benefit more from treatment: patients with illness duration between 1 and 3 years or private practice patients; their re-inclusion into the trial was tapping into a population having on average a more favorable ΔCGI-S than the average score obtained in the original “RCT population”.

### Predictions of real-world effects from RCT population data

**Fig. 3:**
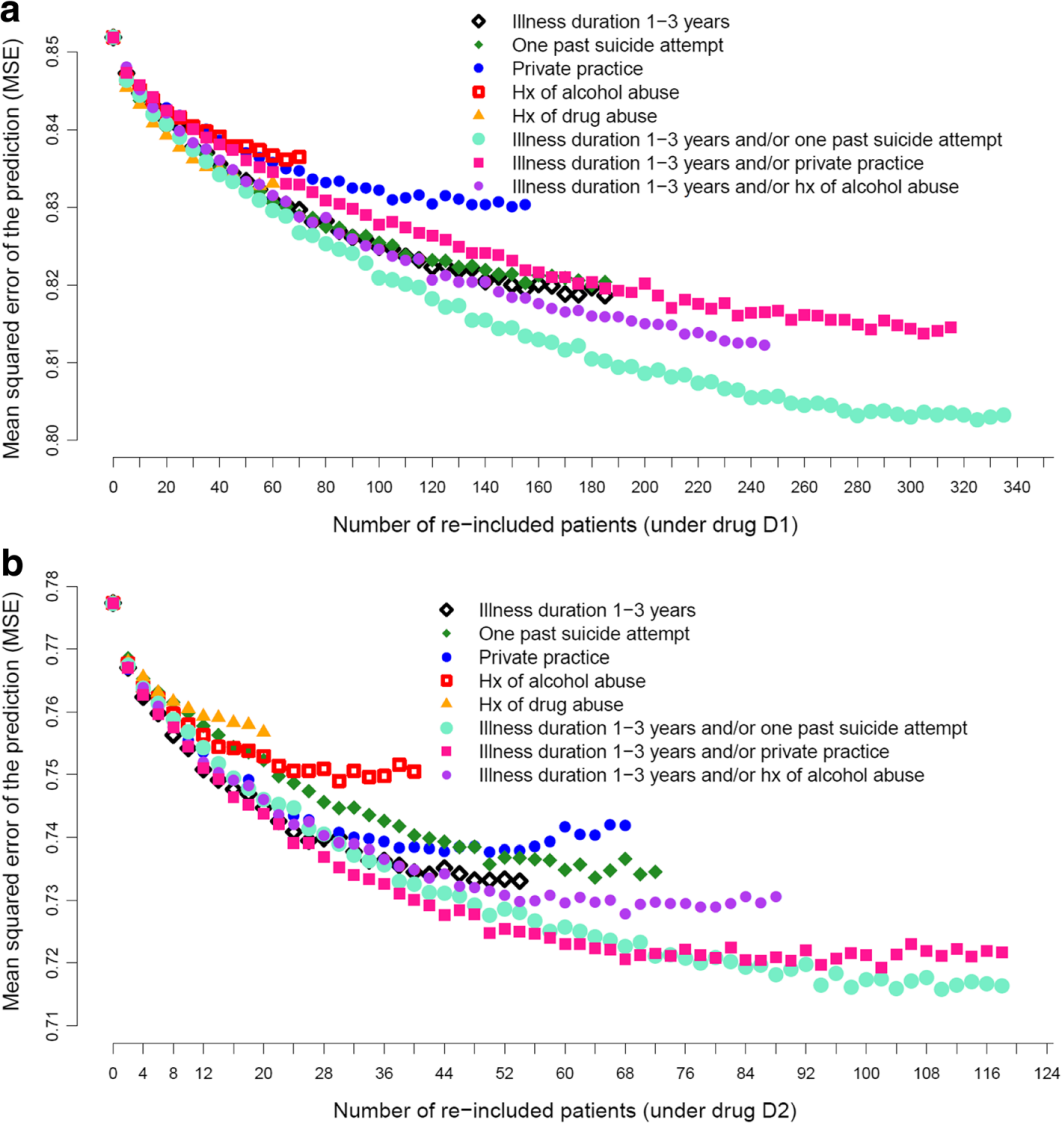
Mean squared error of the prediction from model fitted to data from augmented RCT populations of patients initiating drug D1 (**a**) or drug D2 (**b**). The augmentation was performed by re-including, through random replacement within the RCT population, an increasing number of patients (x-axis) from eight different real-world subpopulations (colored markers) until the natural percentage of the patients with that specific characteristic was reached (right end of each curve). Each point represents an average of 500 random samplings of re-included patients

Using data from the “RCT population” alone yielded prediction models for real-world effects of D1 and D2 associated with a bias of 0.054 and − 0.016 and an MSE of 0.85 and 0.78 for participants under drug D1 and D2, respectively (values for x = 0 in Fig. 3). That is, the absolute value of the bias represented less than 2 % of the average real-world CGI-S at 3 months (0.054/3.54 = 1.5% under drug D1 and 0.016/3.67 = 0.4% under drug D2), while the MSE of the prediction represented about 6–7% of the average of squared real-world CGI-S values at 3 months (0.85/12.53 = 6.8% under drug D1 and 0.78/13.47 = 5.8% under drug D2).

The augmentation of the “RCT populations” initiating drug D1 or D2 by relaxing any of the pre-selected eight exclusion criteria led to improvement of the prediction of real-world effects in terms of MSE (6.4–6.7% under drug D1, 5.3–5.6% under drug D2). The bias remained under 2%. A more heterogeneous RCT population yielded, as expected, better predictions of real-world effects (see Fig. 3a and Table 2 for RCT populations initiating drug D1, Fig. 3b and Table 3 for RCT populations initiating drug D2).

Upon relaxing any of the eight exclusion criteria, we first observed a decrease in MSE as a result of augmenting the “RCT population” through the inclusion of patients with real-world characteristics (Fig. 3). As more real-world patients were re-included, the decrease in MSE became gradually weaker. In other words, benefit in terms of effectiveness prediction is mostly gained from re-including the first few real-world patients from each subpopulation.

As shown in Fig. 3, Tables 2 and 3, re-inclusion of different real-world subpopulations contributed differently to improving prediction. Re-including patients with illness duration between 1 and 3 years and/or with one past suicide attempt (with natural augmentation) returned the most accurate prediction (lowest MSE, corresponding to an improvement in MSE of 0.803 (5.8%) and 0.716 (4.9%) for drug D1 and D2, respectively, see Tables 2 and 3). Among eligibility criteria relaxed as single elements, patients with illness duration between 1 and 3 years yielded the most accurate prediction (see Tables 2 and 3). Note that using these eligibility criteria also enabled to re-include patients from the larger subpopulation pools, that is, from the “augmented RCT populations” of larger sizes, and/or the subpopulation pools with the larger change in symptoms (see Table 1).

### Comparative efficacy in virtual RCTs using augmented populations

**Fig. 4:**
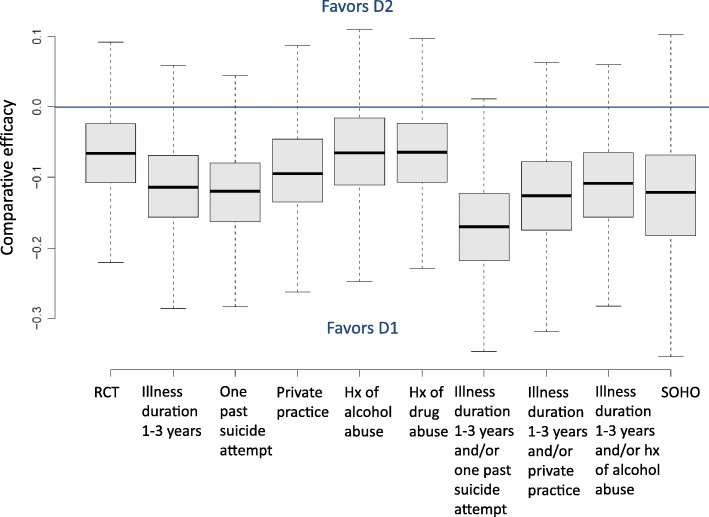
Comparative efficacy of virtual RCTs comparing drug D1 and drug D2, in two parallel study arms with 250 patients each. Source populations are displayed on the x-axis: RCT or augmented RCTs as a result of relaxing any of the eight eligibility criteria. Comparative effectiveness is reported for the real-world population in the full SOHO cohort. Each box plot represents the distribution of comparative efficacy values obtained for the 1000 sampling replicates (bootstrapping)

We simulated virtual RCTs comparing drug D1 and D2 to estimate how the evaluation of comparative efficacy would be impacted when augmenting trial populations, a strategy which could add variability in the outcome and change its estimation in RCTs. Comparative effectiveness of drug D1 vs. D2, which we calculated by propensity score matching on the real-world data, was found to favor drug D1 over D2; the change in symptoms under D1 was − 0.12 ± 0.08 points larger for D1 than D2 (not clinically-significant). Comparative efficacy as measured in virtual RCTs with six standard exclusion criteria was less favorable to D1, with a change in symptoms for D1 vs. D2 of − 0.07 ± 0.06 points (Fig. 4 and Table 4).

**Table 4:** Comparative efficacy of virtual RCTs comparing drug D1 and drug D2

Re-included subpopulations		Average comparative efficacy of D1 vs. D2 (ΔCGI-S for D1 minus ΔCGI-S for D2)	Standard deviation of comparative efficacy D1 vs. D2 (ΔCGI-S for D1 minus ΔCGI-S for D2)
Relaxed eligibility criteria	Illness duration between 1 and 3 years	− 0.113	0.067
	1 past suicide attempt	−0.122	0.064
	Private practice	−0.092	0.064
	Alcohol abuse	−0.064	0.066
	Drug abuse	−0.066	0.064
	Illness duration between 1 and 3 years + 1 past suicide attempt	− 0.171	0.072
	Illness duration between 1 and 3 years + private practice	− 0.127	0.071
	Illness duration between 1 and 3 years + alcohol abuse	− 0.110	0.067
RCT population		−0.066	0.062
SOHO “real-world population”		−0.124	0.084

The results were generated in two parallel study arms with 250 patients each

Average comparative efficacy increased when re-including specific real-world patients (i.e., it was more favorable to D1 vs. D2, thereby closer to the real-world comparative effectiveness) across all of the augmented RCT population tested, except for augmentation using patients with a history of alcohol or drug abuse where it was almost unchanged (Table 4). Virtual trials that returned a comparative efficacy closest to comparative effectiveness were trials with populations augmented in patients with one past suicide attempt (comparative efficacy of − 0.122 ± 0.064 points) and trials augmented in patients with illness duration between 1 and 3 years and/or private practice patients (comparative efficacy of − 0.127 ± 0.071 points). The standard deviation of comparative efficacy varied from 0.06 in the RCT population to 0.08 in SOHO, with intermediate values for augmented trials.

## Discussion

We report the development and testing of a novel modeling and simulation method, the “RCT augmentation” method, enabling trialists to select inclusion/exclusion criteria based on an estimation of the impact that this population selection will have on (1) the trial’s results and (2) the trial’s ability to inform on the effectiveness of the investigational drug (i.e., provide data that best predicts the drug’s effectiveness). The trial results (i.e., the calculated distribution of comparative efficacy from virtual RCTs) can be directly used to calculate the trial’s statistical power, and thereby its ability to detect effect sizes for the studied endpoint.

### Key findings

The present case study provides preliminary information on the scope and application of the method. First, our results suggest that relaxing certain eligibility criteria (i.e., including a part of the real-world patients in the “RCT population”) provide RCT results of comparative efficacy that are closer to comparative effectiveness, thereby narrowing the “efficacy-to-effectiveness gap”. In addition, data obtained from these augmented RCTs can be used to improve effectiveness predictions using modeling (lowering the MSE, bias remaining < 2%). In our case study, the comparative efficacy improved with population augmentation because comparative treatment effect on patients excluded – as a result of applying certain criteria – was on average better than that measured for the “RCT population”. In cases where comparative effectiveness (or effectiveness) would be on average lower than comparative efficacy (or efficacy), a decrease in trial statistical power is expected with population augmentation, along with the gain in precision for real-world effects. In these cases as well, our method enables to quantify the terms of the compromise to be made between trial’s results (comparative efficacy, including its variability) and effectiveness prediction/representativeness. Our results reiterate findings from studies conducted in other indications that exploring outcomes in real-world patient subgroups can better inform predictions of real-world effects for the population as a whole [26]. The two factors driving improvement in predicting real-world effects (i.e., lowering MSE while keeping bias < 2%) were found to be 1) a larger size of the subpopulation of real-world patients originally excluded and 2) better outcomes in these subpopulations (i.e., outcomes closer to those obtained for the full real-world population). Similarly, we found that RCTs returned comparative efficacy results that are more variable if augmented in real-world populations, as previously demonstrated in the general case [13–15].

Second, our results show that applying six of the eligibility criteria most typically used for RCTs in schizophrenia (namely, illness duration < 3 years, history of suicide attempt, of alcohol or drug abuse, non-compliant patients according to the physician, private practice patients) to the SOHO population, considered to represent real-world schizophrenia patients, resulted in the exclusion of more than three quarters of patients. While in accordance with the documented need to conduct effectiveness studies for antipsychotic drugs, [27] the high percentage of patients currently excluded from drug development trials in schizophrenia is worth noting. In addition, the sample size of excluded patients presented here needs to be interpreted in the light of knowledge that RCTs in schizophrenia may employ even more exclusion criteria than those applied in our study. In our study, it was not possible to apply all exclusion criteria commonly used in RCTs due to the corresponding characteristics not being reported in SOHO. Another set of eligibility criteria not applied to define the RCT population from our study is the one pertaining to the risk factors associated with the safety profile intrinsic to the investigational drug. While particular to each individual drug (and not to schizophrenia), it further restricts the selection of trial populations in drug development.

### Practical implications for RCT design

Quantifying the effectiveness prediction accuracy and expected RCT results has practical implications on RCT design. First, the impact of re-including certain patients upon the prediction accuracy of real-world effects, as well as on comparative effects measured in the RCT, varied according to the exclusion criterion relaxed. As an example, opening the trial to patients with one past suicide attempt enables a better prediction of effectiveness, while also providing RCT results (comparative efficacy) that are less variable and closer to the real-world effects, than opening the trial to patients with history of alcohol abuse. In addition, opening the trial to real-world patients with one prior suicide attempt or shorter illness duration enables to tap into a wider patient pool than opening the trial to patients with history of alcohol (or drug) abuse. Assuming all patients were equally easy/difficult to recruit, the former re-inclusion process is likely to enable faster recruitment, which may be a way of preventing long accrual times, in particular in those of disease areas where many clinical trials are competing to recruit the same patients. The interplay of treatment effects and recruitment speed has been previously formalized and tested under a range of assumptions [28]. Once treatment effects are estimated on real-world data, the method by Rudser et al. could be used to extend ours to include quantification of recruitment speed improvement as a factor in the selection of RCT populations. Second, for each relaxed criterion, adding only a few patients improves predictions right at the beginning of the augmentation process (quickly achieving a 50% of the optimal improvement in MSE by adding merely some 10–20% of patients needed for natural augmentation, see Fig. 3), a trend that tends to slow down as the “natural” augmentation percentage is reached. This suggests that, for exclusion criteria that are difficult to relax entirely, it might be worth designing trials that incorporate only a small proportion of these patients. This can be done, for example, by stratifying patients at enrollment so that patients meeting specific exclusion criteria can be re-included in smaller proportions.

Finally, it is interesting to note that the predictions of real-world effects using the model incorporating data from the “RCT population” alone returned sound results in terms of bias (< 2%) and MSE (5–6%) for both drugs D1 and D2, thereby showing that a good prediction can be achieved with RCT patients alone. This should motivate more researchers and drug developers to undertake this type of modeling, still only rarely implemented despite being advocated by many authors [11].

### Applicability of the “RCT augmentation” method

While tested only on second generation antipsychotic drugs in schizophrenia, the method is nonetheless powerful in that it is applicable to other indications and drug classes, with few requirements. Namely, requirements to implement our method to guide the selection of the most suitable population for an upcoming drug development trial include: (i) identifying the exclusion criteria that could potentially be relaxed, at least partially, such as criteria that may not absolutely need to be applied for acute safety issues or ethical reasons; and (ii) locating a real-world data source including suitable patients that captures (a) variables that define the eligibility criteria to be tested, (b) the trial outcomes considered as endpoints, and (c) treatments, where possible, of the same or a similar class as the drug to be investigated in the new trial. In addition, while not demonstrated herewith, this same modeling and simulation approach is applicable to testing how different thresholds used to define the exclusion criterion impact both trial results and effectiveness predictions/representativeness.

Of note, our method is applicable not only to optimize the choice of population at the trial level, as illustrated above, but also at the clinical development program level. For example, consider a situation where the population destined to prove efficacy in a Phase 3 trial and its size are already set. The question is then how to choose the population of patients to enroll in the rest of the program (in the same Phase 3 trial(s), in a subsequent Phase 3b trial). I.e. which type of real-world patients to include in priority to best train the predictive model and estimate real-world effects? and how many of them are needed? The “RCT augmentation” method enables to quantify the improvement upon estimation of real-world effects of including different population subgroups in the clinical program. Finally, we have used an ordinal logistic regression model with 24 patient covariates to illustrate our “RCT augmentation” method. We did not consider other unavailable covariates, such as type of prior medication, nor any interactions between the covariates or between the treatment and the covariates. Alternative, potentially more advanced modelling approaches may increase the power of the prediction model. Such approaches can be readily used within our framework.

### Study limitations

Appropriate real-world data was available to us with schizophrenia patients on second-generation antipsychotics. However, it may be more difficult to find appropriate real-world data to support design of a RCT on a treatment of a completely new class or never-studied mechanism of action; in this case, the assumption of similar patient characteristics-to- treatment effects interactions between drugs used in the real-word data source and the investigational drug to be tested in the RCT would be stronger. Also, as a limitation of using real-world data, estimation of comparative efficacy in virtual RCTs may be biased by unmeasured confounders despite our use of propensity score matching with 24 covariates [29].

It is also important to note that the improvement in prediction accuracy (i.e., decrease in MSE) by augmenting the trial populations was relatively small across all instances studied here. Three reasons can be invoked: 1) low resolution of ΔCGI-S (this outcome provides less information on patients’ symptoms than other widely-used scales in schizophrenia RCTs such as the Brief Psychiatric Rating Scale or the Positive And Negative Syndrome Scale); 2) volunteers in RCTs may differ from those from the “RCT population” defined here (selection bias); and 3) it is possible that re-inclusion criteria are not necessarily the criteria contributing the most to increase generalizability of results from RCT.

Finally, outcomes can differ in RCT and real-world settings not only due to differing population characteristics, as investigated in our study, but also due to different drug usage patterns. For example, RCTs typically deploy closer monitoring, such as several exams and physician visits within the first 3 months (e.g., in recent trials [30–32]), which may entail higher adherence and/or better care overall, and more research-oriented physicians, who may have different prescribing habits to their peers. This additional layer of difference between the two settings would need to be incorporated when estimating real-world effectiveness from conventional RCTs. Our method, however, enabled us to isolate how differences in populations between RCT and real-world practice impact effectiveness evaluation.

## Conclusion

The impact of augmenting the population of schizophrenia RCTs in real-world patients by selectively relaxing a range of exclusion criteria was investigated through modeling and simulations using observational data from a large cohort of schizophrenia patients assumed to represent routine care practice in schizophrenia. By quantifying the impact of each augmentation, our simulations provide a guide to measuring the impact upon prediction of effectiveness and trial statistical power from opening trials to certain types of real-world patients in a controlled manner.

## Additional file

Additional file 1: 1. Details on methods, 2. details on results, 3. comparison of the study results and the data from literature, 4. codes used for the analysis, modeling and simulation, and 5. references used in the Additional file 1. (DOCX 2465 kb)

## Abbreviations

ΔCGI-S: Change in CGI-S at 3 months after the last adjustment made to the antipsychotic treatment (new treatment or switch, at baseline)
CGI-S: Clinical Global Impression – Severity score
CI: Confidence interval
DDDeq: Equivalent defined daily dose
EU: European Union
Hx: History
MSE: Mean squared error
RCT: Randomized controlled trials
SOHO: Schizophrenia Outpatients Health Outcomes
WHO ITCRP: WHO International Clinical Trials Registry Platform
WHO: World Health Organization
