# Supplementary material for: The “RCT augmentation”: a novel simulation method to add patient heterogeneity into phase III trials
Source: BMC Med Res Methodol. 2018 Jul 6;18:75. doi: 10.1186/s12874-018-0534-6 (PMC6035409; doi:10.1186/s12874-018-0534-6)
Supplement: Supplementary file 1 — 1. Details on methods, 2. details on results, 3. comparison of the study results and the data from literature, 4. codes used for the analysis, modeling and simulation, and 5. references used in the Additional file 1. (DOCX 2465 kb) [file 12874_2018_534_MOESM1_ESM.docx]

## Additional file 1

## Details on Methods

### Definition of MSE and bias for model predictions

Model predictions obtained from different datasets (using the same model) were evaluated using the meansquared error (MSE):

$$MSE=\frac{1}{n}\sum_{i=1}^{n} \left( predicted \Delta{CGIS}_{i}-observed {\Delta CGIS}_{i} \right)^{2}$$

where n indicates the number of patients in the full SOHO population,

predicted ΔCGI-S is ΔCGI-S predicted based on the augmented RCT population data,

observed ΔCGI-S as measured in the SOHO trial (i.e., real-world ΔCGI-S).

MSE can also be rewritten:

$$\mathrm{MSE}={bias}^{2}+variance(predictor of \Delta CGIS)$$

with

$$bias=\frac{1}{n}\sum_{i=1}^{n} (predicted \Delta{CGIS}_{i}-observed {\Delta CGIS}_{i})$$

and

$$variance\left( predictor of \Delta CGIS \right)=\sum_{i=1}^{n} \left( predicted \Delta{CGIS}_{i}-\frac{1}{n}\sum_{i=1}^{n} predicted \Delta{CGIS}_{i} \right)^{2}$$

Bias and MSE tend towards zero as prediction performance increases, i.e., as the distribution of predicted changes in symptoms in the real-world population approaches its actualdistribution as observed in the entire SOHO cohort.

### Calculation of comparative efficacy in RCT, augmented RCT and real-world populations

Virtual RCTs were simulated to estimate the comparative efficacy of D1 vs. D2, the two most prevalent drugs in the cohort. The RCTs were all assumed to be a parallel, 2-arm trial with patients treated with eitherone of the twodrugs.

First, an imputation method was used to generate the missing data on covariates in SOHO and enable matching of the patients initiating drug D1 with those initiating drug D2. The R package “Amelia”[1,2] using the bootstrap expectation-maximization algorithm was chosen for the imputation because it is simple and quick to runusing alarge number of variables.

For each simulated trial, the population was drawn from one of the following groups:

- “RCT population”, to estimate comparative efficacy of Phase III trials with standard exclusion criteria
- “augmented RCT population”, to estimate comparative efficacy of trials with one or two relaxed exclusion criteria
- SOHO, to estimate comparative effectiveness

And the main steps for calculating comparative efficacy (or effectiveness) were:

1. Sample 250 patients takingdrug D2 from the chosen population above,
2. Match to 250 patients takingdrug D1 from the chosen population aboveby applying the propensity score matching approach with all 24 available variables (the caliper was set to 0.25 standard deviations of propensity score, as recommended by Rosenbaum and Rubin),[3] using the R package “MatchIt” [4]
3. Calculate the average ∆CGI-S between the paired groups of patients, i.e., the point estimate for the endpoint of the virtual trial.
4. Repeat the first three steps 1000 times (N=1000) and report the average CGI-S each time to obtain the distribution for the chosen population, which is represented in Fig 4

### Predictive model

An ordinal logistic model was developed to predict effects in the real world using data from the RCT population alone. First, the categorical variable CGI-S, which can assume values in {1,2,3,4,5,6,7}, was transformed with help of seven latent variables α_1,_ …, α_7_, defined as:

*CGI-S = j, if α_j-1_ <U≤ α_j_,with U ~ Uniform(0,1).*

In other words, α_j_ were defined as cumulative probabilities *α_j_ = p(CGI-S≤j).*

Second, the latent variables *α_j_* were modeled as a function of the explanatory variables ${\boldsymbol{x}=\{x}_{1},x_{2},\ldots, x_{n}\}$ with using a logistic regression:

$${\mathrm{logit} (\alpha_{j}\left( \boldsymbol{x} \right)) = \log( \frac{\alpha_{j}\left( \boldsymbol{x} \right)}{1-\alpha_{j}\left( \boldsymbol{x} \right)}) = \theta}_{j}+\beta_{1}x_{1}+\beta_{2}x_{2}+\cdots+\beta_{n}x_{n},$$

where $\theta_{j}$ is the intercept for the j-th latent variable$\alpha_{j}\left( \boldsymbol{x} \right)$,β_1_, β_2_,…, β_n_ are regression coefficients, ${\boldsymbol{x}=\{x}_{1},x_{2},\ldots, x_{n}\}$ is the vector of $x_{i}$variables, which were here all the variables available in the SOHO database, and n is the number of explanatory variables (n=24 in our case). The parameters {$\theta_{1}, \ldots, \theta_{7},$ β_1_, β_2_,…, β_24_} were jointly fitted to the RCT population data using the R function *polr()*.

The 24 variables $x_{i}$ are detailed below.

- Patient characteristics
  - Age, gender, BMI (body mass index), illness duration
  - Country of residence, practice type, work status, housing condition, social activities, relationships, number of suicide attempts
  - Hospitalization(binary), number of hospital admissions
  - EQ-depression, EQ-mobility, EQ-pain/discomfort, EQ- Self-care, EQ-usual activities and overall QOL score
  - Patient compliance
- Baseline symptoms
  - Negative symptom at baseline
  - Positive symptom at baseline
  - Cognitive symptom at baseline
  - CGI-S at baseline
- Drug dose prescribed at baseline
  - Dosage DDDeq

Concerning the choice of variables, first all continuous and categorical variables collected in the SOHO study were included. Then, variables were chosen by excluding multicollinearity among them with the help of the variance inflation factor (VIF). The VIF provided an index that measured how much the [variance](https://en.wikipedia.org/wiki/Variance) (i.e., the square of the [standard deviation](https://en.wikipedia.org/wiki/Standard_deviation) of theCGI-S estimate) of an estimated regression coefficient was increased because of collinearity. In practice, we calculated the VIF for each variable, and excluded the variablefrom the regression model if the corresponding VIF was superior to 5. In our case, all the included variables were kept as all the VIFs were inferior to 3.

The model predictions were carried out using the entire SOHO population baseline values without excluding the RCT population data (training set), becausethe goal of our analysis was to predict the drug effect in a wide population. Moreover, if we excluded the training set, the model predictions would not be based on the same dataset for different augmentations, preventing comparison.

For the predictive model developed on the RCT population, the *α_j_* values were found to be:

α_1_ = 0.234; α_2_ = 0.459; α_3_ = 0.648; α_4_ = 0.823; α_5_ = 0.940; α_6_ = 0.992; α_7_ = 1.

## Details on Results

### Percentage of patients and change in observed CGI-S in different subpopulations initiating drugs D1or D2


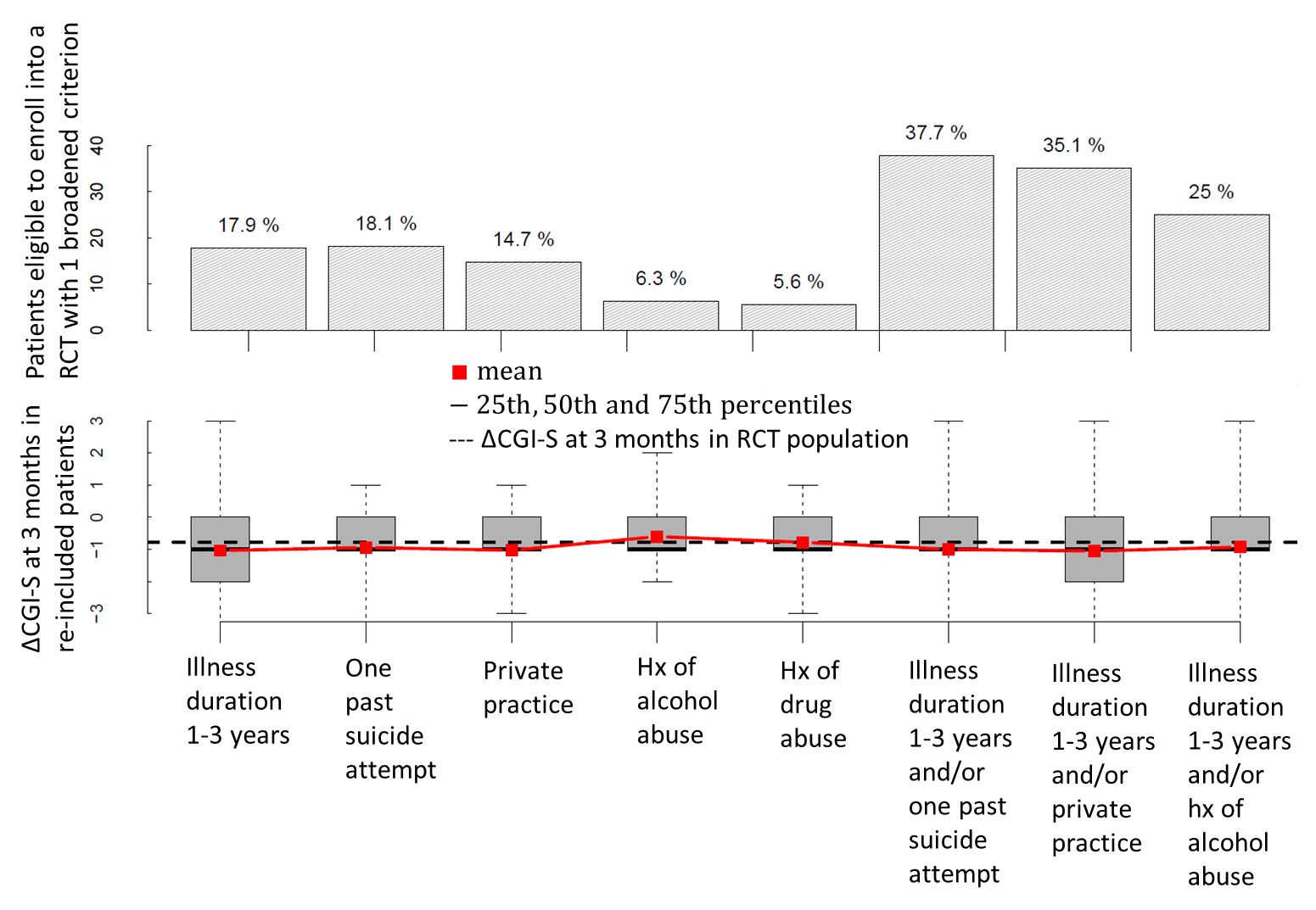


#### Fig. S1 Percentage of patients & change in symptoms across different subpopulations initiating drug D1

Notes: Red square (▪) represents the mean value of ΔCGI-S in each subpopulation.

Dashed line (**--**) represents the mean value of ΔCGI-S change in the “RCT population”.

Boxplot represents five summary statistics from top to down (maximum, third quartile, median, first quartile and minimum) of ΔCGI-S in each subpopulation.


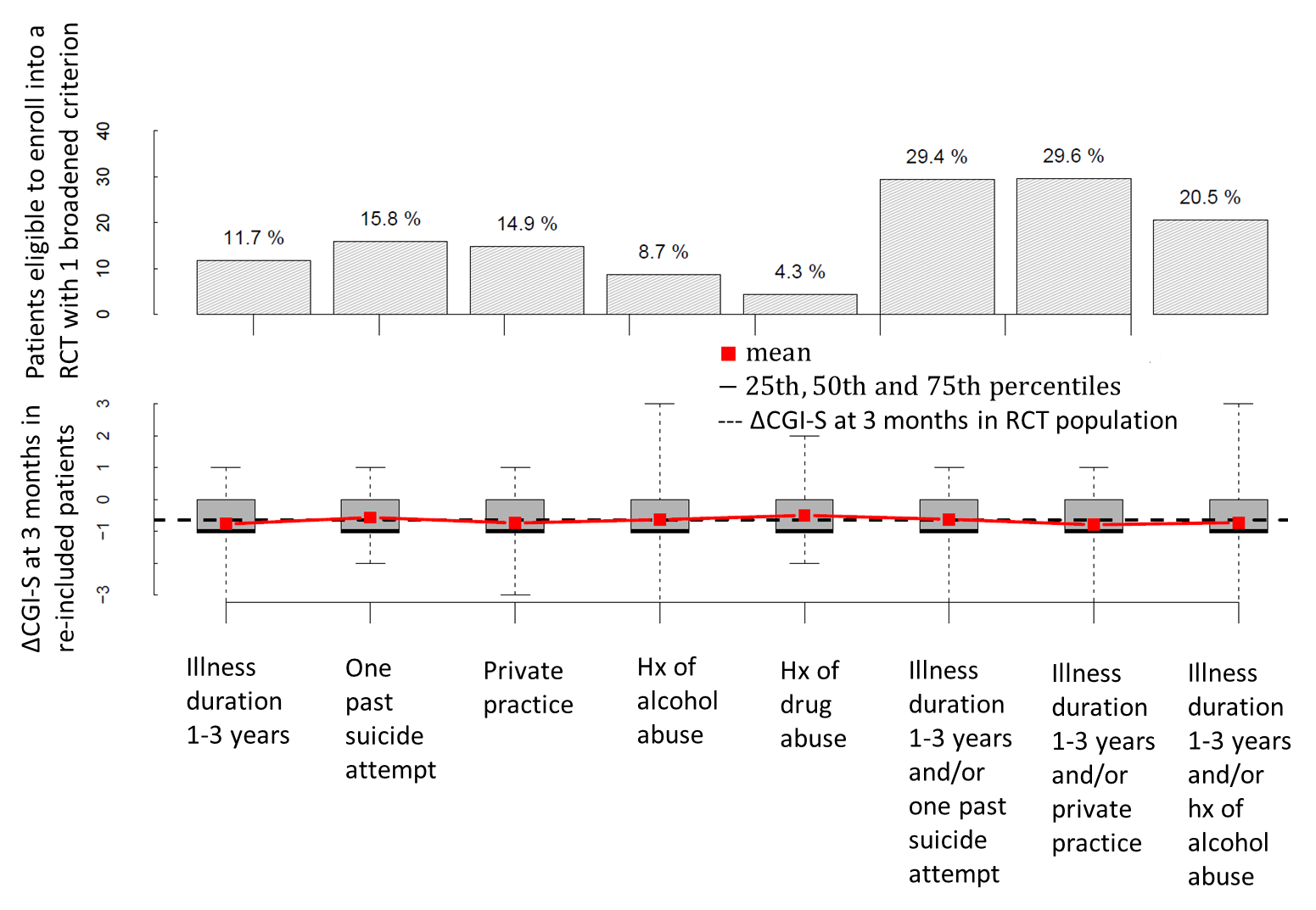


#### Fig. S2 Percentage of patients &change in symptomsin different subpopulations initiating drug D2

Notes: Red square (▪) represents the mean value of ΔCGI-S in each subpopulation.

Dashed line (**--**) represents the mean value of ΔCGI-S change in the “RCT population”.

Boxplot represents five summary statistics from top to down (maximum, third quartile, median, first quartile and minimum) of ΔCGI-S in each subpopulation.

### MSE from model fitted to data from augmented RCT populations of patients initiating drug D1– per augmentation factor


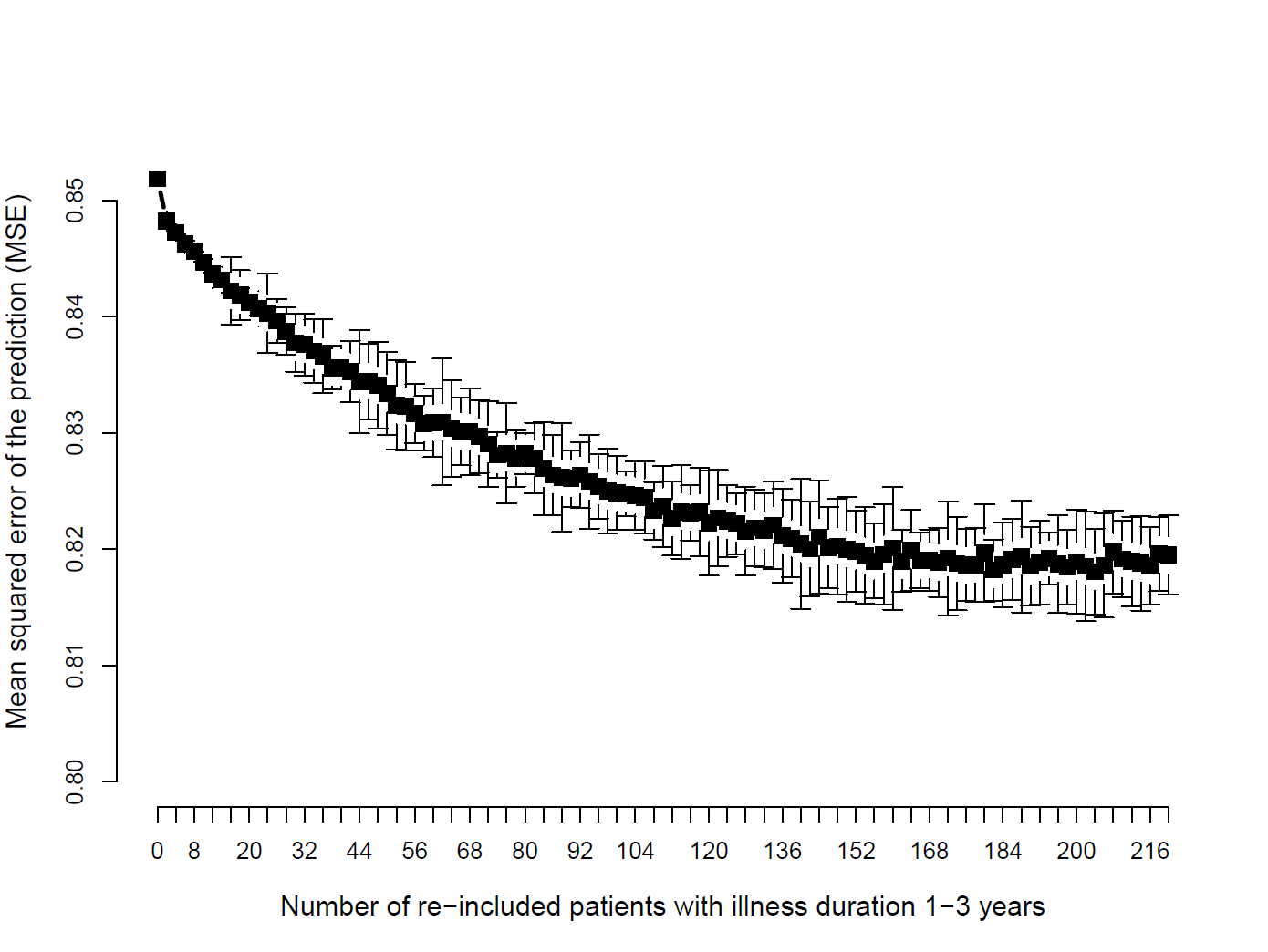


**Fig. S3** Prediction MSE - augmentation in patients with illness duration between 1-3 years. Each square represents an average of 500 random samplings of re-included patients; the interval delimits the 2.5% and 97.5% percentiles of the MSE for these 500 random samplings.
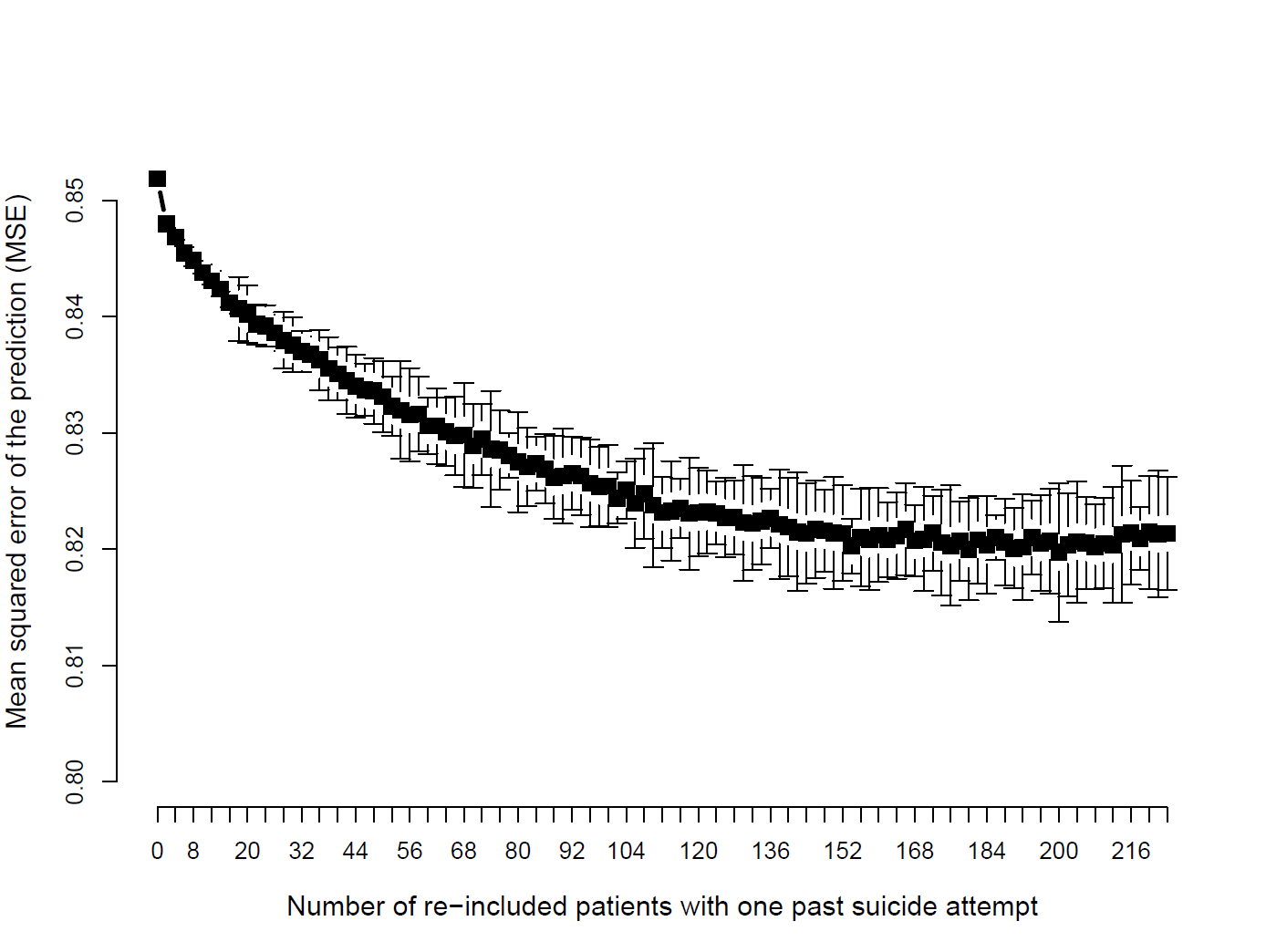


#### Fig. S4 Prediction MSE- augmentation in patients with one past suicide attempt. Each square represents an average of 500 random samplings of re-included patients; the interval delimits the 2.5% and 97.5% percentiles of the MSE for these 500 random samplings.


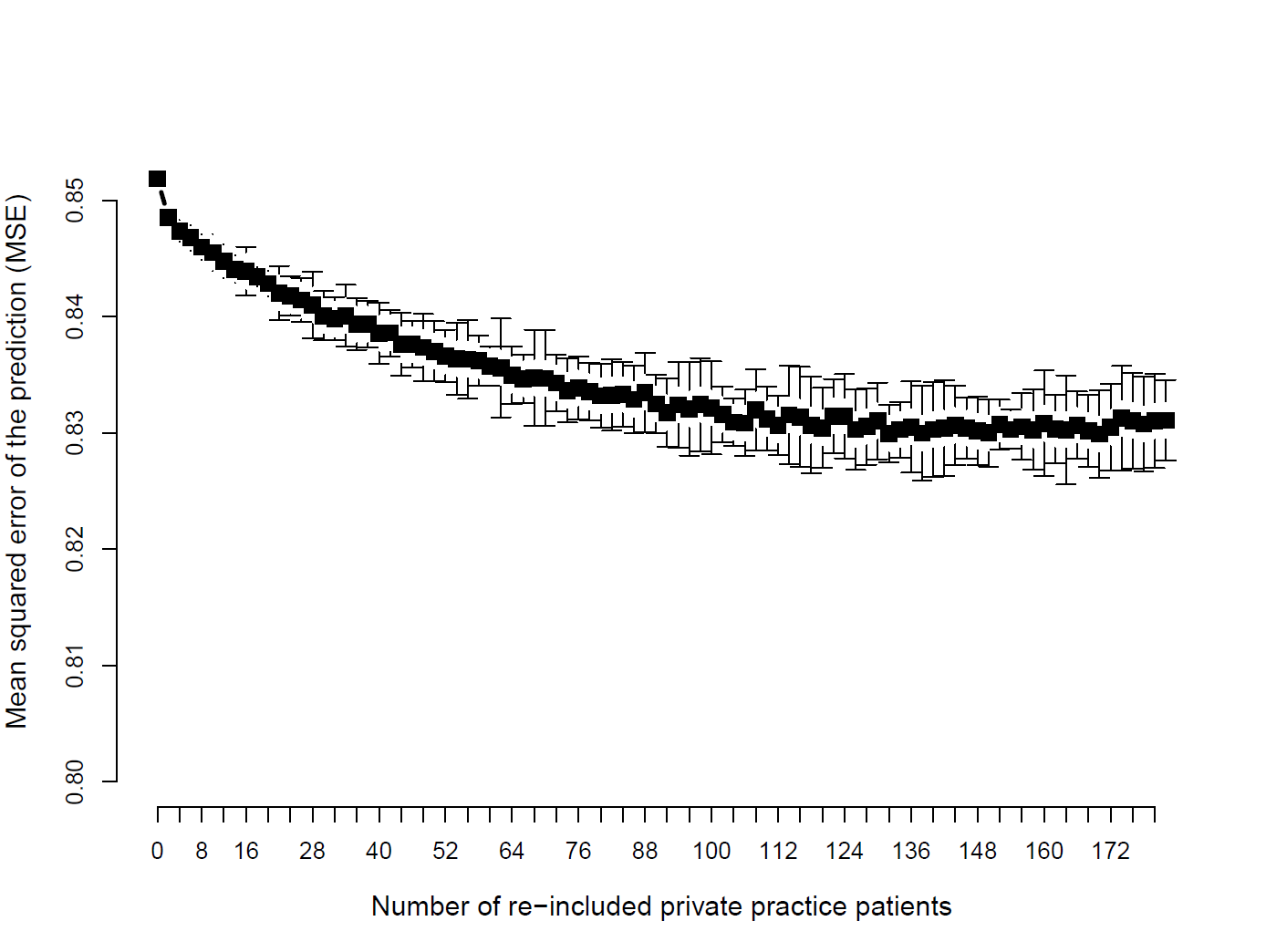


#### Fig. S5 Prediction MSE- augmentation in private practice patients. Each square represents an average of 500 random samplings of re-included patients; the interval delimits the 2.5% and 97.5% percentiles of the MSE for these 500 random samplings.

**
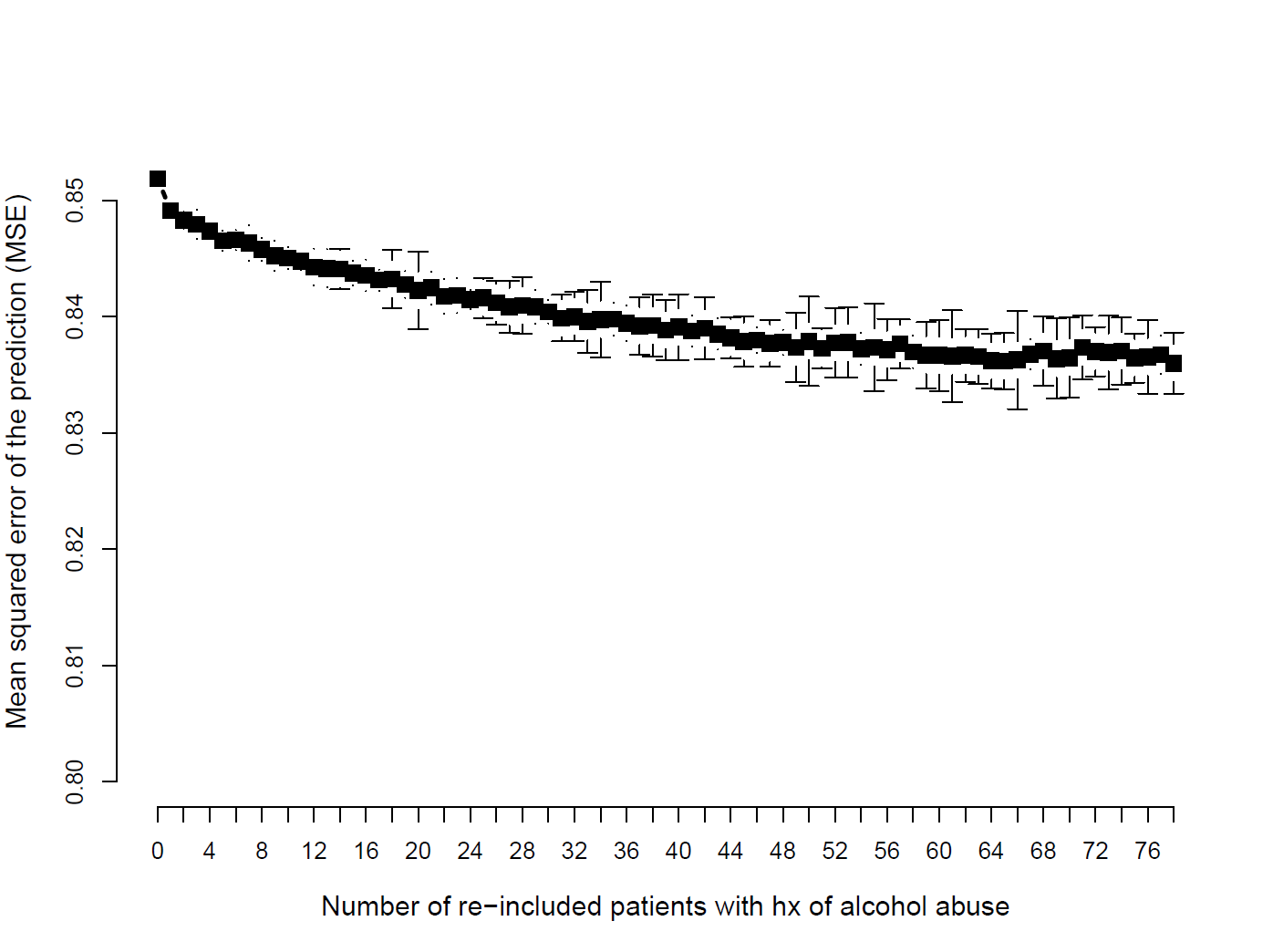
**

**Fig. S6** Prediction MSE- augmentation in patients with a history of alcohol abuse***.*** Each square represents an average of 500 random samplings of re-included patients; the interval delimits the 2.5% and 97.5% percentiles of the MSE for these 500 random samplings.


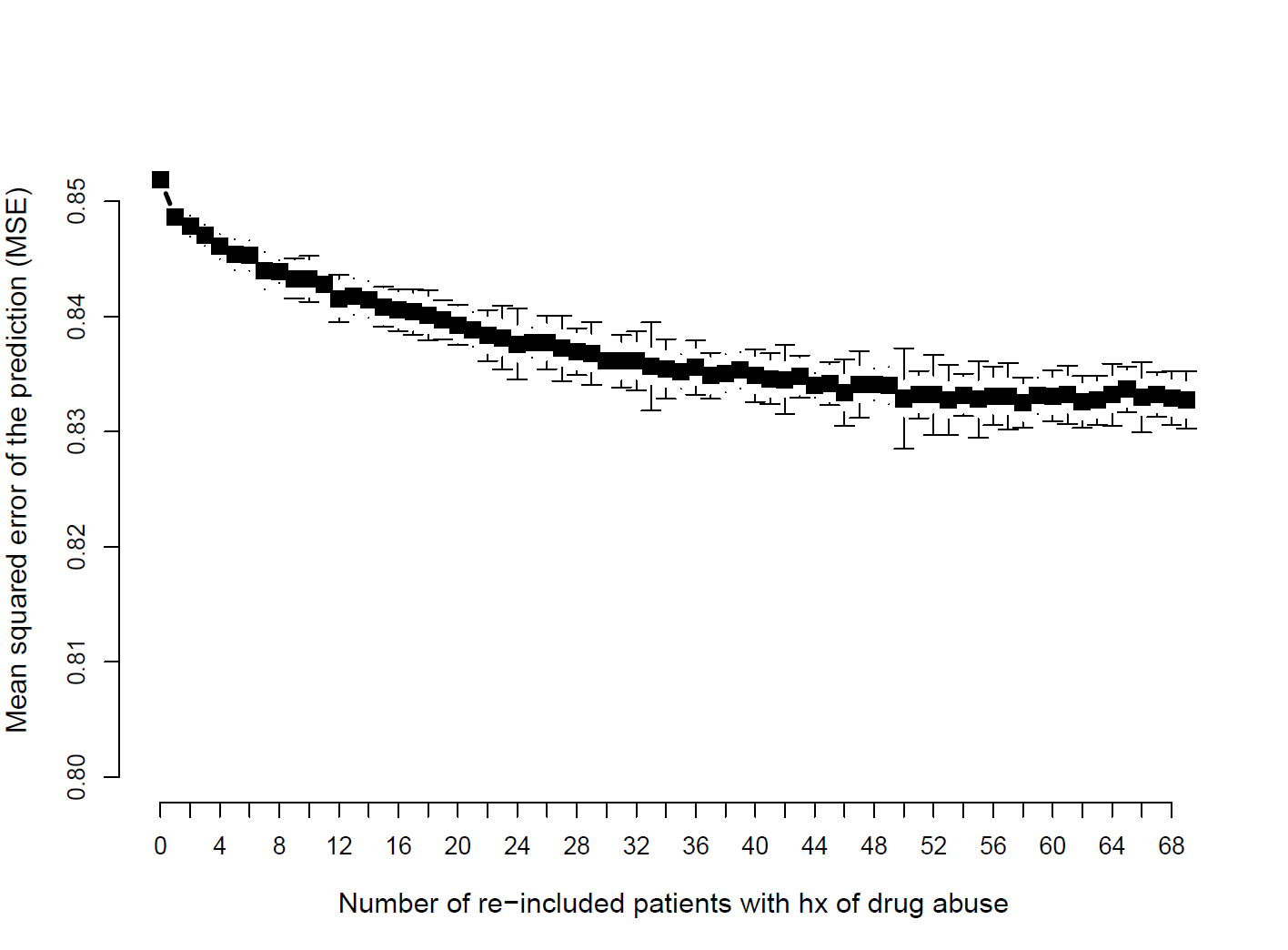


#### Fig. S7 Prediction MSE- augmentation in patients with a history of drug abuse. Each square represents an average of 500 random samplings of re-included patients; the interval delimits the 2.5% and 97.5% percentiles of the MSE for these 500 random samplings.


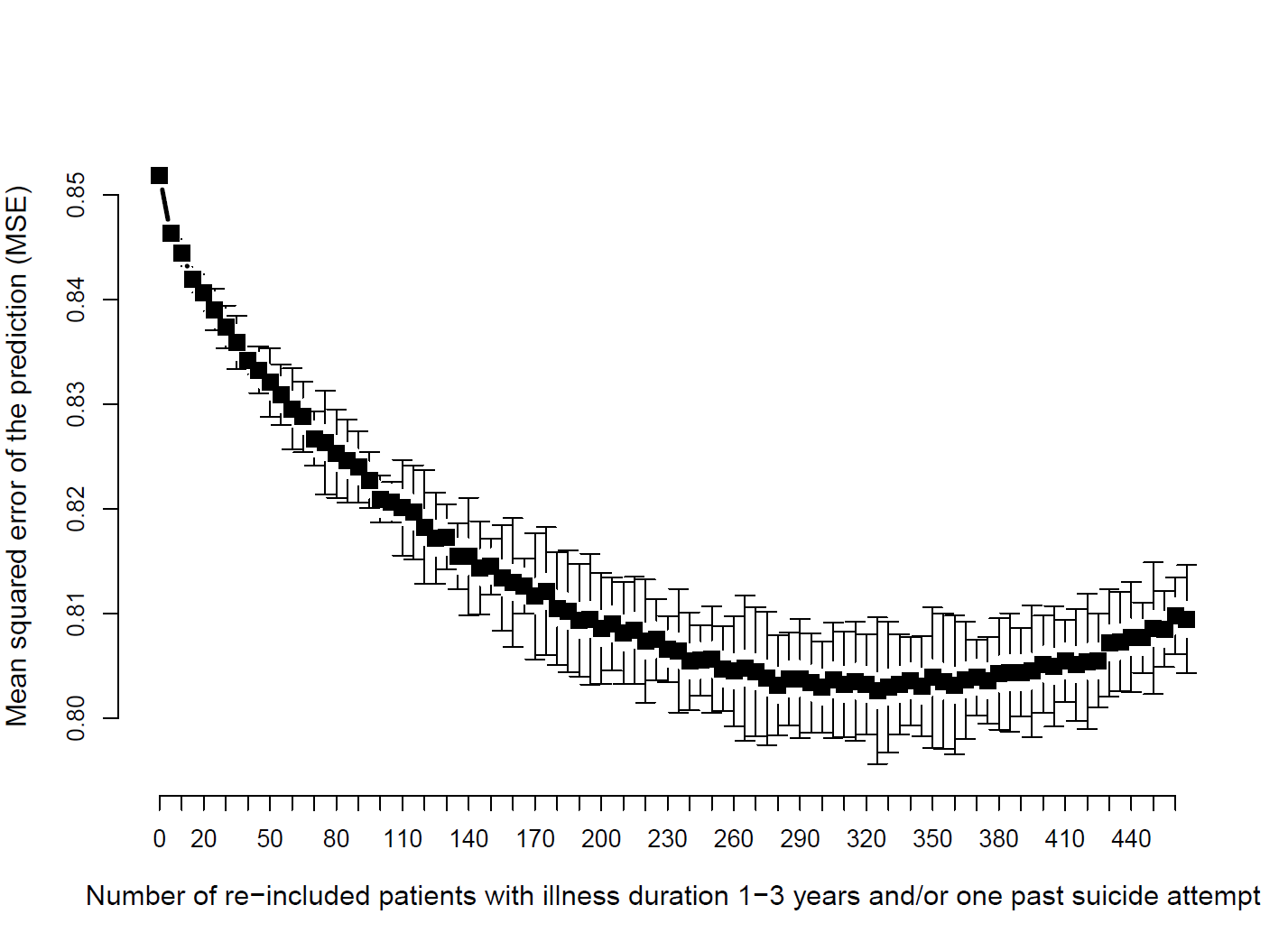


#### Fig. S8 Prediction MSE- augmentation in patients with illness duration between 1-3 years or one prior suicide attempt (or both). Each square represents an average of 500 random samplings of re-included patients; the interval delimits the 2.5% and 97.5% percentiles of the MSE for these 500 random samplings.

**
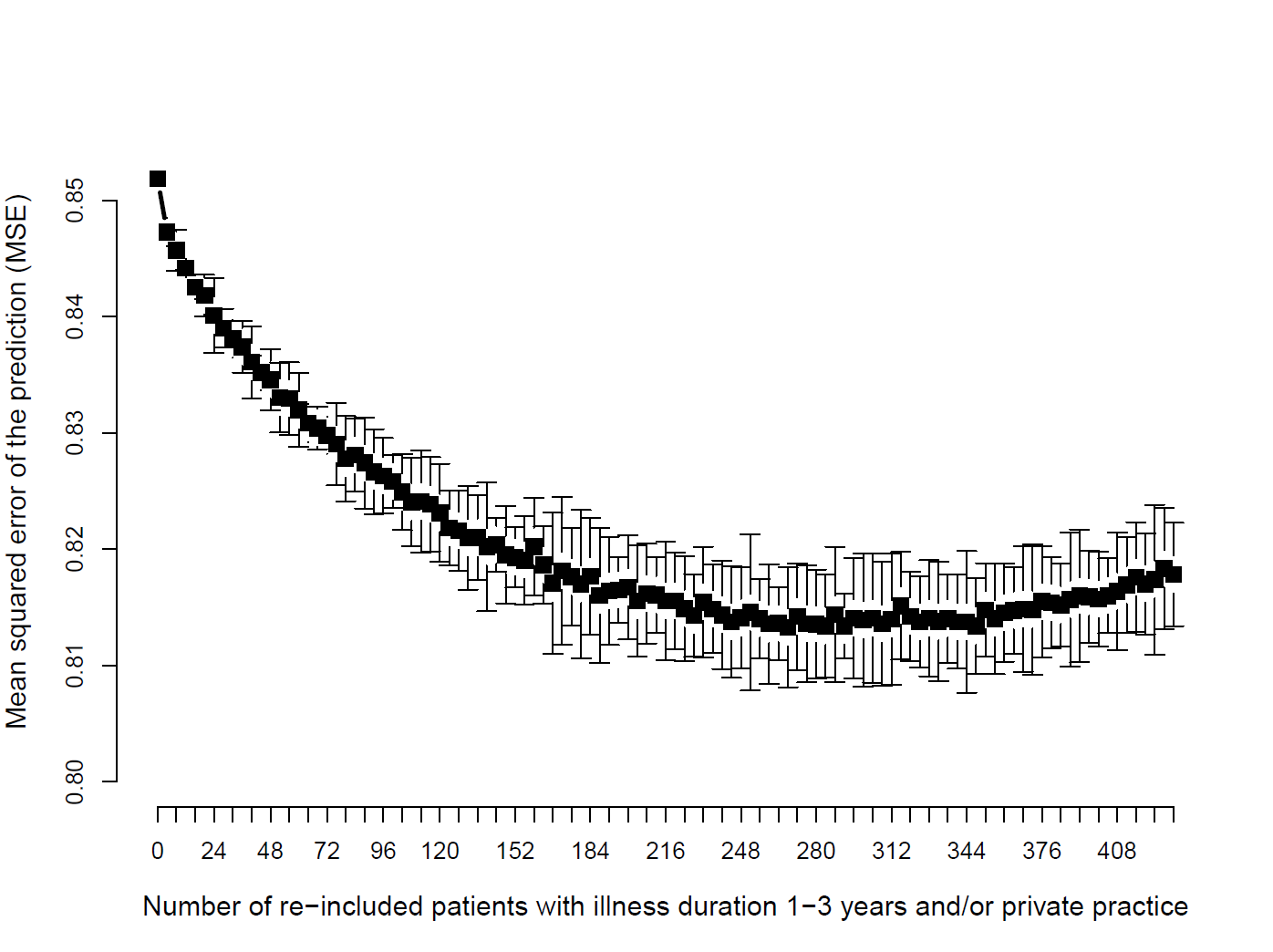
**

**Fig. S9** Prediction MSE- augmentation in patients with illness duration between 1-3 years or in private practice patients (or both). Each square represents an average of 500 random samplings of re-included patients; the interval delimits the 2.5% and 97.5% percentiles of the MSE for these 500 random samplings.

**
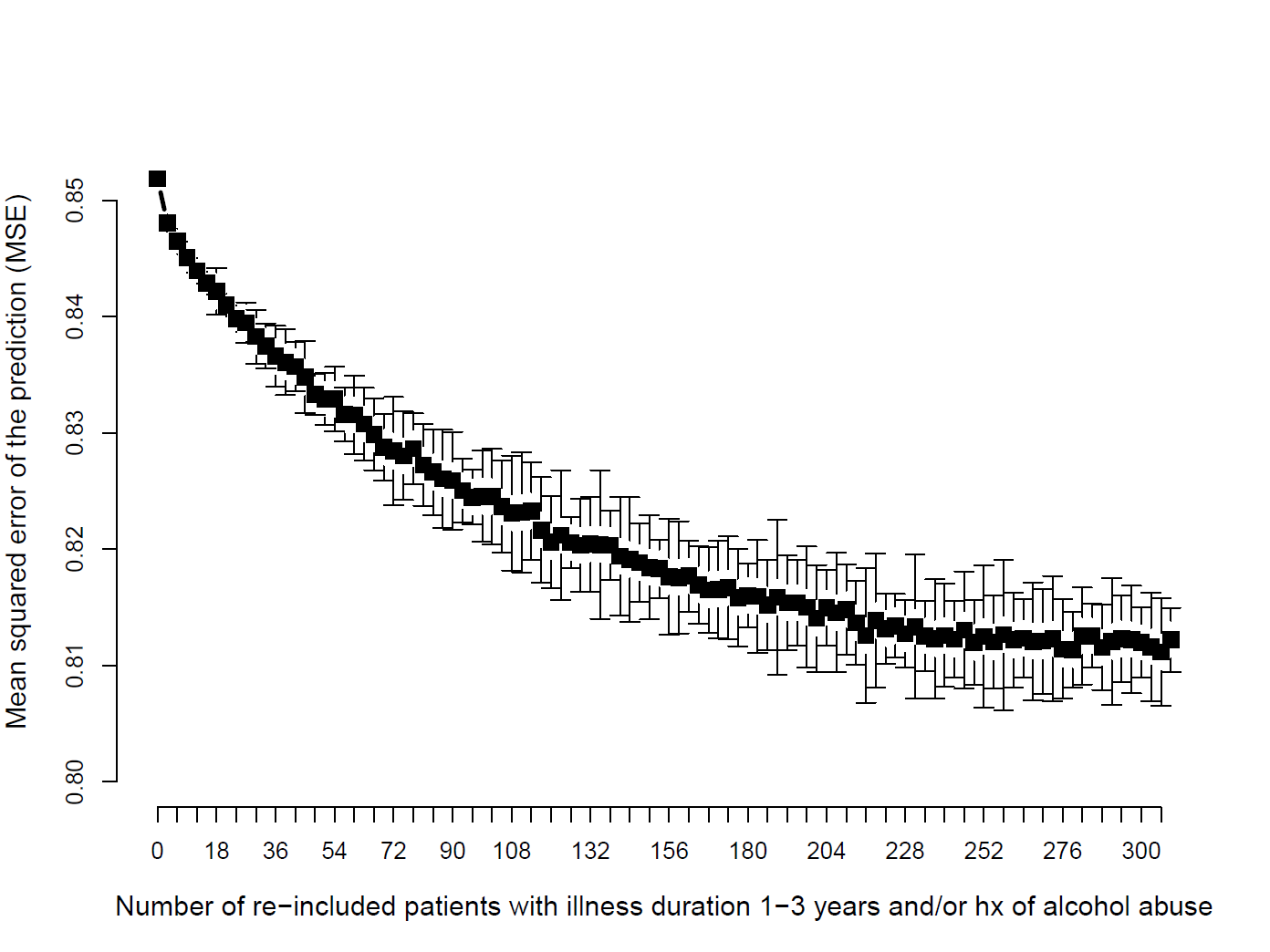
**

**Fig. S10** Prediction MSE- augmentation in patients with illness duration between 1-3 years or history of alcohol abuse (or both). Each square represents an average of 500 random samplings of re-included patients; the interval delimits the 2.5% and 97.5% percentiles of the MSE for these 500 random samplings.

### MSE from model fitted to data from augmented RCT populations of patients initiating drug D2 – per augmentation factor


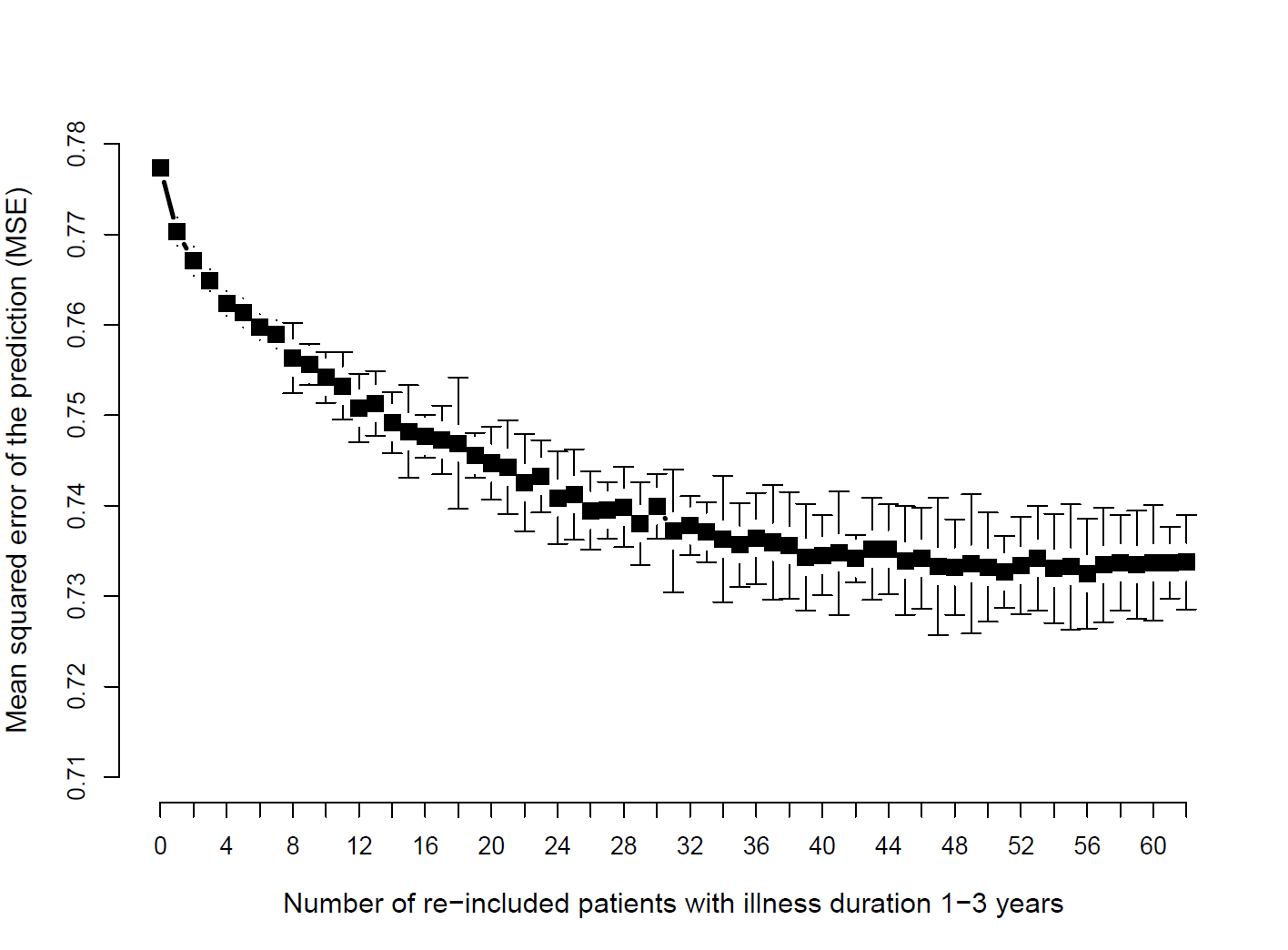


**Fig. S11** Prediction MSE- augmentation in patients with illness duration between 1-3 years. Each square represents an average of 500 random samplings of re-included patients; the interval delimits the 2.5% and 97.5% percentiles of the MSE for these 500 random samplings.

**
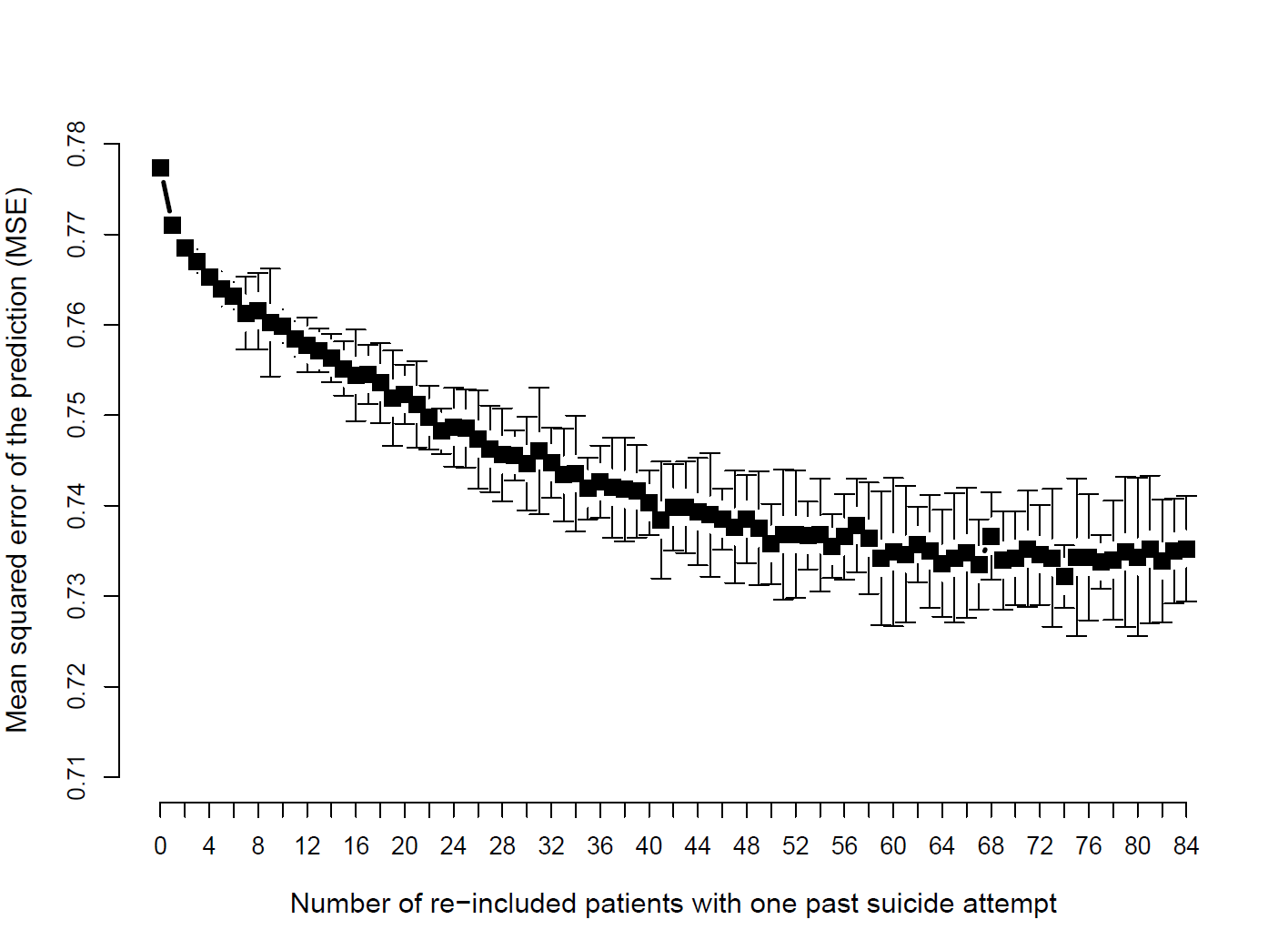
**

**Fig. S12** Prediction MSE- augmentation in patients with one past suicide attempt. Each square represents an average of 500 random samplings of re-included patients; the interval delimits the 2.5% and 97.5% percentiles of the MSE for these 500 random samplings.


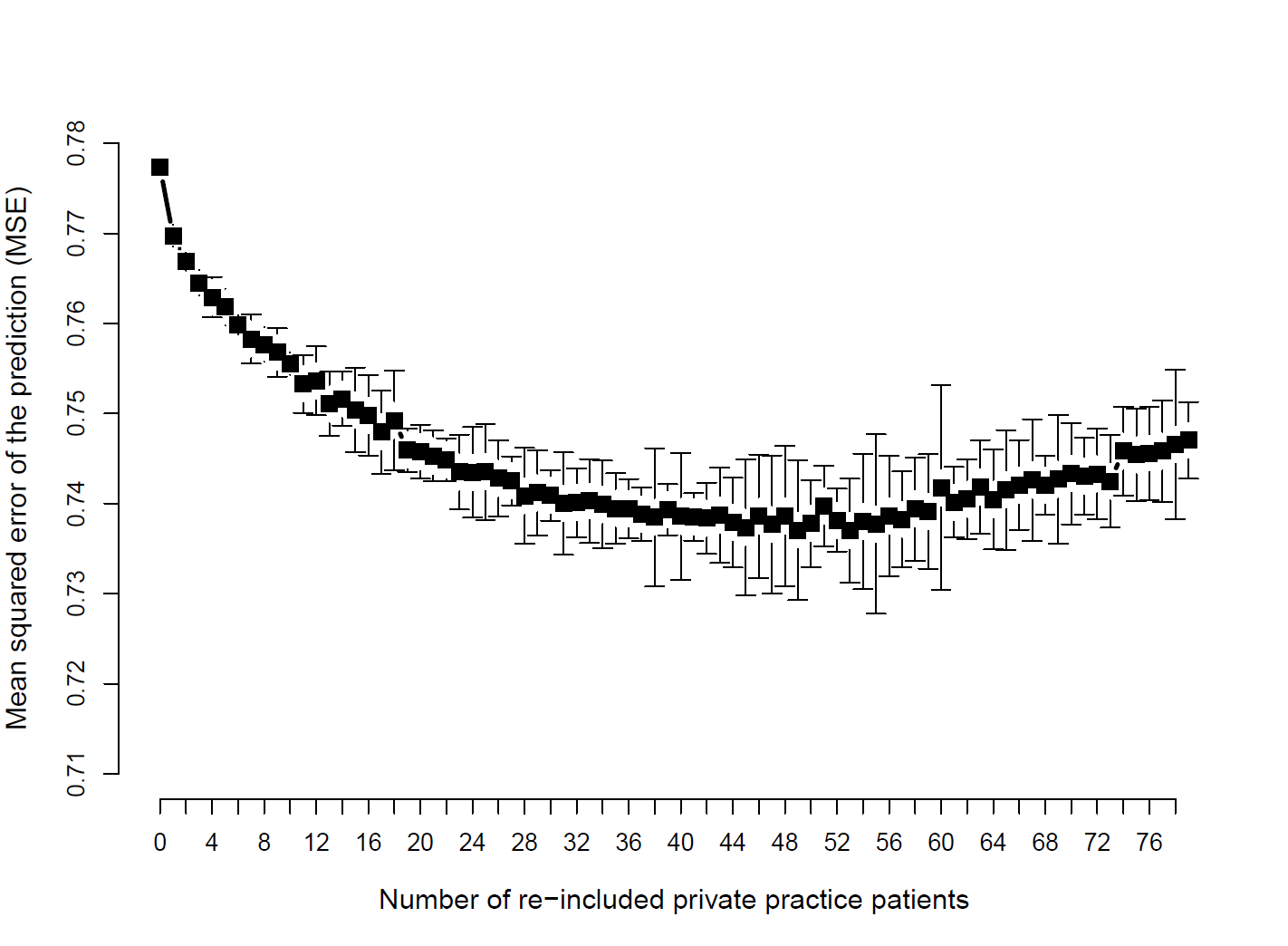


#### Fig. S13 Prediction MSE - augmentation in private practice patients. Each square represents an average of 500 random samplings of re-included patients; the interval delimits the 2.5% and 97.5% percentiles of the MSE for these 500 random samplings.


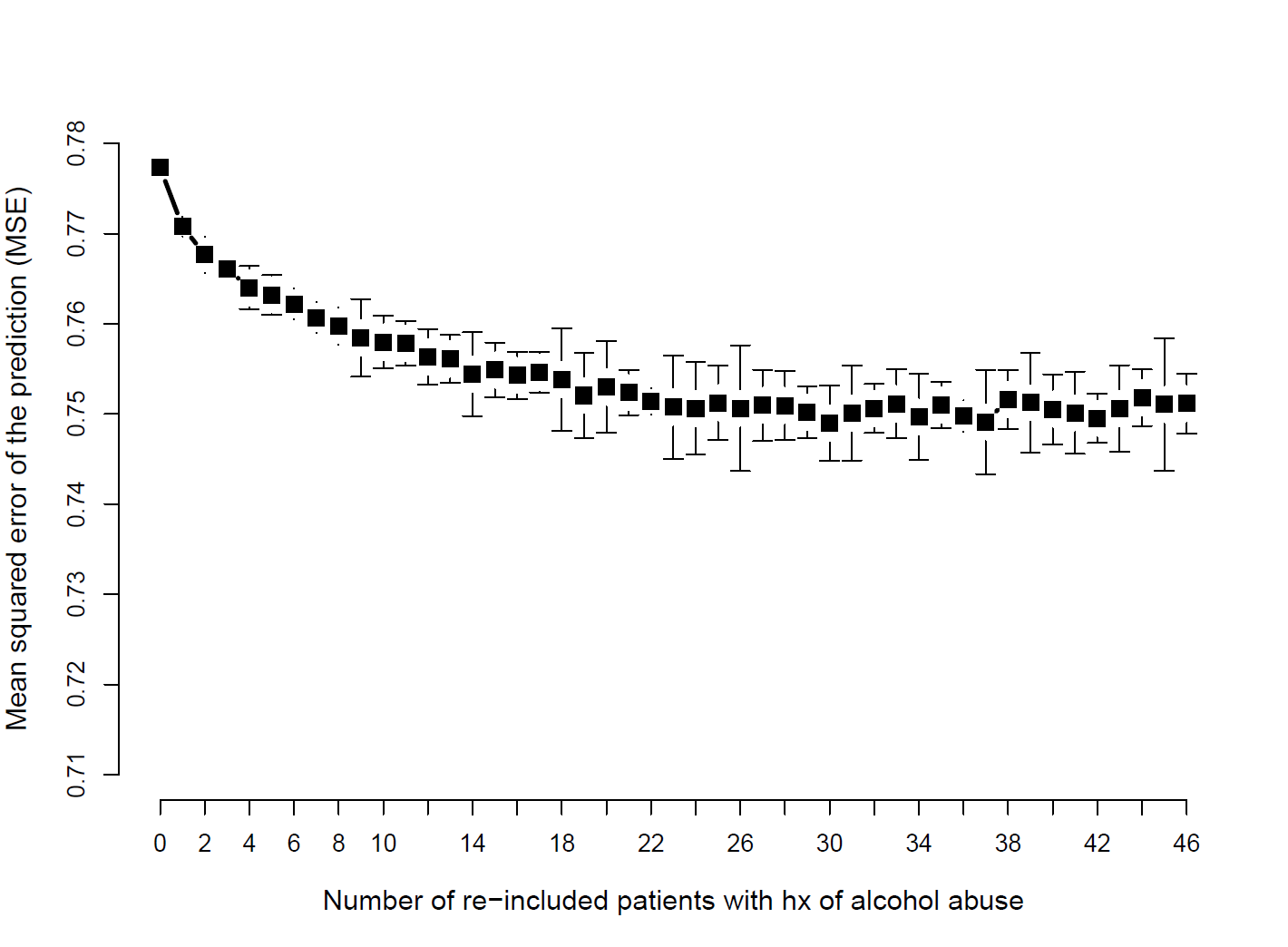


**Fig. S14** Prediction MSE - augmentation in patients with a history of alcohol abuse. . Each square represents an average of 500 random samplings of re-included patients; the interval delimits the 2.5% and 97.5% percentiles of the MSE for these 500 random samplings.

**
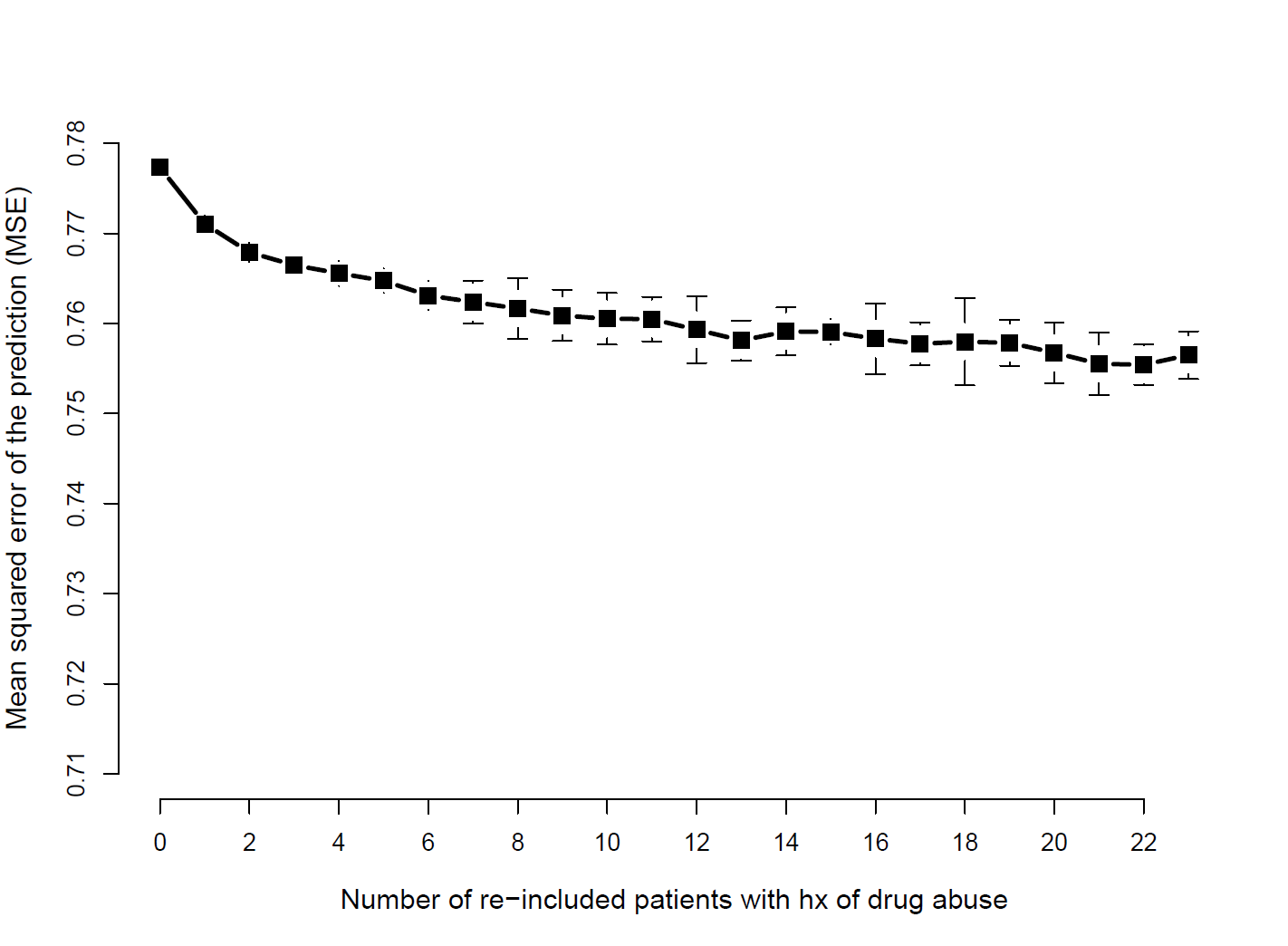
**

**Fig. S15** Prediction MSE- augmentation in patients with a history of drug abuse. Each square represents an average of 500 random samplings of re-included patients; the interval delimits the 2.5% and 97.5% percentiles of the MSE for these 500 random samplings.

**
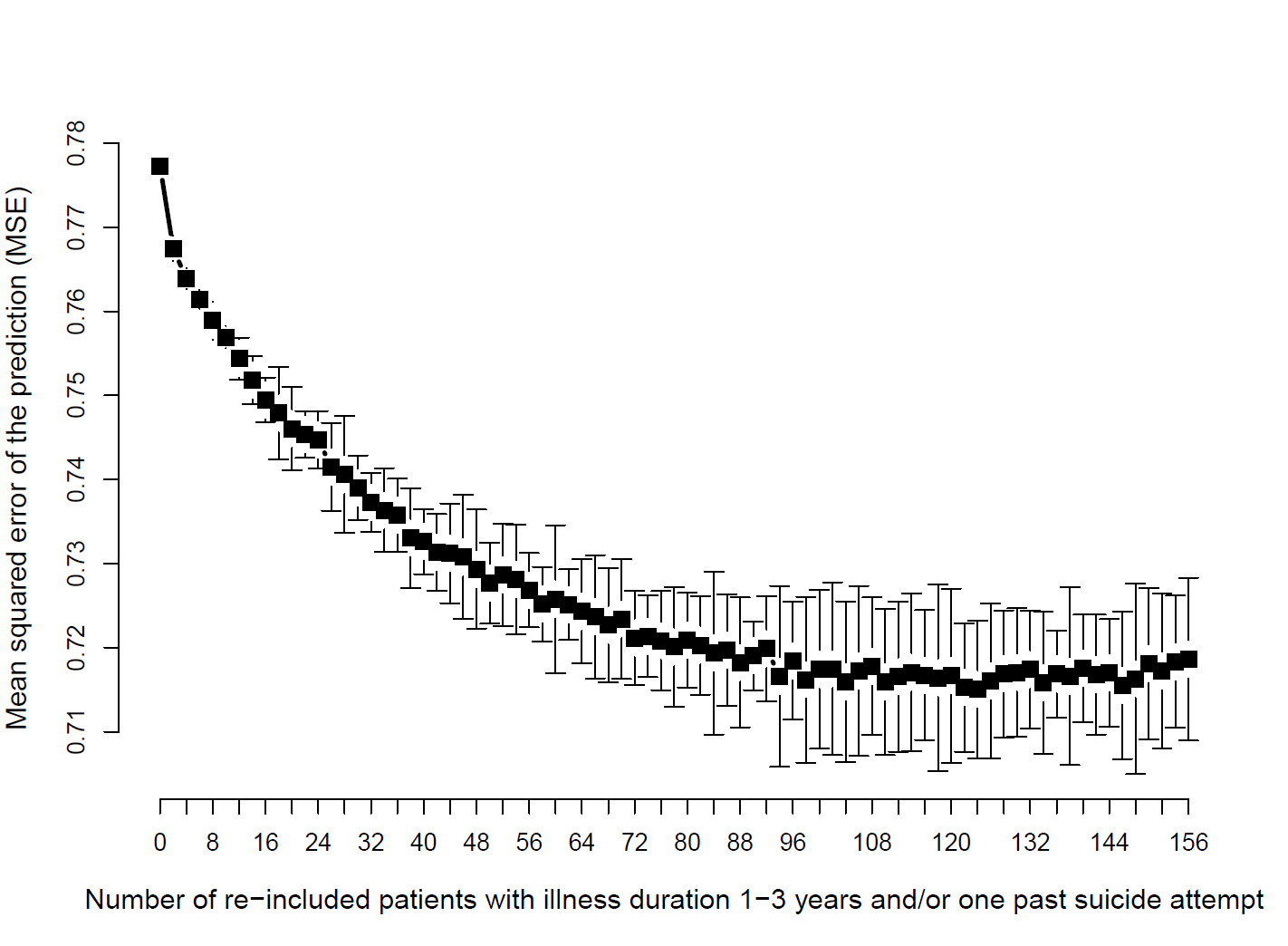
**

**Fig. S16** Prediction MSE- augmentation in patients with illness duration between 1-3 years or one prior suicide attempt (or both). Each square represents an average of 500 random samplings of re-included patients; the interval delimits the 2.5% and 97.5% percentiles of the MSE for these 500 random samplings.

**
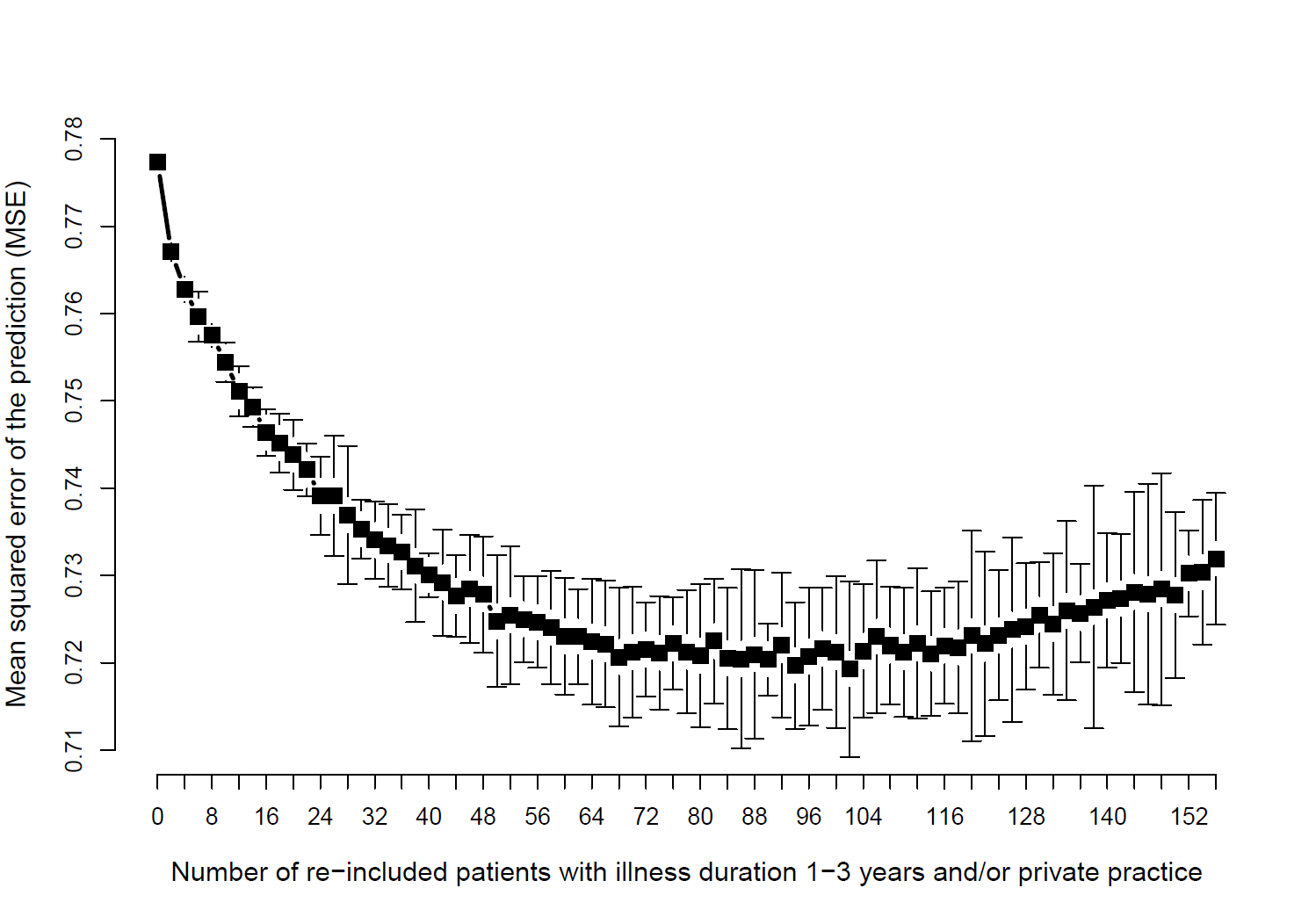
**

**Fig. S17** Prediction MSE- augmentation in patients with illness duration between 1-3 years or in private practice patients (or both). Each square represents an average of 500 random samplings of re-included patients; the interval delimits the 2.5% and 97.5% percentiles of the MSE for these 500 random samplings.


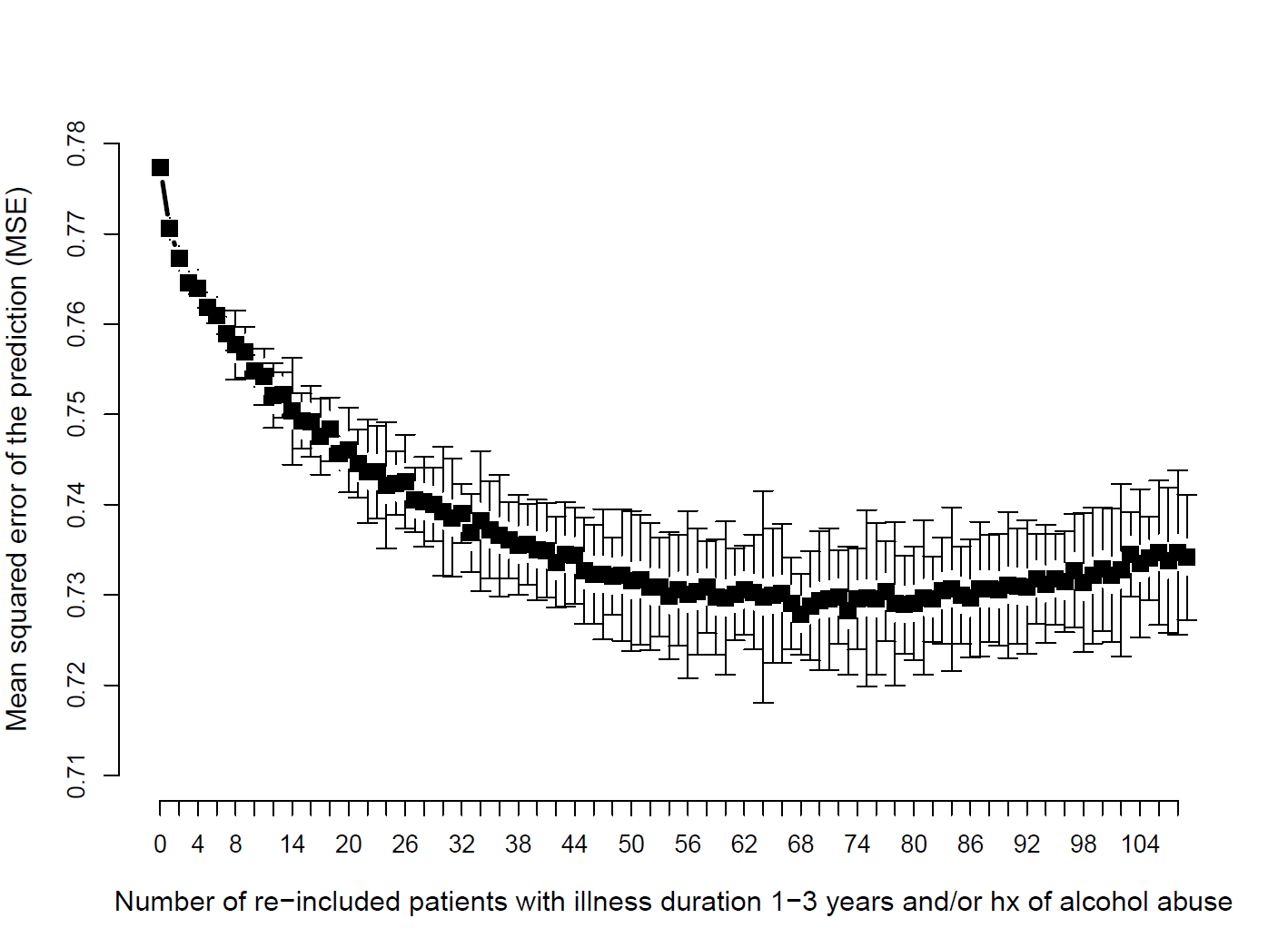


**Fig. S18** Prediction MSE- augmentation in patients with illness duration between 1-3 years or history of alcohol abuse (or both). Each square represents an average of 500 random samplings of re-included patients; the interval delimits the 2.5% and 97.5% percentiles of the MSE for these 500 random samplings.

### Results for drug D3, the third-most frequently initiated in SOHO

To allow for generalization of study results on schizophrenia, the analyses, modeling and simulations on absolute effectiveness were repeated for drug D3, the third-most frequently initiated drug in SOHO; the comparative efficacy over D1 was also calculated. The relative sizes of patient populations under D3 and the associated outcomes are displayed in Table S1; the results of prediction MSE are shown in Fig. S19; the impact of relaxing different eligibility criteria in patients taking D3 is displayed in Table S2. Comparative efficacy between D1 and D3 is shown in Fig. S20 and Table S3. Please note that virtual Phase 3 trials used to calculated comparative efficacy of D3 vs. D1 compared only 200 matched patients in each treatment arm, instead of 250 matched patients for the comparison of D1 and D2, because of the reduced number of patients initiating drug D3 in SOHO.

Conclusions on drugs D1 and D2 generally held true also for drug D3.

- About a quarter (26%) of the SOHO real-world population initiating drug D3 met all six eligibility criteria required for the “RCT population”, a result similar to that of patients included in RCT populations taking D1 or D2.
- The model applied on RCT population data under drug D3 also returned accurate predictions for real-world effects. Namely, prediction bias represented less than 3% of the average real-world results for CGI-S at 3 months (0.098/3.80 =2.6%), while the MSE of the prediction represented about 7% of the average squared real-world CGI-S values at 3 months (1.034/14.44 = 7.2%). Every augmentation of the “RCT population” initiating drug D3 by relaxing any of the pre-selected eight exclusion criteria led to improvement of the prediction of real-world effects in terms of MSE (bias remained small). Equally, re-including patients with illness duration between 1-3 years or one past suicide attempt or only with one past suicide attempt yielded the most accurate prediction (lowest MSE at natural augmentation).
- The average comparative efficacy approached the real-world comparative effectiveness upon augmentation though re-introduction of specific real-world patients except those with a history of alcohol abuse. Variability in comparative efficacy with different augmentations varied between that of RCT and SOHO.

Table S1. Relative sizes of patient populations and outcomes across different populations: real-world, RCT, and subpopulations used for re-inclusion into the RCT by relaxing one given exclusion criterion in patients under drug D3

| **Population type** | **% increase in RCT-eligible patient pool (95% CI†)** | **Outcome**  **Average ΔCGI-S (95% CI‡)** |
| --- | --- | --- |
| **Patients initiating drug D3** | | |
| Real-world population (SOHO cohort) | **-** | -0.59 (-0.66, -0.52) |
| “RCT population”**(26.0% of SOHO cohort patients)** | 0 | -0.53 (-0.66, -0.40) |
| Patients with illnessduration between 1-3 years* | 12.2% (8.0%, 18.1%) | -0.38(-0.67, -0.08) |
| Patients with one past suicide attempt* | 17.8% (12.5%, 24.9%) | -0.65(-0.97, -0.33) |
| Private practice patients* | 7.5% (4.4%, 12.2%) | -0.50(-1.19, 0.19) |
| Patients with history of alcohol abuse* | 12.7% (8.4%, 18.6%) | -0.46 (-0.88, -0.05) |
| Patients with history of drug abuse* | 2.8% (1.2%, 5.9%) | -0.60 (-1.31, 0.12) |
| Patients with illness duration between 1-3 years and/or one past suicide attempt** | 31.9% (24.2%, 41.8%) | -0.57(-0.79, -0.35) |
| Patients with illness duration between 1-3 years and/or private practice patients** | 22.1% (16.0%, 30.0%) | -0.48(-0.77, -0.19) |
| Patients with illness duration between 1-3 years and/or history of alcohol abuse** | 25.4% (18.7%, 34.0%) | -0.45 (-0.71, -0.19) |

*plus meeting the remaining 5 RCT eligibility criteria.

** plus meeting the remaining 4 RCT eligibility criteria.

†The Clopper-Pearson interval was used to calculate the 95% confidence interval; CI: confidence interval.

‡The confidence interval (CI) was calculatedunder the assumption that ΔCGI-S had a normal distribution.


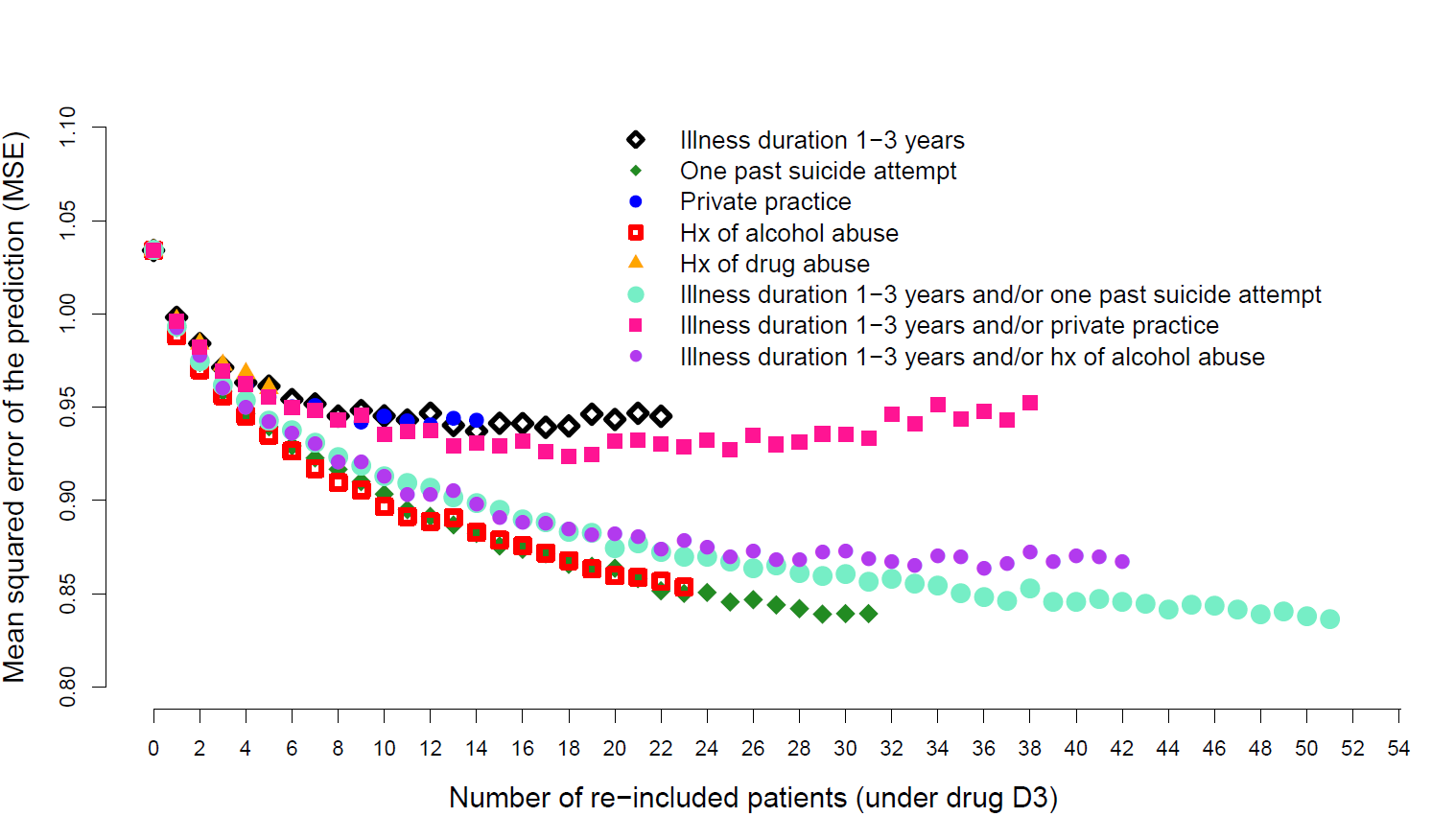


Fig. S19 Mean squared error of the prediction from model fitted to data using augmented RCT populations of patients initiating drug D3. The augmentation was performed by re-including, through random replacement within the RCT population, an increasing number of patients (x-axis) from eight different real-world subpopulations (colored markers) until the natural percentage of the patients with that specific characteristic was reached (right end of each curve). Each point represents an average of 500 random samplings of re-included patients.

**Table S2.** Comparison of the impact of relaxing different eligibility criteria in patients taking drug D3. Results for the “RCT population” and SOHO real-world populations are displayed as benchmark.

| Re-included subpopulations | | Natural augmentation (number of patients re-included when opening the trial to the specific real-world population subgroup) | Prediction bias with natural augmentation | Mean squared error (MSE) of prediction with natural augmentation |
| --- | --- | --- | --- | --- |
| Relaxed eligibility criteria | Illness duration between 1-3 years | 23 | 0.084 | 0.945 |
|  | One past suicide attempt | 32 | 0.073 | 0.840 |
|  | Private practice | 15 | 0.134 | 0.943 |
|  | Alcohol abuse | 24 | 0.072 | 0.854 |
|  | Drug abuse | 6 | 0.088 | 0.960 |
|  | Illness duration between 1-3 years + one past suicide attempt | 52 | 0.058 | 0.836 |
|  | Illness duration between 1-3 years + private practice | 43 | 0.126 | 0.952 |
|  | Illness duration between 1-3 years + alcohol abuse | 39 | 0.030 | 0.867 |
| RCT population | | n/a | 0.098 | 1.034 |
| SOHO real-world population | | n/a | 0.000 | 0.000 |

**Table S3.** Comparative efficacy of virtual RCTs comparing drug D1 and drug D3 in two parallel study arms with 200 patients each.

| Re-included subpopulations | | Average comparative efficacy of D1 vs. D2  (ΔCGI-S for D1 – ΔCGI-S for D2) | Standard deviation of comparative efficacy D1 vs. D2  (ΔCGI-S for D1 – ΔCGI-S for D2) |
| --- | --- | --- | --- |
| Relaxed eligibility criteria | Illness duration between 1-3 years | -0.204 | 0.055 |
|  | One past suicide attempt | -0.215 | 0.059 |
|  | Private practice | -0.184 | 0.055 |
|  | Alcohol abuse | -0.140 | 0.052 |
|  | Drug abuse | -0.149 | 0.052 |
|  | Illness duration between 1-3 years + one past suicide attempt | -0.255 | 0.064 |
|  | Illness duration between 1-3 years + private practice | -0.226 | 0.063 |
|  | Illness duration between 1-3 years + alcohol abuse | -0.186 | 0.061 |
| RCT population | | -0.149 | 0.047 |
| SOHO real-world population | | -0.193 | 0.085 |


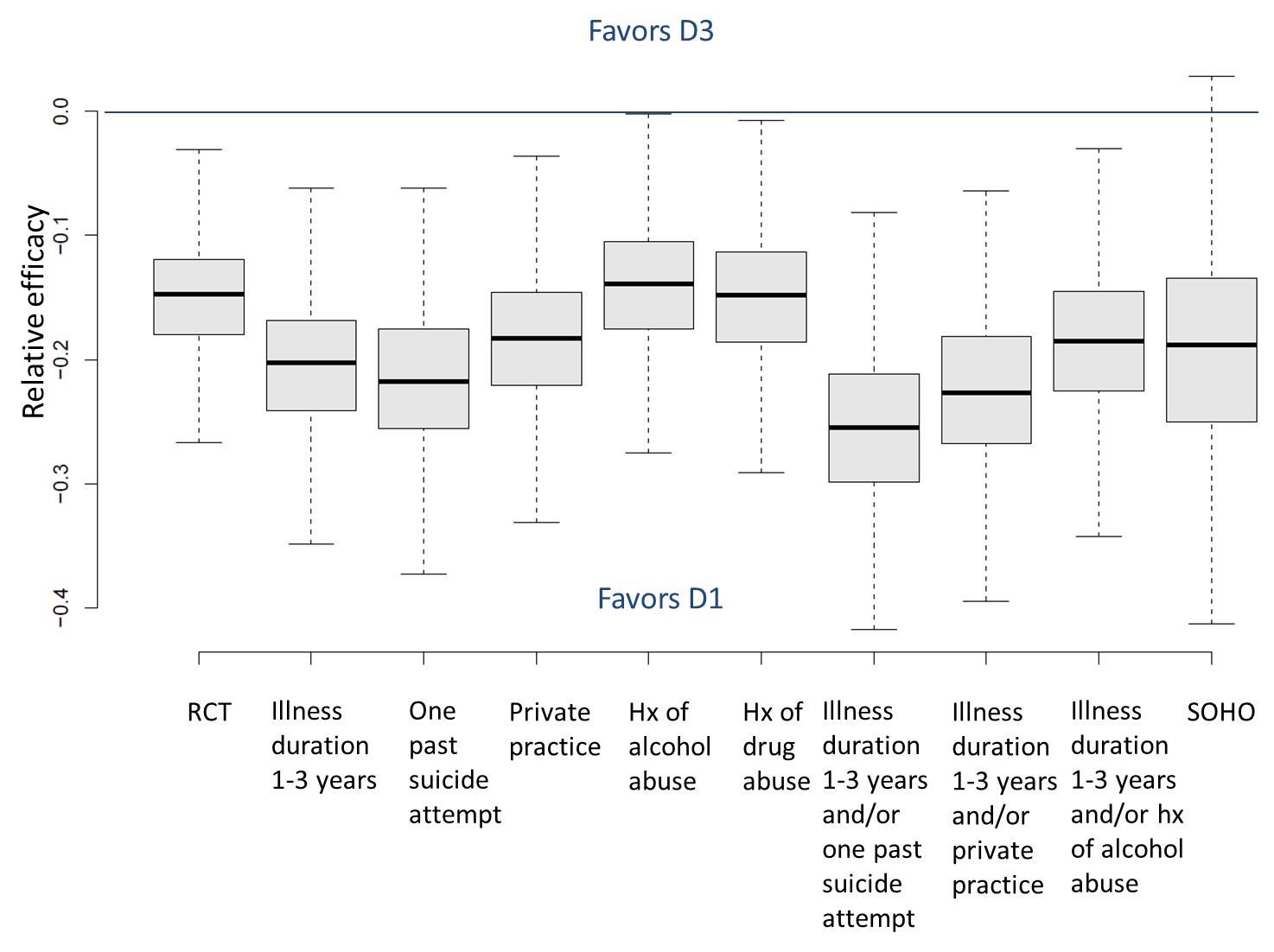


**Fig. S20** Comparative efficacy of virtual RCTs comparing drug D1 and drug D3 in two parallel study arms with 200 patients each. Source populations are displayed on the x-axis: RCT or augmented RCTs as a result of relaxing any of the eight eligibility criteria. Comparative effectiveness is reported for the real-world population, i.e. in the entire SOHO cohort.

### Bias from model fitted to data from augmented RCT populations of patients initiating drug D1, D2 or D3

Bias was calculated for all predictions as an additional test of the prediction quality, even though bias has already been implicitly considered in the more standard metric used to gauge prediction accuracy: MSE because $\mathrm{MSE}={bias}^{2}+variance(predictor)$ (see definitions at the top of Supplemental Material).

The prediction bias of models based on the “RCT population” represented less than 3% of the average real-world CGI-S at 3 months under all drugs D1, D2 or D3, whereas MSE of the prediction represented about 5-7% of the average squared real-world CGI-S values at 3 months. More precisely, the prediction bias was:

- - 0.054/3.54 = 1.5% of the average real-world CGI-S at 3 months under D1
  - 0.016/3.67 = 0.4% of the average real-world CGI-S at 3 months under D2
  - 0.098/3.8 = 2.6% of the average real-world CGI-S at 3 months under D3

Changes in bias upon augmentation (Fig. S21 below) differed for each drug and relaxed exclusion criterion. The difference in bias behavior upon augmentation between D1 (which was similar to D3) and D2 is attributable to the fact that predictions under D1 (and D3) had a positive prediction bias for the reference “RCT population” (x=0) (=model underestimated the absolute CGIS change), whereas predictions under D2 had a negative prediction bias (=model overestimated the absolute CGIS change, it ‘overshooted’). Therefore, under D2, bias reaches 0 faster upon augmentation, i.e., the absolute bias decreased faster than under D1 or D3, when the first patients of each characteristic were re-introduced. However, bias for predictions under D2 increased again as more patients with higher ΔCGI-S were re-introducted. For example, the patients with illness duration 1-3 years and/or one past suicide attempt (“the light blue curve” on Fig. S21b) have ΔCGI-S of -0.62 on average (see Table1), which raised the absolute bias value when reintroduced in their natural augmentation (right side of Fig. S21b).

a.


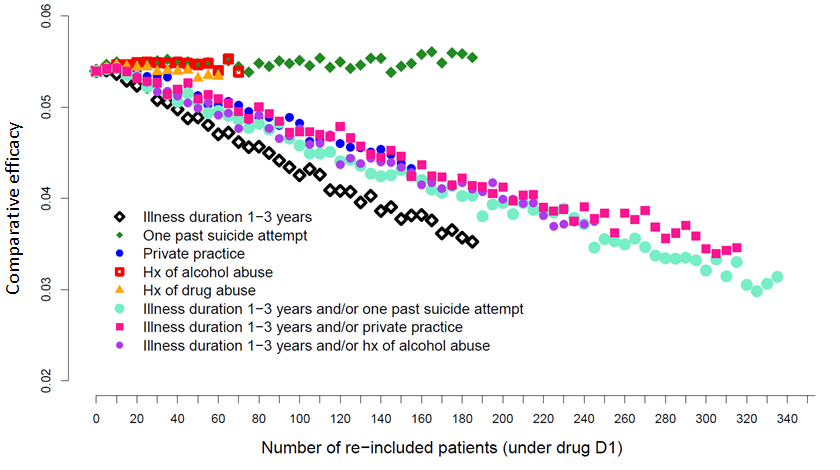


b.


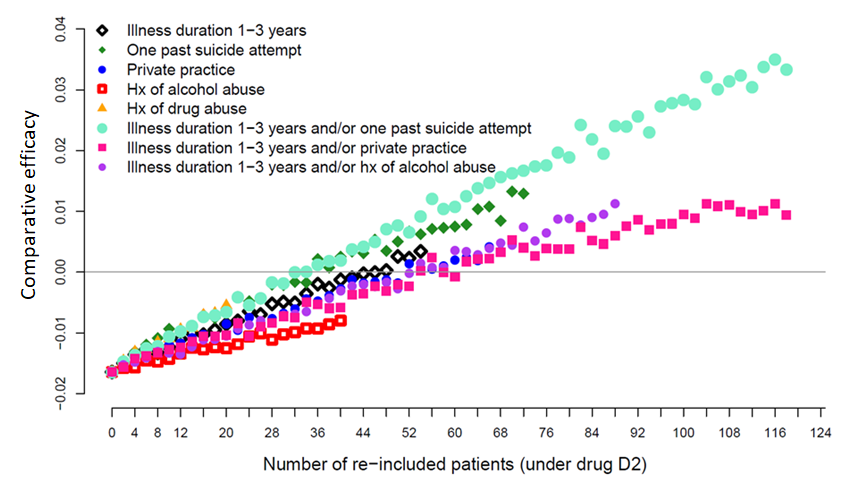


**c.**
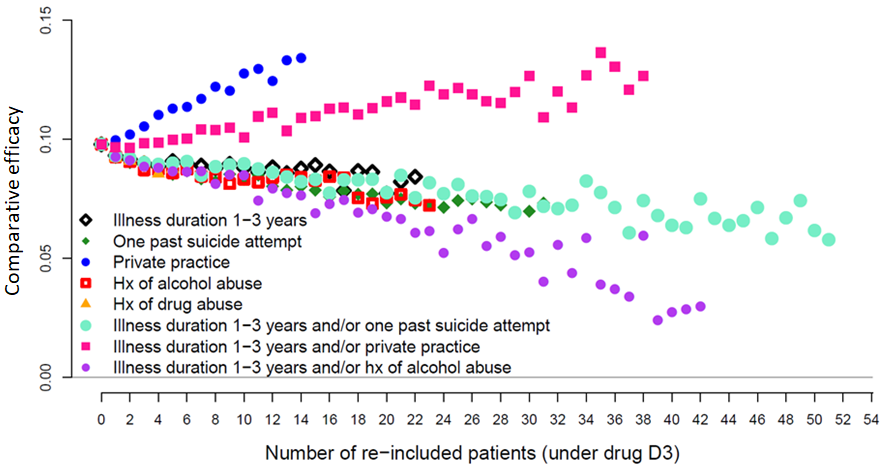


**Fig. S21** Bias of the prediction from model fitted to data using augmented RCT populations of patients initiating drug D1 (a), drug D2 (b), or drug D3 (c). The augmentation was performed by re-including, through random replacement within the RCT population, an increasing number of patients (x-axis) from eight different real-world subpopulations (colored markers) until the natural percentage of the patients with that specific characteristic was reached (right end of each curve). Each point represents an average of 500 random samplings of re-included patients.

## Comparison of CGI-S from the “RCT population” subset with values obtained for RCTs published in the literature

To validate the way the RCT populations were defined within SOHO, change in symptoms (ΔCGI-S) in these populations was compared to outcomes observed in RCTs for schizophrenia where participants were started on a new antipsychotic at baseline. A literature review returned six such schizophrenia studies where CGI-S was measured.[5-10] CGI-S at baseline and ΔCGI-S were extracted (Table S4). By comparing the CGI-S values (at baseline, changes between baseline and 3 months) in the “RCT population” with the CGI-S results from RCT studies, we noted that CGI-S values in the “RCT population” fell within a similar range to those from the six RCT studies identified in the literature, both for mean CGI-S values at baseline and ΔCGI-S between baseline and 3 months.

Table S4. Comparison of the CGI-S outcome in the “RCT population” carved out from the SOHO cohort and actual RCT populations reported in the literature

| Patients’data source[5-10] | Drug | CGI-S at baseline Mean (±SD) | ΔCGI-S between 3 months and baseline Mean (±SD) |
| --- | --- | --- | --- |
| “RCT population” as carved out from SOHO | drug D1 | 4.4 (±1.0) | -0.8 (±1.0) |
|  | drug D2 | 4.3 (±1.1) | -0.6 (±0.9) |
| RCT studies | | | |
| CATIE study [5] | Olanzapine | 4.0 (±1.0) | -0.4 (n/a) |
|  | Risperidone | 4.0 (±0.9) | -0.1 (n/a) |
|  | Ziprasidone | 3.9 (±0.9) | -0.1 (n/a) |
|  | Perphenazine | 3.9 (±1.0) | -0.3 (n/a) |
|  | Quetiapine | 3.9 (±0.9) | -0.2(n/a) |
| Korea prospective study [6] | Aripiprazole | 5.10 (±1.01) | -1.52 (n/a) |
| EUFEST study [7] | Olanzapine | 4.8 (±0.8) | -2 (n/a) |
|  | Haloperidol | 4.9 (±0.7) | -1.7 (n/a) |
|  | Ziprasidone | 4.8 (±0.8) | -1.9 (n/a) |
|  | Amisulpride | 4.8 (±0.8) | -2.3 (n/a) |
|  | Quetiapine | 4.9 (±0.8) | -1.8 (n/a) |
| Clinical PoC study [8] | Placebo | 4.4 (±0.6) | n/a |
|  | Bitopertin 10mg/d | 4.4 (±0.7) | n/a |
|  | Bitopertin 30mg/d | 4.6 (±0.8) | n/a |
|  | Bitopertin 60mg/d | 4.5 (±0.6) | n/a |
| COMETA study [9] | Naive patients | 4.6 (±0.9) | -0.7 (±1.2) |
|  | Non-naive patients | 4.3 (±1.1) | -0.2 (±1.1) |
| OLZ study 1 [10] | Olanzapine nonrelapser | 4.6 (±0.8) | -1.6 (±0.9) |
|  | Olanzapine relapser | 4.8 (±0.8) | -1.0 (±0.8) |
| OLZ study 2 [10] | Olanzapine nonrelapser | 4.8 (±0.7) | -1.8 (±1.0) |
|  | Olanzapine relapser | 4.9 (±0.7) | -1.8 (±0.7) |

Abbreviations: n/a, not available; SD, standard deviation

## Codes for analysis, modeling and simulations

We report below the codes used in the analyses, modeling and simulations performed on populations under drug D1, as well as comparative efficacy of D1 vs. D2. Codes to analyse populations under drug D2 and D3 were similar. Codes used to calculate comparative efficacy of drug D1 vs. D3 were similar to the ones to calculate the comparative efficacy of D1 vs. D2.

### Codes used to augment the RCT populations

###########################################################

#### Load data

###########################################################

#### Drug R = drug D1

#### Drug AE = drug D2

load("sohodata.RData")

nb.data <- dim(mydata)[1]

nb.var <- dim(mydata)[2]

nb.patient <- nb.data/4

### Inclusion and exclusion criteria are all based on variables at baseline

mydata.baseline <- mydata[seq(1,nb.data,4),]

mydata.3month <- mydata[seq(2,nb.data,4),]

###########################################################

#### RCT Creation

###########################################################

### Duration of illness superior to 3 years

### No history of alcohol abuse

### No history of drug abuse

### No history of suicide attempt

### Compliant patients

### Patients not included in private practices

inclu.flag <- rep(1, nb.patient)

R.flag <- AE.flag <- rep(0, nb.patient)

for(i in 1:nb.patient){

if(!is.na(mydata.baseline$R.after[i]) && mydata.baseline$R.after[i] >0) R.flag[i] <- 1

if(!is.na(mydata.baseline$AE.after[i]) && mydata.baseline$AE.after[i] >0) AE.flag[i] <- 1

if(is.na(mydata.baseline$duration[i]) | mydata.baseline$duration[i] < 3) inclu.flag[i] <- 0

if(is.na(mydata.baseline$never.alcohol[i]) | mydata.baseline$never.alcohol[i] != 1) inclu.flag[i] <- 0

if(is.na(mydata.baseline$never.substance[i]) | mydata.baseline$never.substance[i] != 1) inclu.flag[i] <- 0

if(is.na(mydata.baseline$practice[i]) | mydata.baseline$practice[i]=="" | mydata.baseline$practice[i] == "B") inclu.flag[i] <- 0

if(is.na(mydata.baseline$suicide.base[i]) | mydata.baseline$suicide.base[i] > 0) inclu.flag[i] <- 0

if(is.na(mydata.baseline$compliance.physician[i]) | mydata.baseline$compliance.physician[i]==""| mydata.baseline$compliance.physician[i] %in% c("D")) inclu.flag[i] <- 0

}

###########################################################

#### Subpopulations and Generalized linear model

###########################################################

############## RCT

## RCT patients treated with R but without AE

RCT.patientID.R <- unique(mydata$patient.ID)[which(inclu.flag==1 & R.flag == 1 & AE.flag == 0)]

RCT.index.R <- which(unique(mydata$patient.ID) %in% RCT.patientID.R)

RCT.baseline.R.only <- mydata.baseline[RCT.index.R, ]

RCT.baseline.R.only$treatment <- R.flag[RCT.patientID.R]

RCT.3month.R.only <- mydata.3month[RCT.index.R, ]

RCT.3month.R.only$treatment <- R.flag[RCT.patientID.R]

############## SOHO

## SOHO patients treated with R but without AE

SOHO.patientID.R <- unique(mydata$patient.ID)[which(R.flag == 1 & AE.flag == 0)]

SOHO.index.R <- which(unique(mydata$patient.ID) %in% SOHO.patientID.R)

SOHO.baseline.R.only <- mydata.baseline[SOHO.index.R, ]

SOHO.baseline.R.only$treatment <- R.flag[SOHO.patientID.R]

SOHO.3month.R.only <- mydata.3month[SOHO.index.R, ]

SOHO.3month.R.only$treatment <- R.flag[SOHO.patientID.R]

###########################################################

#### Definition of 8 subpopulations

###########################################################

### subpopulation of augmentation: relax one eligibility criterion from RCT

inclu.flag.practice <- inclu.flag.suicide <- inclu.flag.alcohol <- inclu.flag.substance <- inclu.flag.duration <- inclu.flag.duration.suicide <- inclu.flag.practice.duration <- inclu.flag.alcohol.duration <- rep(1, nb.patient)

### SUBPOPULATION 1 : Duration

for(i in 1:nb.patient){

if(is.na(mydata.baseline$duration[i]) | (!is.na(mydata.baseline$duration[i]) && mydata.baseline$duration[i] < 1 | mydata.baseline$duration[i] >= 3)) inclu.flag.duration[i] <- 0

if(is.na(mydata.baseline$never.alcohol[i]) | mydata.baseline$never.alcohol[i] != 1) inclu.flag.duration[i] <- 0

if(is.na(mydata.baseline$never.substance[i]) | mydata.baseline$never.substance[i] != 1) inclu.flag.duration[i] <- 0

if(is.na(mydata.baseline$practice[i]) | mydata.baseline$practice[i]=="" | mydata.baseline$practice[i] == 2) inclu.flag.duration[i] <- 0

if(is.na(mydata.baseline$suicide.base[i]) | mydata.baseline$suicide.base[i] > 0) inclu.flag.duration[i] <- 0

if(is.na(mydata.baseline$compliance.physician[i]) | mydata.baseline$compliance.physician[i]==""| mydata.baseline$compliance.physician[i] == 4) inclu.flag.duration[i] <- 0

}

### SUBPOPULATION 2 : Alcohol abuse

for(i in 1:nb.patient){

if(is.na(mydata.baseline$duration[i]) | mydata.baseline$duration[i] < 3) inclu.flag.alcohol[i] <- 0

if(is.na(mydata.baseline$never.alcohol[i]) | (!is.na(mydata.baseline$never.alcohol[i]) && mydata.baseline$never.alcohol[i] == 1)) inclu.flag.alcohol[i] <- 0

if(is.na(mydata.baseline$never.substance[i]) | mydata.baseline$never.substance[i] != 1) inclu.flag.alcohol[i] <- 0

if(is.na(mydata.baseline$practice[i]) | mydata.baseline$practice[i]=="" | mydata.baseline$practice[i] == 2) inclu.flag.alcohol[i] <- 0

if(is.na(mydata.baseline$suicide.base[i]) | mydata.baseline$suicide.base[i] > 0) inclu.flag.alcohol[i] <- 0

if(is.na(mydata.baseline$compliance.physician[i]) | mydata.baseline$compliance.physician[i]==""| mydata.baseline$compliance.physician[i] == 4) inclu.flag.alcohol[i] <- 0

}

### SUBPOPULATION 3 : Substance abuse

for(i in 1:nb.patient){

if(is.na(mydata.baseline$duration[i]) | mydata.baseline$duration[i] < 3) inclu.flag.substance[i] <- 0

if(is.na(mydata.baseline$never.alcohol[i]) | mydata.baseline$never.alcohol[i] != 1) inclu.flag.substance[i] <- 0

if(is.na(mydata.baseline$never.substance[i]) | (!is.na(mydata.baseline$never.substance[i]) && mydata.baseline$never.substance[i] == 1)) inclu.flag.substance[i] <- 0

if(is.na(mydata.baseline$practice[i]) | mydata.baseline$practice[i]=="" | mydata.baseline$practice[i] == 2) inclu.flag.substance[i] <- 0

if(is.na(mydata.baseline$suicide.base[i]) | mydata.baseline$suicide.base[i] > 0) inclu.flag.substance[i] <- 0

if(is.na(mydata.baseline$compliance.physician[i]) | mydata.baseline$compliance.physician[i]==""| mydata.baseline$compliance.physician[i] == 4) inclu.flag.substance[i] <- 0

}

### SUBPOPULATION 4 : Practice

for(i in 1:nb.patient){

if(is.na(mydata.baseline$duration[i]) | mydata.baseline$duration[i] < 3) inclu.flag.practice[i] <- 0

if(is.na(mydata.baseline$never.alcohol[i]) | mydata.baseline$never.alcohol[i] != 1) inclu.flag.practice[i] <- 0

if(is.na(mydata.baseline$never.substance[i]) | mydata.baseline$never.substance[i] != 1) inclu.flag.practice[i] <- 0

if(is.na(mydata.baseline$practice[i]) | mydata.baseline$practice[i] =="" |(!is.na(mydata.baseline$practice[i]) && mydata.baseline$practice[i] !="" && mydata.baseline$practice[i] != 2)) inclu.flag.practice[i] <- 0

if(is.na(mydata.baseline$suicide.base[i]) | mydata.baseline$suicide.base[i] > 0) inclu.flag.practice[i] <- 0

if(is.na(mydata.baseline$compliance.physician[i]) | mydata.baseline$compliance.physician[i]==""| mydata.baseline$compliance.physician[i] == 4) inclu.flag.practice[i] <- 0

}

### SUBPOPULATION 5 : Suicide attempt

for(i in 1:nb.patient){

if(is.na(mydata.baseline$duration[i]) | mydata.baseline$duration[i] < 3) inclu.flag.suicide[i] <- 0

if(is.na(mydata.baseline$never.alcohol[i]) | mydata.baseline$never.alcohol[i] != 1) inclu.flag.suicide[i] <- 0

if(is.na(mydata.baseline$never.substance[i]) | mydata.baseline$never.substance[i] != 1) inclu.flag.suicide[i] <- 0

if(is.na(mydata.baseline$practice[i]) | mydata.baseline$practice[i]=="" | mydata.baseline$practice[i] == 2) inclu.flag.suicide[i] <- 0

if(is.na(mydata.baseline$suicide.base[i]) | (!is.na(mydata.baseline$suicide.base[i]) && mydata.baseline$suicide.base[i] <= 0 | mydata.baseline$suicide.base[i] >1)) inclu.flag.suicide[i] <- 0

if(is.na(mydata.baseline$compliance.physician[i]) | mydata.baseline$compliance.physician[i]==""| mydata.baseline$compliance.physician[i] == 4) inclu.flag.suicide[i] <- 0

}

### SUBPOPULATION 6 : Duration of illness + Suicide attempt

for(i in 1:nb.patient){

if(is.na(mydata.baseline$duration[i]) | (!is.na(mydata.baseline$duration[i]) && mydata.baseline$duration[i] < 1 | mydata.baseline$duration[i] >= 3)) inclu.flag.duration.suicide[i] <- 0

if(is.na(mydata.baseline$never.alcohol[i]) | mydata.baseline$never.alcohol[i] != 1) inclu.flag.duration.suicide[i] <- 0

if(is.na(mydata.baseline$never.substance[i]) | mydata.baseline$never.substance[i] != 1) inclu.flag.duration.suicide[i] <- 0

if(is.na(mydata.baseline$practice[i]) | mydata.baseline$practice[i]=="" | mydata.baseline$practice[i] == 2) inclu.flag.duration.suicide[i] <- 0

if(is.na(mydata.baseline$suicide.base[i]) | (!is.na(mydata.baseline$suicide.base[i]) && mydata.baseline$suicide.base[i] <= 0 | mydata.baseline$suicide.base[i] >1)) inclu.flag.duration.suicide[i] <- 0

if(is.na(mydata.baseline$compliance.physician[i]) | mydata.baseline$compliance.physician[i]==""| mydata.baseline$compliance.physician[i] == 4) inclu.flag.duration.suicide[i] <- 0

}

### SUBPOPULATION 7 : Duration of illness + alcohol abuse

for(i in 1:nb.patient){

if(is.na(mydata.baseline$duration[i]) | (!is.na(mydata.baseline$duration[i]) && mydata.baseline$duration[i] < 1 | mydata.baseline$duration[i] >= 3)) inclu.flag.alcohol.duration[i] <- 0

if(is.na(mydata.baseline$never.alcohol[i]) | (!is.na(mydata.baseline$never.alcohol[i]) && mydata.baseline$never.alcohol[i] == 1)) inclu.flag.alcohol.duration[i] <- 0

if(is.na(mydata.baseline$never.substance[i]) | mydata.baseline$never.substance[i] != 1) inclu.flag.alcohol.duration[i] <- 0

if(is.na(mydata.baseline$practice[i]) | mydata.baseline$practice[i]=="" | mydata.baseline$practice[i] == 2) inclu.flag.alcohol.duration[i] <- 0

if(is.na(mydata.baseline$suicide.base[i]) | mydata.baseline$suicide.base[i] > 0) inclu.flag.alcohol.duration[i] <- 0

if(is.na(mydata.baseline$compliance.physician[i]) | mydata.baseline$compliance.physician[i]==""| mydata.baseline$compliance.physician[i] == 4) inclu.flag.alcohol.duration[i] <- 0

}

### SUBPOPULATION 8 : Duration of illness + practice

for(i in 1:nb.patient){

if(is.na(mydata.baseline$duration[i]) | (!is.na(mydata.baseline$duration[i]) && mydata.baseline$duration[i] < 1 | mydata.baseline$duration[i] >= 3)) inclu.flag.practice.duration[i] <- 0

if(is.na(mydata.baseline$never.alcohol[i]) | mydata.baseline$never.alcohol[i] != 1) inclu.flag.practice.duration[i] <- 0

if(is.na(mydata.baseline$never.substance[i]) | mydata.baseline$never.substance[i] != 1) inclu.flag.practice.duration[i] <- 0

if(is.na(mydata.baseline$practice[i]) | mydata.baseline$practice[i]=="" |(!is.na(mydata.baseline$practice[i]) && mydata.baseline$practice[i] !="" && mydata.baseline$practice[i] != 2)) inclu.flag.practice.duration[i] <- 0

if(is.na(mydata.baseline$suicide.base[i]) | mydata.baseline$suicide.base[i] > 0) inclu.flag.practice.duration[i] <- 0

if(is.na(mydata.baseline$compliance.physician[i]) | mydata.baseline$compliance.physician[i]==""| mydata.baseline$compliance.physician[i] == 4) inclu.flag.practice.duration[i] <- 0

}

## Subpopulation creation: patients under R but not AE

# RCT population

RCT.R <- mydata.baseline[inclu.flag==1 & R.flag == 1 & AE.flag == 0,]

RCT.R$CGIS.baseline <- RCT.R$overall.symptom

RCT.R$CGIS.3month <- mydata.3month[inclu.flag==1 & R.flag == 1 & AE.flag == 0,]$overall.symptom

# Illness duration subpopulation

SUBPOP.X.DURATION.R <- mydata.baseline[inclu.flag.duration==1 & R.flag == 1 & AE.flag == 0,]

SUBPOP.X.DURATION.R$CGIS.baseline <- SUBPOP.X.DURATION.R$overall.symptom

SUBPOP.X.DURATION.R$CGIS.3month <- mydata.3month[inclu.flag.duration==1 & R.flag == 1 & AE.flag == 0,]$overall.symptom

# Practice type subpopulation

SUBPOP.X.PRACTICE.R <- mydata.baseline[inclu.flag.practice==1 & R.flag == 1 & AE.flag == 0,]

SUBPOP.X.PRACTICE.R$CGIS.baseline <- SUBPOP.X.PRACTICE.R$overall.symptom

SUBPOP.X.PRACTICE.R$CGIS.3month <- mydata.3month[inclu.flag.practice==1 & R.flag == 1 & AE.flag == 0,]$overall.symptom

# Suicide attempts subpopulation

SUBPOP.X.SUICIDE.R <- mydata.baseline[inclu.flag.suicide==1 & R.flag == 1 & AE.flag == 0,]

SUBPOP.X.SUICIDE.R$CGIS.baseline <- SUBPOP.X.SUICIDE.R$overall.symptom

SUBPOP.X.SUICIDE.R$CGIS.3month <- mydata.3month[inclu.flag.suicide==1 & R.flag == 1 & AE.flag == 0,]$overall.symptom

# Alcohol abuse subpopulation

SUBPOP.X.ALCOHOL.R <- mydata.baseline[inclu.flag.alcohol==1 & R.flag == 1 & AE.flag == 0,]

SUBPOP.X.ALCOHOL.R$CGIS.baseline <- SUBPOP.X.ALCOHOL.R$overall.symptom

SUBPOP.X.ALCOHOL.R$CGIS.3month <- mydata.3month[inclu.flag.alcohol==1 & R.flag == 1 & AE.flag == 0,]$overall.symptom

# Drug abuse subpopulation

SUBPOP.X.SUBSTANCE.R <- mydata.baseline[inclu.flag.substance==1 & R.flag == 1 & AE.flag == 0,]

SUBPOP.X.SUBSTANCE.R$CGIS.baseline <- SUBPOP.X.SUBSTANCE.R$overall.symptom

SUBPOP.X.SUBSTANCE.R$CGIS.3month <- mydata.3month[inclu.flag.substance==1 & R.flag == 1 & AE.flag == 0,]$overall.symptom

# Illness duration+suicide attempt subpopulation

SUBPOP.X.SUICIDE.DURATION.R <- mydata.baseline[(inclu.flag.duration.suicide==1 | inclu.flag.duration==1 | inclu.flag.suicide==1) & R.flag == 1 & AE.flag == 0,]; dim(SUBPOP.X.SUICIDE.DURATION.R)

SUBPOP.X.SUICIDE.DURATION.R$CGIS.baseline <- SUBPOP.X.SUICIDE.DURATION.R$overall.symptom

SUBPOP.X.SUICIDE.DURATION.R$CGIS.3month <- mydata.3month[(inclu.flag.duration.suicide==1 | inclu.flag.duration==1 | inclu.flag.suicide==1) & R.flag == 1 & AE.flag == 0,]$overall.symptom

# Illness duration + Alcohol subpopulation

SUBPOP.X.DURATION.ALCOHOL.R <- mydata.baseline[(inclu.flag.alcohol.duration==1 | inclu.flag.alcohol==1 | inclu.flag.duration==1) & R.flag == 1 & AE.flag == 0,]; dim(SUBPOP.X.DURATION.ALCOHOL.R)

SUBPOP.X.DURATION.ALCOHOL.R$CGIS.baseline <- SUBPOP.X.DURATION.ALCOHOL.R$overall.symptom

SUBPOP.X.DURATION.ALCOHOL.R$CGIS.3month <- mydata.3month[(inclu.flag.alcohol.duration==1 | inclu.flag.alcohol==1 | inclu.flag.duration==1) & R.flag == 1 & AE.flag == 0,]$overall.symptom

# Illness duration + Practice subpopulation

SUBPOP.X.DURATION.PRACTICE.R <- mydata.baseline[(inclu.flag.practice.duration==1 | inclu.flag.practice==1 | inclu.flag.duration==1) & R.flag == 1 & AE.flag == 0,]; dim(SUBPOP.X.DURATION.PRACTICE.R)

SUBPOP.X.DURATION.PRACTICE.R$CGIS.baseline <- SUBPOP.X.DURATION.PRACTICE.R$overall.symptom

SUBPOP.X.DURATION.PRACTICE.R$CGIS.3month <- mydata.3month[(inclu.flag.practice.duration==1 | inclu.flag.practice==1 | inclu.flag.duration==1) & R.flag == 1 & AE.flag == 0,]$overall.symptom

# SOHO population

SOHO.R <- mydata.baseline[R.flag == 1 & AE.flag == 0,]

SOHO.R$CGIS.baseline <- SOHO.R$overall.symptom

SOHO.R$CGIS.3month <- mydata.3month[R.flag == 1 & AE.flag == 0,]$overall.symptom

### Code used to predict real-world effectiveness

#############################################

### PREDICTION MODEL

#############################################

set.seed(42)

library(MASS)

# Preparation of necessary covariates

RCT.R$CGIS.change.3month <- RCT.R$CGIS.3month - RCT.R$CGIS.baseline

RCT.R$hospitalization <- RCT.R$nb.days

RCT.R$hospitalization[which(RCT.R$hospitalization > 0)] = 1

SOHO.R$CGIS.change.3month <- SOHO.R$CGIS.3month-SOHO.R$CGIS.baseline

SOHO.R$hospitalization <- SOHO.R$nb.days

SOHO.R$hospitalization[which(SOHO.R$hospitalization > 0)] = 1

############################################################################

### Augmentation of the reference RCT and prediction

############################################################################

size.RCT.target <- dim(RCT.R)[1]

nb.repeat <- 500

#################################################################

### SUBPOPULATION 1: Duration of illness

SUBPOP.X.DURATION.R$durationgroup <- cut(as.numeric(SUBPOP.X.DURATION.R$duration), breaks=c(0,1,3, seq(15, 55, 10),65), labels=c("0-1","1-3","3-15", "15-25", "25-35", "35-45", "45-55", "55-65"))

RCT.R$durationgroup <- cut(as.numeric(RCT.R$duration), breaks=c(0,1,3, seq(15, 55, 10),65), labels=c("0-1","1-3","3-15", "15-25", "25-35", "35-45", "45-55", "55-65"))

SOHO.R$durationgroup <- cut(as.numeric(SOHO.R$duration), breaks=c(0,1,3, seq(15, 55, 10),65), labels=c("0-1","1-3","3-15", "15-25", "25-35", "35-45", "45-55", "55-65"))

RCT.R.durationgroup.table <- c( length(which(is.na(RCT.R$duration))),table(RCT.R$durationgroup))

names(RCT.R.durationgroup.table)[1]="NA"

SUBPOP.X.DURATION.R.durationgroup.table <- c( length(which(is.na(SUBPOP.X.DURATION.R$duration))),table(SUBPOP.X.DURATION.R$durationgroup))

names(SUBPOP.X.DURATION.R.durationgroup.table)[1]="NA"

SOHO.R.durationgroup.table <- c( length(which(is.na(SOHO.R$duration))),table(SOHO.R$durationgroup))

names(SOHO.R.durationgroup.table)[1]="NA"

### Augmentation procedure

sub.duration.size <- dim(SUBPOP.X.DURATION.R)[1]

enrich.size <- seq(0,sub.duration.size,max(round(sub.duration.size/100),1))

predict.enrichset.R.duration <- array(0, dim=c(length(SOHO.R$CGIS.change.3month), length(enrich.size), nb.repeat))

mean.sd.enrichset.R.duration <- array(0, dim=c(2, length(enrich.size), nb.repeat))

enrichset.ID.R.duration <- as.list(numeric(length(enrich.size)*nb.repeat)); dim(enrichset.ID.R.duration) <- c(length(enrich.size), nb.repeat)

for(l in 1:nb.repeat){

if(l%%100 == 0) print(l)

for(i in 1:length(enrich.size)){

### Prepare the patient ID in X and in the reference RCT

enrich.ID <- sample(SUBPOP.X.DURATION.R$patient.ID, enrich.size[i])

RCT.ID <- sample(RCT.R$patient.ID, size.RCT.target-enrich.size[i])

enrichset.ID.R.duration[[i,l]] <- enrichset.R.duration.ID <- factor(c(as.character(RCT.ID),as.character(enrich.ID)))

### The data frame of the augmented RCT = enrichset.R.duration

enrichset.R.duration <- SOHO.R[which(SOHO.R$patient.ID %in% enrichset.R.duration.ID),]

### The mean and sd of the CGIS change (from baseline to 3 months) in the augmented RCTs

mean.sd.enrichset.R.duration[1,i,l] <- mean(enrichset.R.duration$CGIS.3month,na.rm=T)

mean.sd.enrichset.R.duration[2,i,l] <- sd(enrichset.R.duration$CGIS.3month,na.rm=T)

## MODEL FITTING

enrichset.R.duration$CGIS.3month2 =as.factor(enrichset.R.duration$CGIS.3month)

opr.enrichset.R.duration <- polr(CGIS.3month2 ~ age + duration + BMI + compliance.physician + practice + gender + hospitalization + qualitylife.anxiety + qualitylife.vas + qualitylife.mobility + qualitylife.pain + qualitylife.selfcare + qualitylife.activity + country + work.base + house.base + social.base + spouse.base + negative.symptom + positive.symptom + cognitive.symptom + nb.admit + dosage.R.after + CGIS.baseline, data=enrichset.R.duration, method = "logistic", na.action=na.omit)

######## New data for FULL MODEL

SOHO.R.dat <- data.frame(1,age=SOHO.R$age, duration=SOHO.R$duration, BMI=SOHO.R$BMI, compliance.physician=SOHO.R$compliance.physician, practice=SOHO.R$practice, gender=SOHO.R$gender, hospitalization=SOHO.R$hospitalization, qualitylife.anxiety=SOHO.R$qualitylife.anxiety, qualitylife.vas=SOHO.R$qualitylife.vas,qualitylife.mobility=SOHO.R$qualitylife.mobility, qualitylife.pain=SOHO.R$qualitylife.pain, qualitylife.selfcare=SOHO.R$qualitylife.selfcare, qualitylife.activity=SOHO.R$qualitylife.activity, country=SOHO.R$country, work.base=SOHO.R$work.base, house.base=SOHO.R$house.base, social.base=SOHO.R$social.base, spouse.base=SOHO.R$spouse.base, negative.symptom=SOHO.R$negative.symptom, positive.symptom=SOHO.R$positive.symptom, cognitive.symptom=SOHO.R$cognitive.symptom, nb.admit=SOHO.R$nb.admit, dosage.R.after=SOHO.R$dosage.R.after, CGIS.baseline=SOHO.R$CGIS.baseline)

### Prediction in SOHO using the regression model

estimate.coef.duration.opr <- as.numeric(suppressMessages(summary(opr.enrichset.R.duration))$coefficients[1:24,1])

estimate.Z.duration.opr<- as.matrix(SOHO.R.dat[,-1])%*% estimate.coef.duration.opr

predict.enrichset.R.duration[,i,l] <- ZtoY(estimate.Z.duration.opr, alpha=suppressMessages(summary(opr.enrichset.R.duration))$coefficients[25:30,1])

}

}

### Check the prediction MSE in each augmented RCT subpopulation

nb.subset.duration <- length(enrich.size)

mse.enrichset.duration <- sd.mse.enrichset.duration <- rep(0, nb.subset.duration)

mse.per.repeat.enrichset.duration <- matrix(0, nb.subset.duration, nb.repeat)

ci.mse.enrichset.duration <- matrix(0, nb.subset.duration, 2)

for(i in 1:nb.subset.duration)

{

if(dim(predict.enrichset.R.duration)[3] == 1){

mse.enrichset.duration[i] = mean((predict.enrichset.R.duration[,i,1] - SOHO.R$CGIS.3month)^2,na.rm=T)

sd.mse.enrichset.duration[i] = sd((predict.enrichset.R.duration[,i,1] - SOHO.R$CGIS.3month)^2,na.rm=T)

ci.mse.enrichset.duration[i,] = c(mse.enrichset.duration[i]-1.96*sd.mse.enrichset.duration[i],mse.enrichset.duration[i]+1.96*sd.mse.enrichset.duration[i])

} else {

mse.enrichset.duration[i] = mean((rowMeans(predict.enrichset.R.duration[,i,]) - SOHO.R$CGIS.3month)^2,na.rm=T)

mse.per.repeat.enrichset.duration[i,1] = mean((predict.enrichset.R.duration[,i,1] - SOHO.R$CGIS.3month)^2,na.rm=T)

for(j in 2:nb.repeat){

mse.per.repeat.enrichset.duration[i,j] = mean((rowMeans(predict.enrichset.R.duration[,i,1:j]) - SOHO.R$CGIS.3month)^2,na.rm=T)

}

sd.mse.enrichset.duration[i] = sd(mse.per.repeat.enrichset.duration[i,],na.rm=T)

ci.mse.enrichset.duration[i,] = c(mse.enrichset.duration[i]-1.96*sd.mse.enrichset.duration[i],mse.enrichset.duration[i]+1.96*sd.mse.enrichset.duration[i])

}

}

### Check the prediction bias in each augmented RCT subpopulation

bias.enrichset.duration <- sd.bias.enrichset.duration <- rep(0, nb.subset.duration)

bias.per.repeat.enrichset.duration <- matrix(0, nb.subset.duration, nb.repeat)

ci.bias.enrichset.duration <- matrix(0, nb.subset.duration, 2)

for(i in 1:nb.subset.duration)

{

if(dim(predict.enrichset.R.duration)[3] == 1){

bias.enrichset.duration[i] = mean((predict.enrichset.R.duration[,i,1] - SOHO.R$CGIS.3month),na.rm=T)

sd.bias.enrichset.duration[i] = sd((predict.enrichset.R.duration[,i,1] - SOHO.R$CGIS.3month),na.rm=T)

ci.bias.enrichset.duration[i,] = c(bias.enrichset.duration[i]-1.96*sd.bias.enrichset.duration[i],bias.enrichset.duration[i]+1.96*sd.bias.enrichset.duration[i])

} else {

bias.enrichset.duration[i] = mean((rowMeans(predict.enrichset.R.duration[,i,]) - SOHO.R$CGIS.3month),na.rm=T)

bias.per.repeat.enrichset.duration[i,1] = mean((predict.enrichset.R.duration[,i,1] - SOHO.R$CGIS.3month),na.rm=T)

for(j in 2:nb.repeat){

bias.per.repeat.enrichset.duration[i,j] = mean((rowMeans(predict.enrichset.R.duration[,i,1:j]) - SOHO.R$CGIS.3month),na.rm=T)

}

sd.bias.enrichset.duration[i] = sd(bias.per.repeat.enrichset.duration[i,],na.rm=T)

ci.bias.enrichset.duration[i,] = c(bias.enrichset.duration[i]-1.96*sd.bias.enrichset.duration[i],bias.enrichset.duration[i]+1.96*sd.bias.enrichset.duration[i])

}

}

#################################################################################

#### SUBPOPULATION 2: Practice type

RCT.R.practice.table <- SUBPOP.X.PRACTICE.R.practice.table <- SOHO.R.practice.table <- rep(0,4)

RCT.R.practice.table[c(2,4)] <- table(RCT.R$practice)

RCT.R.practice.table[1] <- length(which(is.na(RCT.R$practice)))

names(RCT.R.practice.table)[1]="NA"; names(RCT.R.practice.table)[2]="Public"; names(RCT.R.practice.table)[3]="Private"; names(RCT.R.practice.table)[4]="Combined";

SUBPOP.X.PRACTICE.R.practice.table[3] <- table(SUBPOP.X.PRACTICE.R$practice)

SUBPOP.X.PRACTICE.R.practice.table[1] <- length(which(is.na(SUBPOP.X.PRACTICE.R$practice)))

names(SUBPOP.X.PRACTICE.R.practice.table)[1]="NA"; names(SUBPOP.X.PRACTICE.R.practice.table)[2]="Public"; names(SUBPOP.X.PRACTICE.R.practice.table)[3]="Private"; names(SUBPOP.X.PRACTICE.R.practice.table)[4]="Combined";

SOHO.R.practice.table[2:4] <- table(SOHO.R$practice)

SOHO.R.practice.table[1] <- length(which(is.na(SOHO.R$practice)))

names(SOHO.R.practice.table)[1]="NA"; names(SOHO.R.practice.table)[2]="Public"; names(SOHO.R.practice.table)[3]="Private"; names(SOHO.R.practice.table)[4]="Combined";

### Augmentation procedure

sub.practice.size <- dim(SUBPOP.X.PRACTICE.R)[1]

enrich.size <- seq(0,sub.practice.size,max(round(sub.practice.size/100),1))

predict.enrichset.R.practice <- array(0, dim=c(length(SOHO.R$CGIS.change.3month), length(enrich.size), nb.repeat))

mean.sd.enrichset.R.practice <- array(0, dim=c(2, length(enrich.size), nb.repeat))

enrichset.ID.R.practice <- as.list(numeric(length(enrich.size)*nb.repeat)); dim(enrichset.ID.R.practice) <- c(length(enrich.size), nb.repeat)

for(l in 1:nb.repeat){

if(l%%100 == 0) print(l)

for(i in 1:length(enrich.size)){

### Prepare the patient ID in X and in the reference RCT

enrich.ID <- sample(SUBPOP.X.PRACTICE.R$patient.ID, enrich.size[i])

RCT.ID <- sample(RCT.R$patient.ID, size.RCT.target-enrich.size[i])

enrichset.ID.R.practice[[i,l]] <- enrichset.R.practice.ID <- factor(c(as.character(RCT.ID),as.character(enrich.ID)))

### The data frame of the augmented RCT = enrichset.R.practice

enrichset.R.practice <- SOHO.R[which(SOHO.R$patient.ID %in% enrichset.R.practice.ID),]

### The mean and sd of the CGIS change (from baseline to 3 months) in the augmented RCTs

mean.sd.enrichset.R.practice[1,i,l] <- mean(enrichset.R.practice$CGIS.3month,na.rm=T)

mean.sd.enrichset.R.practice[2,i,l] <- sd(enrichset.R.practice$CGIS.3month,na.rm=T)

## MODEL FITTING

enrichset.R.practice$CGIS.3month2 =as.factor(enrichset.R.practice$CGIS.3month)

opr.enrichset.R.practice <- polr(CGIS.3month2 ~ age + duration + BMI + compliance.physician + practice + gender + hospitalization + qualitylife.anxiety + qualitylife.vas + qualitylife.mobility + qualitylife.pain + qualitylife.selfcare + qualitylife.activity + country + work.base + house.base + social.base + spouse.base + negative.symptom + positive.symptom + cognitive.symptom + nb.admit + dosage.R.after + CGIS.baseline, data=enrichset.R.practice, method = "logistic", na.action=na.omit)

### Preparation of the new data = SOHO population

SOHO.R.dat <- data.frame(1,age=SOHO.R$age, duration=SOHO.R$duration, BMI=SOHO.R$BMI, compliance.physician=SOHO.R$compliance.physician, practice=SOHO.R$practice, gender=SOHO.R$gender, hospitalization=SOHO.R$hospitalization, qualitylife.anxiety=SOHO.R$qualitylife.anxiety, qualitylife.vas=SOHO.R$qualitylife.vas,qualitylife.mobility=SOHO.R$qualitylife.mobility, qualitylife.pain=SOHO.R$qualitylife.pain, qualitylife.selfcare=SOHO.R$qualitylife.selfcare, qualitylife.activity=SOHO.R$qualitylife.activity, country=SOHO.R$country, work.base=SOHO.R$work.base, house.base=SOHO.R$house.base, social.base=SOHO.R$social.base, spouse.base=SOHO.R$spouse.base, negative.symptom=SOHO.R$negative.symptom, positive.symptom=SOHO.R$positive.symptom, cognitive.symptom=SOHO.R$cognitive.symptom, nb.admit=SOHO.R$nb.admit, dosage.R.after=SOHO.R$dosage.R.after, CGIS.baseline=SOHO.R$CGIS.baseline)

### Prediction in SOHO using the regression model

estimate.coef.practice.opr <- as.numeric(suppressMessages(summary(opr.enrichset.R.practice))$coefficients[1:24,1])

estimate.Z.practice.opr<- as.matrix(SOHO.R.dat[,-1])%*% estimate.coef.practice.opr

predict.enrichset.R.practice[,i,l] <- ZtoY(estimate.Z.practice.opr, alpha=suppressMessages(summary(opr.enrichset.R.practice))$coefficients[25:30,1])

}

}

### Check the prediction MSE in each augmented RCT subpopulation

nb.subset.practice <- length(enrich.size)

mse.enrichset.practice <- sd.mse.enrichset.practice <- rep(0, nb.subset.practice)

mse.per.repeat.enrichset.practice <- matrix(0, nb.subset.practice, nb.repeat)

ci.mse.enrichset.practice <- matrix(0, nb.subset.practice, 2)

for(i in 1:nb.subset.practice)

{

if(dim(predict.enrichset.R.practice)[3] == 1){

mse.enrichset.practice[i] = mean((predict.enrichset.R.practice[,i,1] - SOHO.R$CGIS.3month)^2,na.rm=T)

sd.mse.enrichset.practice[i] = sd((predict.enrichset.R.practice[,i,1] - SOHO.R$CGIS.3month)^2,na.rm=T)

ci.mse.enrichset.practice[i,] = c(mse.enrichset.practice[i]-1.96*sd.mse.enrichset.practice[i],mse.enrichset.practice[i]+1.96*sd.mse.enrichset.practice[i])

} else {

mse.enrichset.practice[i] = mean((rowMeans(predict.enrichset.R.practice[,i,]) - SOHO.R$CGIS.3month)^2,na.rm=T)

mse.per.repeat.enrichset.practice[i,1] = mean((predict.enrichset.R.practice[,i,1] - SOHO.R$CGIS.3month)^2,na.rm=T)

for(j in 2:nb.repeat){

mse.per.repeat.enrichset.practice[i,j] = mean((rowMeans(predict.enrichset.R.practice[,i,1:j]) - SOHO.R$CGIS.3month)^2,na.rm=T)

}

sd.mse.enrichset.practice[i] = sd(mse.per.repeat.enrichset.practice[i,],na.rm=T)

ci.mse.enrichset.practice[i,] = c(mse.enrichset.practice[i]-1.96*sd.mse.enrichset.practice[i],mse.enrichset.practice[i]+1.96*sd.mse.enrichset.practice[i])

}

}

### Check the prediction bias in each augmented RCT subpopulation

bias.enrichset.practice <- sd.bias.enrichset.practice <- rep(0, nb.subset.practice)

bias.per.repeat.enrichset.practice <- matrix(0, nb.subset.practice, nb.repeat)

ci.bias.enrichset.practice <- matrix(0, nb.subset.practice, 2)

for(i in 1:nb.subset.practice)

{

if(dim(predict.enrichset.R.practice)[3] == 1){

bias.enrichset.practice[i] = mean((predict.enrichset.R.practice[,i,1] - SOHO.R$CGIS.3month),na.rm=T)

sd.bias.enrichset.practice[i] = sd((predict.enrichset.R.practice[,i,1] - SOHO.R$CGIS.3month),na.rm=T)

ci.bias.enrichset.practice[i,] = c(bias.enrichset.practice[i]-1.96*sd.bias.enrichset.practice[i],bias.enrichset.practice[i]+1.96*sd.bias.enrichset.practice[i])

} else {

bias.enrichset.practice[i] = mean((rowMeans(predict.enrichset.R.practice[,i,]) - SOHO.R$CGIS.3month),na.rm=T)

bias.per.repeat.enrichset.practice[i,1] = mean((predict.enrichset.R.practice[,i,1] - SOHO.R$CGIS.3month),na.rm=T)

for(j in 2:nb.repeat){

bias.per.repeat.enrichset.practice[i,j] = mean((rowMeans(predict.enrichset.R.practice[,i,1:j]) - SOHO.R$CGIS.3month),na.rm=T)

}

sd.bias.enrichset.practice[i] = sd(bias.per.repeat.enrichset.practice[i,],na.rm=T)

ci.bias.enrichset.practice[i,] = c(bias.enrichset.practice[i]-1.96*sd.bias.enrichset.practice[i],bias.enrichset.practice[i]+1.96*sd.bias.enrichset.practice[i])

}

}

##################################################################################

### SUBPOPULATION 3: Suicide attempt

SUBPOP.X.SUICIDE.R$suicidegroup <- cut(as.numeric(SUBPOP.X.SUICIDE.R$suicide.base), breaks=c(-0.5, 0.5, 1.5, 5.5, 60), labels=c("0", "1", "1-5","6+"))

RCT.R$suicidegroup <- cut(as.numeric(RCT.R$suicide.base), breaks=c(-0.5, 0.5, 1.5, 5.5, 60), labels=c("0", "1", "1-5","6+"))

SOHO.R$suicidegroup <- cut(as.numeric(SOHO.R$suicide.base), breaks=c(-0.5, 0.5, 1.5, 5.5, 60), labels=c("0", "1", "1-5","6+"))

RCT.R.suicidegroup.table <- c( length(which(is.na(RCT.R$suicide.base))),table(RCT.R$suicidegroup))

names(RCT.R.suicidegroup.table)[1]="NA"

SUBPOP.X.SUICIDE.R.suicidegroup.table <- c( length(which(is.na(SUBPOP.X.SUICIDE.R$suicide.base))),table(SUBPOP.X.SUICIDE.R$suicidegroup))

names(SUBPOP.X.SUICIDE.R.suicidegroup.table)[1]="NA"

SOHO.R.suicidegroup.table <- c( length(which(is.na(SOHO.R$suicide.base))),table(SOHO.R$suicidegroup))

names(SOHO.R.suicidegroup.table)[1]="NA"

### Augmentation procedure

sub.suicide.size <- dim(SUBPOP.X.SUICIDE.R)[1]

enrich.size <- seq(0,sub.suicide.size,max(round(sub.suicide.size/100),1))

predict.enrichset.R.suicide <- array(0, dim=c(length(SOHO.R$CGIS.change.3month), length(enrich.size), nb.repeat))

mean.sd.enrichset.R.suicide <- array(0, dim=c(2, length(enrich.size), nb.repeat))

enrichset.ID.R.suicide <- as.list(numeric(length(enrich.size)*nb.repeat)); dim(enrichset.ID.R.suicide) <- c(length(enrich.size), nb.repeat)

for(l in 1:nb.repeat){

if(l%%100 == 0) print(l)

for(i in 1:length(enrich.size)){

### Prepare the patient ID in X and in the reference RCT

enrich.ID <- sample(SUBPOP.X.SUICIDE.R$patient.ID, enrich.size[i])

RCT.ID <- sample(RCT.R$patient.ID, size.RCT.target-enrich.size[i])

enrichset.ID.R.suicide[[i,l]] <- enrichset.R.suicide.ID <- factor(c(as.character(RCT.ID),as.character(enrich.ID)))

### The data frame of the augmented RCT = enrichset.R.suicide

enrichset.R.suicide <- SOHO.R[which(SOHO.R$patient.ID %in% enrichset.R.suicide.ID),]

### The mean and sd of the CGIS change (from baseline to 3 months) in the augmented RCTs

mean.sd.enrichset.R.suicide[1,i,l] <- mean(enrichset.R.suicide$CGIS.3month,na.rm=T)

mean.sd.enrichset.R.suicide[2,i,l] <- sd(enrichset.R.suicide$CGIS.3month,na.rm=T)

## MODEL FITTING

enrichset.R.suicide$CGIS.3month2 =as.factor(enrichset.R.suicide$CGIS.3month)

opr.enrichset.R.suicide <- polr(CGIS.3month2 ~ age + duration + BMI + compliance.physician + practice + gender + hospitalization + qualitylife.anxiety + qualitylife.vas + qualitylife.mobility + qualitylife.pain + qualitylife.selfcare + qualitylife.activity + country + work.base + house.base + social.base + spouse.base + negative.symptom + positive.symptom + cognitive.symptom + nb.admit + dosage.R.after + CGIS.baseline, data=enrichset.R.suicide, method = "logistic", na.action=na.omit)

### Preparation of the new data = SOHO population

SOHO.R.dat <- data.frame(1,age=SOHO.R$age, duration=SOHO.R$duration, BMI=SOHO.R$BMI, compliance.physician=SOHO.R$compliance.physician, practice=SOHO.R$practice, gender=SOHO.R$gender, hospitalization=SOHO.R$hospitalization, qualitylife.anxiety=SOHO.R$qualitylife.anxiety, qualitylife.vas=SOHO.R$qualitylife.vas,qualitylife.mobility=SOHO.R$qualitylife.mobility, qualitylife.pain=SOHO.R$qualitylife.pain, qualitylife.selfcare=SOHO.R$qualitylife.selfcare, qualitylife.activity=SOHO.R$qualitylife.activity, country=SOHO.R$country, work.base=SOHO.R$work.base, house.base=SOHO.R$house.base, social.base=SOHO.R$social.base, spouse.base=SOHO.R$spouse.base, negative.symptom=SOHO.R$negative.symptom, positive.symptom=SOHO.R$positive.symptom, cognitive.symptom=SOHO.R$cognitive.symptom, nb.admit=SOHO.R$nb.admit, dosage.R.after=SOHO.R$dosage.R.after, CGIS.baseline=SOHO.R$CGIS.baseline)

### Prediction in SOHO using the regression model

estimate.coef.suicide.opr <- as.numeric(suppressMessages(summary(opr.enrichset.R.suicide))$coefficients[1:24,1])

estimate.Z.suicide.opr<- as.matrix(SOHO.R.dat[,-1])%*% estimate.coef.suicide.opr

predict.enrichset.R.suicide[,i,l] <- ZtoY(estimate.Z.suicide.opr, alpha=suppressMessages(summary(opr.enrichset.R.suicide))$coefficients[25:30,1])

}

}

### Check the prediction MSE in each augmented RCT subpopulation

nb.subset.suicide <- length(enrich.size)

mse.enrichset.suicide <- sd.mse.enrichset.suicide <- rep(0, nb.subset.suicide)

mse.per.repeat.enrichset.suicide <- matrix(0, nb.subset.suicide, nb.repeat)

ci.mse.enrichset.suicide <- matrix(0, nb.subset.suicide, 2)

for(i in 1:nb.subset.suicide)

{

if(dim(predict.enrichset.R.suicide)[3] == 1){

mse.enrichset.suicide[i] = mean((predict.enrichset.R.suicide[,i,1] - SOHO.R$CGIS.3month)^2,na.rm=T)

sd.mse.enrichset.suicide[i] = sd((predict.enrichset.R.suicide[,i,1] - SOHO.R$CGIS.3month)^2,na.rm=T)

ci.mse.enrichset.suicide[i,] = c(mse.enrichset.suicide[i]-1.96*sd.mse.enrichset.suicide[i],mse.enrichset.suicide[i]+1.96*sd.mse.enrichset.suicide[i])

} else {

mse.enrichset.suicide[i] = mean((rowMeans(predict.enrichset.R.suicide[,i,]) - SOHO.R$CGIS.3month)^2,na.rm=T)

mse.per.repeat.enrichset.suicide[i,1] = mean((predict.enrichset.R.suicide[,i,1] - SOHO.R$CGIS.3month)^2,na.rm=T)

for(j in 2:nb.repeat){

mse.per.repeat.enrichset.suicide[i,j] = mean((rowMeans(predict.enrichset.R.suicide[,i,1:j]) - SOHO.R$CGIS.3month)^2,na.rm=T)

}

sd.mse.enrichset.suicide[i] = sd(mse.per.repeat.enrichset.suicide[i,],na.rm=T)

ci.mse.enrichset.suicide[i,] = c(mse.enrichset.suicide[i]-1.96*sd.mse.enrichset.suicide[i],mse.enrichset.suicide[i]+1.96*sd.mse.enrichset.suicide[i])

}

}

### Check the prediction bias in each augmented RCT subpopulation

bias.enrichset.suicide <- sd.bias.enrichset.suicide <- rep(0, nb.subset.suicide)

bias.per.repeat.enrichset.suicide <- matrix(0, nb.subset.suicide, nb.repeat)

ci.bias.enrichset.suicide <- matrix(0, nb.subset.suicide, 2)

for(i in 1:nb.subset.suicide)

{

if(dim(predict.enrichset.R.suicide)[3] == 1){

bias.enrichset.suicide[i] = mean((predict.enrichset.R.suicide[,i,1] - SOHO.R$CGIS.3month),na.rm=T)

sd.bias.enrichset.suicide[i] = sd((predict.enrichset.R.suicide[,i,1] - SOHO.R$CGIS.3month),na.rm=T)

ci.bias.enrichset.suicide[i,] = c(bias.enrichset.suicide[i]-1.96*sd.bias.enrichset.suicide[i],bias.enrichset.suicide[i]+1.96*sd.bias.enrichset.suicide[i])

} else {

bias.enrichset.suicide[i] = mean((rowMeans(predict.enrichset.R.suicide[,i,]) - SOHO.R$CGIS.3month),na.rm=T)

bias.per.repeat.enrichset.suicide[i,1] = mean((predict.enrichset.R.suicide[,i,1] - SOHO.R$CGIS.3month),na.rm=T)

for(j in 2:nb.repeat){

bias.per.repeat.enrichset.suicide[i,j] = mean((rowMeans(predict.enrichset.R.suicide[,i,1:j]) - SOHO.R$CGIS.3month),na.rm=T)

}

sd.bias.enrichset.suicide[i] = sd(bias.per.repeat.enrichset.suicide[i,],na.rm=T)

ci.bias.enrichset.suicide[i,] = c(bias.enrichset.suicide[i]-1.96*sd.bias.enrichset.suicide[i],bias.enrichset.suicide[i]+1.96*sd.bias.enrichset.suicide[i])

}

}

##################################################################################

### SUBPOPULATION 4: Alcohol abuse

RCT.R.alcohol.table <- c( length(which(is.na(RCT.R$never.alcohol))),table(RCT.R$never.alcohol))

names(RCT.R.alcohol.table)[1]="NA"; names(RCT.R.alcohol.table)[2]="0";

SUBPOP.X.ALCOHOL.R.alcohol.table <- c( length(which(is.na(SUBPOP.X.ALCOHOL.R$never.alcohol))),table(SUBPOP.X.ALCOHOL.R$never.alcohol))

names(SUBPOP.X.ALCOHOL.R.alcohol.table)[1]="NA"; names(SUBPOP.X.ALCOHOL.R.alcohol.table)[2]="1";

SOHO.R.alcohol.table <- c( length(which(is.na(SOHO.R$never.alcohol))),table(SOHO.R$never.alcohol))

names(SOHO.R.alcohol.table)[1]="NA"; names(SOHO.R.alcohol.table)[2]="1"; names(SOHO.R.alcohol.table)[3]="0";

### Augmentation procedure

sub.alcohol.size <- dim(SUBPOP.X.ALCOHOL.R)[1]

enrich.size <- seq(0,sub.alcohol.size,max(round(sub.alcohol.size/100),1))

predict.enrichset.R.alcohol <- array(0, dim=c(length(SOHO.R$CGIS.change.3month), length(enrich.size), nb.repeat))

mean.sd.enrichset.R.alcohol <- array(0, dim=c(2, length(enrich.size), nb.repeat))

enrichset.ID.R.alcohol <- as.list(numeric(length(enrich.size)*nb.repeat)); dim(enrichset.ID.R.alcohol) <- c(length(enrich.size), nb.repeat)

for(l in 1:nb.repeat){

if(l%%100 == 0) print(l)

for(i in 1:length(enrich.size)){

### Prepare the patient ID in X and in the reference RCT

enrich.ID <- sample(SUBPOP.X.ALCOHOL.R$patient.ID, enrich.size[i])

RCT.ID <- sample(RCT.R$patient.ID, size.RCT.target-enrich.size[i])

enrichset.ID.R.alcohol[[i,l]] <- enrichset.R.alcohol.ID <- factor(c(as.character(RCT.ID),as.character(enrich.ID)))

### The data frame of the augmented RCT = enrichset.R.alcohol

enrichset.R.alcohol <- SOHO.R[which(SOHO.R$patient.ID %in% enrichset.R.alcohol.ID),]

### The mean and sd of the CGIS change (from baseline to 3 months) in the augmented RCTs

mean.sd.enrichset.R.alcohol[1,i,l] <- mean(enrichset.R.alcohol$CGIS.3month,na.rm=T)

mean.sd.enrichset.R.alcohol[2,i,l] <- sd(enrichset.R.alcohol$CGIS.3month,na.rm=T)

## MODEL FITTING

enrichset.R.alcohol$CGIS.3month2 =as.factor(enrichset.R.alcohol$CGIS.3month)

opr.enrichset.R.alcohol <- polr(CGIS.3month2 ~ age + duration + BMI + compliance.physician + practice + gender + hospitalization + qualitylife.anxiety + qualitylife.vas + qualitylife.mobility + qualitylife.pain + qualitylife.selfcare + qualitylife.activity + country + work.base + house.base + social.base + spouse.base + negative.symptom + positive.symptom + cognitive.symptom + nb.admit + dosage.R.after + CGIS.baseline, data=enrichset.R.alcohol, method = "logistic", na.action=na.omit)

######## New data for FULL MODEL

SOHO.R.dat <- data.frame(1,age=SOHO.R$age, duration=SOHO.R$duration, BMI=SOHO.R$BMI, compliance.physician=SOHO.R$compliance.physician, practice=SOHO.R$practice, gender=SOHO.R$gender, hospitalization=SOHO.R$hospitalization, qualitylife.anxiety=SOHO.R$qualitylife.anxiety, qualitylife.vas=SOHO.R$qualitylife.vas,qualitylife.mobility=SOHO.R$qualitylife.mobility, qualitylife.pain=SOHO.R$qualitylife.pain, qualitylife.selfcare=SOHO.R$qualitylife.selfcare, qualitylife.activity=SOHO.R$qualitylife.activity, country=SOHO.R$country, work.base=SOHO.R$work.base, house.base=SOHO.R$house.base, social.base=SOHO.R$social.base, spouse.base=SOHO.R$spouse.base, negative.symptom=SOHO.R$negative.symptom, positive.symptom=SOHO.R$positive.symptom, cognitive.symptom=SOHO.R$cognitive.symptom, nb.admit=SOHO.R$nb.admit, dosage.R.after=SOHO.R$dosage.R.after, CGIS.baseline=SOHO.R$CGIS.baseline)

### Prediction in SOHO using the regression model

estimate.coef.alcohol.opr <- as.numeric(suppressMessages(summary(opr.enrichset.R.alcohol))$coefficients[1:24,1])

estimate.Z.alcohol.opr<- as.matrix(SOHO.R.dat[,-1])%*% estimate.coef.alcohol.opr

predict.enrichset.R.alcohol[,i,l] <- ZtoY(estimate.Z.alcohol.opr, alpha=suppressMessages(summary(opr.enrichset.R.alcohol))$coefficients[25:30,1])

}

}

### Check the prediction MSE in each augmented RCT subpopulation

nb.subset.alcohol <- length(enrich.size)

mse.enrichset.alcohol <- sd.mse.enrichset.alcohol <- rep(0, nb.subset.alcohol)

mse.per.repeat.enrichset.alcohol <- matrix(0, nb.subset.alcohol, nb.repeat)

ci.mse.enrichset.alcohol <- matrix(0, nb.subset.alcohol, 2)

for(i in 1:nb.subset.alcohol)

{

if(dim(predict.enrichset.R.alcohol)[3] == 1){

mse.enrichset.alcohol[i] = mean((predict.enrichset.R.alcohol[,i,1] - SOHO.R$CGIS.3month)^2,na.rm=T)

sd.mse.enrichset.alcohol[i] = sd((predict.enrichset.R.alcohol[,i,1] - SOHO.R$CGIS.3month)^2,na.rm=T)

ci.mse.enrichset.alcohol[i,] = c(mse.enrichset.alcohol[i]-1.96*sd.mse.enrichset.alcohol[i],mse.enrichset.alcohol[i]+1.96*sd.mse.enrichset.alcohol[i])

} else {

mse.enrichset.alcohol[i] = mean((rowMeans(predict.enrichset.R.alcohol[,i,]) - SOHO.R$CGIS.3month)^2,na.rm=T)

mse.per.repeat.enrichset.alcohol[i,1] = mean((predict.enrichset.R.alcohol[,i,1] - SOHO.R$CGIS.3month)^2,na.rm=T)

for(j in 2:nb.repeat){

mse.per.repeat.enrichset.alcohol[i,j] = mean((rowMeans(predict.enrichset.R.alcohol[,i,1:j]) - SOHO.R$CGIS.3month)^2,na.rm=T)

}

sd.mse.enrichset.alcohol[i] = sd(mse.per.repeat.enrichset.alcohol[i,],na.rm=T)

ci.mse.enrichset.alcohol[i,] = c(mse.enrichset.alcohol[i]-1.96*sd.mse.enrichset.alcohol[i],mse.enrichset.alcohol[i]+1.96*sd.mse.enrichset.alcohol[i])

}

}

### Check the prediction bias in each augmented RCT subpopulation

bias.enrichset.alcohol <- sd.bias.enrichset.alcohol <- rep(0, nb.subset.alcohol)

bias.per.repeat.enrichset.alcohol <- matrix(0, nb.subset.alcohol, nb.repeat)

ci.bias.enrichset.alcohol <- matrix(0, nb.subset.alcohol, 2)

for(i in 1:nb.subset.alcohol)

{

if(dim(predict.enrichset.R.alcohol)[3] == 1){

bias.enrichset.alcohol[i] = mean((predict.enrichset.R.alcohol[,i,1] - SOHO.R$CGIS.3month),na.rm=T)

sd.bias.enrichset.alcohol[i] = sd((predict.enrichset.R.alcohol[,i,1] - SOHO.R$CGIS.3month),na.rm=T)

ci.bias.enrichset.alcohol[i,] = c(bias.enrichset.alcohol[i]-1.96*sd.bias.enrichset.alcohol[i],bias.enrichset.alcohol[i]+1.96*sd.bias.enrichset.alcohol[i])

} else {

bias.enrichset.alcohol[i] = mean((rowMeans(predict.enrichset.R.alcohol[,i,]) - SOHO.R$CGIS.3month),na.rm=T)

bias.per.repeat.enrichset.alcohol[i,1] = mean((predict.enrichset.R.alcohol[,i,1] - SOHO.R$CGIS.3month),na.rm=T)

for(j in 2:nb.repeat){

bias.per.repeat.enrichset.alcohol[i,j] = mean((rowMeans(predict.enrichset.R.alcohol[,i,1:j]) - SOHO.R$CGIS.3month),na.rm=T)

}

sd.bias.enrichset.alcohol[i] = sd(bias.per.repeat.enrichset.alcohol[i,],na.rm=T)

ci.bias.enrichset.alcohol[i,] = c(bias.enrichset.alcohol[i]-1.96*sd.bias.enrichset.alcohol[i],bias.enrichset.alcohol[i]+1.96*sd.bias.enrichset.alcohol[i])

}

}

##################################################################################

### SUBPOPULATION 5: Drug abuse

RCT.R.substance.table <- c( length(which(is.na(RCT.R$never.substance))),table(RCT.R$never.substance))

names(RCT.R.substance.table)[1]="NA"; names(RCT.R.substance.table)[2]="0";

SUBPOP.X.SUBSTANCE.R.substance.table <- c( length(which(is.na(SUBPOP.X.SUBSTANCE.R$never.substance))),table(SUBPOP.X.SUBSTANCE.R$never.substance))

names(SUBPOP.X.SUBSTANCE.R.substance.table)[1]="NA"; names(SUBPOP.X.SUBSTANCE.R.substance.table)[2]="1";

SOHO.R.substance.table <- c( length(which(is.na(SOHO.R$never.substance))),table(SOHO.R$never.substance))

names(SOHO.R.substance.table)[1]="NA"; names(SOHO.R.substance.table)[2]="1"; names(SOHO.R.substance.table)[3]="0";

### Augmentation procedure

sub.substance.size <- dim(SUBPOP.X.SUBSTANCE.R)[1]

enrich.size <- seq(0,sub.substance.size,max(round(sub.substance.size/100),1))

predict.enrichset.R.substance <- array(0, dim=c(length(SOHO.R$CGIS.change.3month), length(enrich.size), nb.repeat))

mean.sd.enrichset.R.substance <- array(0, dim=c(2, length(enrich.size), nb.repeat))

enrichset.ID.R.substance <- as.list(numeric(length(enrich.size)*nb.repeat)); dim(enrichset.ID.R.substance) <- c(length(enrich.size), nb.repeat)

for(l in 1:nb.repeat){

if(l%%100 == 0) print(l)

for(i in 1:length(enrich.size)){

### Prepare the patient ID in X and in the reference RCT

enrich.ID <- sample(SUBPOP.X.SUBSTANCE.R$patient.ID, enrich.size[i])

RCT.ID <- sample(RCT.R$patient.ID, size.RCT.target-enrich.size[i])

enrichset.ID.R.substance[[i,l]] <- enrichset.R.substance.ID <- factor(c(as.character(RCT.ID),as.character(enrich.ID)))

### The data frame of the augmented RCT = enrichset.R.substance

enrichset.R.substance <- SOHO.R[which(SOHO.R$patient.ID %in% enrichset.R.substance.ID),]

### The mean and sd of the CGIS change (from baseline to 3 months) in the augmented RCTs

mean.sd.enrichset.R.substance[1,i,l] <- mean(enrichset.R.substance$CGIS.3month,na.rm=T)

mean.sd.enrichset.R.substance[2,i,l] <- sd(enrichset.R.substance$CGIS.3month,na.rm=T)

## MODEL FITTING

enrichset.R.substance$CGIS.3month2 =as.factor(enrichset.R.substance$CGIS.3month)

opr.enrichset.R.substance <- polr(CGIS.3month2 ~ age + duration + BMI + compliance.physician + practice + gender + hospitalization + qualitylife.anxiety + qualitylife.vas + qualitylife.mobility + qualitylife.pain + qualitylife.selfcare + qualitylife.activity + country + work.base + house.base + social.base + spouse.base + negative.symptom + positive.symptom + cognitive.symptom + nb.admit + dosage.R.after + CGIS.baseline, data=enrichset.R.substance, method = "logistic", na.action=na.omit)

### Preparation of the new data = SOHO population

SOHO.R.dat <- data.frame(1,age=SOHO.R$age, duration=SOHO.R$duration, BMI=SOHO.R$BMI, compliance.physician=SOHO.R$compliance.physician, practice=SOHO.R$practice, gender=SOHO.R$gender, hospitalization=SOHO.R$hospitalization, qualitylife.anxiety=SOHO.R$qualitylife.anxiety, qualitylife.vas=SOHO.R$qualitylife.vas,qualitylife.mobility=SOHO.R$qualitylife.mobility, qualitylife.pain=SOHO.R$qualitylife.pain, qualitylife.selfcare=SOHO.R$qualitylife.selfcare, qualitylife.activity=SOHO.R$qualitylife.activity, country=SOHO.R$country, work.base=SOHO.R$work.base, house.base=SOHO.R$house.base, social.base=SOHO.R$social.base, spouse.base=SOHO.R$spouse.base, negative.symptom=SOHO.R$negative.symptom, positive.symptom=SOHO.R$positive.symptom, cognitive.symptom=SOHO.R$cognitive.symptom, nb.admit=SOHO.R$nb.admit, dosage.R.after=SOHO.R$dosage.R.after, CGIS.baseline=SOHO.R$CGIS.baseline)

### Prediction in SOHO using the regression model

estimate.coef.substance.opr <- as.numeric(suppressMessages(summary(opr.enrichset.R.substance))$coefficients[1:24,1])

estimate.Z.substance.opr<- as.matrix(SOHO.R.dat[,-1])%*% estimate.coef.substance.opr

predict.enrichset.R.substance[,i,l] <- ZtoY(estimate.Z.substance.opr, alpha=suppressMessages(summary(opr.enrichset.R.substance))$coefficients[25:30,1])

}

}

### Check the prediction MSE in each augmented RCT subpopulation

nb.subset.substance <- length(enrich.size)

mse.enrichset.substance <- sd.mse.enrichset.substance <- rep(0, nb.subset.substance)

mse.per.repeat.enrichset.substance <- matrix(0, nb.subset.substance, nb.repeat)

ci.mse.enrichset.substance <- matrix(0, nb.subset.substance, 2)

for(i in 1:nb.subset.substance)

{

if(dim(predict.enrichset.R.substance)[3] == 1){

mse.enrichset.substance[i] = mean((predict.enrichset.R.substance[,i,1] - SOHO.R$CGIS.3month)^2,na.rm=T)

sd.mse.enrichset.substance[i] = sd((predict.enrichset.R.substance[,i,1] - SOHO.R$CGIS.3month)^2,na.rm=T)

ci.mse.enrichset.substance[i,] = c(mse.enrichset.substance[i]-1.96*sd.mse.enrichset.substance[i],mse.enrichset.substance[i]+1.96*sd.mse.enrichset.substance[i])

} else {

mse.enrichset.substance[i] = mean((rowMeans(predict.enrichset.R.substance[,i,]) - SOHO.R$CGIS.3month)^2,na.rm=T)

mse.per.repeat.enrichset.substance[i,1] = mean((predict.enrichset.R.substance[,i,1] - SOHO.R$CGIS.3month)^2,na.rm=T)

for(j in 2:nb.repeat){

mse.per.repeat.enrichset.substance[i,j] = mean((rowMeans(predict.enrichset.R.substance[,i,1:j]) - SOHO.R$CGIS.3month)^2,na.rm=T)

}

sd.mse.enrichset.substance[i] = sd(mse.per.repeat.enrichset.substance[i,],na.rm=T)

ci.mse.enrichset.substance[i,] = c(mse.enrichset.substance[i]-1.96*sd.mse.enrichset.substance[i],mse.enrichset.substance[i]+1.96*sd.mse.enrichset.substance[i])

}

}

### Check the prediction bias in each augmented RCT subpopulation

bias.enrichset.substance <- sd.bias.enrichset.substance <- rep(0, nb.subset.substance)

bias.per.repeat.enrichset.substance <- matrix(0, nb.subset.substance, nb.repeat)

ci.bias.enrichset.substance <- matrix(0, nb.subset.substance, 2)

for(i in 1:nb.subset.substance)

{

if(dim(predict.enrichset.R.substance)[3] == 1){

bias.enrichset.substance[i] = mean((predict.enrichset.R.substance[,i,1] - SOHO.R$CGIS.3month),na.rm=T)

sd.bias.enrichset.substance[i] = sd((predict.enrichset.R.substance[,i,1] - SOHO.R$CGIS.3month),na.rm=T)

ci.bias.enrichset.substance[i,] = c(bias.enrichset.substance[i]-1.96*sd.bias.enrichset.substance[i],bias.enrichset.substance[i]+1.96*sd.bias.enrichset.substance[i])

} else {

bias.enrichset.substance[i] = mean((rowMeans(predict.enrichset.R.substance[,i,]) - SOHO.R$CGIS.3month),na.rm=T)

bias.per.repeat.enrichset.substance[i,1] = mean((predict.enrichset.R.substance[,i,1] - SOHO.R$CGIS.3month),na.rm=T)

for(j in 2:nb.repeat){

bias.per.repeat.enrichset.substance[i,j] = mean((rowMeans(predict.enrichset.R.substance[,i,1:j]) - SOHO.R$CGIS.3month),na.rm=T)

}

sd.bias.enrichset.substance[i] = sd(bias.per.repeat.enrichset.substance[i,],na.rm=T)

ci.bias.enrichset.substance[i,] = c(bias.enrichset.substance[i]-1.96*sd.bias.enrichset.substance[i],bias.enrichset.substance[i]+1.96*sd.bias.enrichset.substance[i])

}

}

##################################################################################

### SUBPOPULATION 6: Illness duration or Suicide attempt

### Definition of subpopulations of type X and Y: patients who have Illness duration 1-3 years or 1 past suicide attempt

SUBPOP.X.SUICIDE.DURATION.R$suicidegroup <- cut(as.numeric(SUBPOP.X.SUICIDE.DURATION.R$suicide.base), breaks=c(-0.5, 0.5, 1.5, 5.5, 60), labels=c("0", "1", "1-5","6+"))

RCT.R$suicidegroup <- cut(as.numeric(RCT.R$suicide.base), breaks=c(-0.5, 0.5, 1.5, 5.5, 60), labels=c("0", "1", "1-5","6+"))

SOHO.R$suicidegroup <- cut(as.numeric(SOHO.R$suicide.base), breaks=c(-0.5, 0.5, 1.5, 5.5, 60), labels=c("0", "1", "1-5","6+"))

SUBPOP.X.SUICIDE.DURATION.R$durationgroup <- cut(as.numeric(SUBPOP.X.SUICIDE.DURATION.R$duration), breaks=c(0,1,3, seq(15, 55, 10),65), labels=c("0-1","1-3","3-15", "15-25", "25-35", "35-45", "45-55", "55-65"))

RCT.R$durationgroup <- cut(as.numeric(RCT.R$duration), breaks=c(0,1,3, seq(15, 55, 10),65), labels=c("0-1","1-3","3-15", "15-25", "25-35", "35-45", "45-55", "55-65"))

SOHO.R$durationgroup <- cut(as.numeric(SOHO.R$duration), breaks=c(0,1,3, seq(15, 55, 10),65), labels=c("0-1","1-3","3-15", "15-25", "25-35", "35-45", "45-55", "55-65"))

RCT.R.suicidegroup.table <- c( length(which(is.na(RCT.R$suicide.base))),table(RCT.R$suicidegroup))

names(RCT.R.suicidegroup.table)[1]="NA"

SUBPOP.X.SUICIDE.DURATION.R.suicidegroup.table <- c( length(which(is.na(SUBPOP.X.SUICIDE.DURATION.R$suicide.base))),table(SUBPOP.X.SUICIDE.DURATION.R$suicidegroup))

names(SUBPOP.X.SUICIDE.DURATION.R.suicidegroup.table)[1]="NA"

SOHO.R.suicidegroup.table <- c( length(which(is.na(SOHO.R$suicide.base))),table(SOHO.R$suicidegroup))

names(SOHO.R.suicidegroup.table)[1]="NA"

RCT.R.durationgroup.table <- c( length(which(is.na(RCT.R$duration))),table(RCT.R$durationgroup))

names(RCT.R.durationgroup.table)[1]="NA"

SUBPOP.X.SUICIDE.DURATION.R.durationgroup.table <- c( length(which(is.na(SUBPOP.X.SUICIDE.DURATION.R$duration))),table(SUBPOP.X.SUICIDE.DURATION.R$durationgroup))

names(SUBPOP.X.SUICIDE.DURATION.R.durationgroup.table)[1]="NA"

SOHO.R.durationgroup.table <- c( length(which(is.na(SOHO.R$duration))),table(SOHO.R$durationgroup))

names(SOHO.R.durationgroup.table)[1]="NA"

### Augmentation procedure

sub.suicide.duration.size <- dim(SUBPOP.X.SUICIDE.DURATION.R)[1]

enrich.size <- seq(0,sub.suicide.duration.size,max(round(sub.suicide.duration.size/100),1))

predict.enrichset.R.suicide.duration <- array(0, dim=c(length(SOHO.R$CGIS.change.3month), length(enrich.size), nb.repeat))

mean.sd.enrichset.R.suicide.duration <- array(0, dim=c(2, length(enrich.size), nb.repeat))

enrichset.ID.R.suicide.duration <- as.list(numeric(length(enrich.size)*nb.repeat)); dim(enrichset.ID.R.suicide.duration) <- c(length(enrich.size), nb.repeat)

for(l in 1:nb.repeat){

if(l%%100 == 0) print(l)

for(i in 1:length(enrich.size)){

### Prepare the patient ID in X and in the reference RCT

enrich.ID <- sample(SUBPOP.X.SUICIDE.DURATION.R$patient.ID, enrich.size[i])

RCT.ID <- sample(RCT.R$patient.ID, size.RCT.target-enrich.size[i])

enrichset.ID.R.suicide.duration[[i,l]] <- enrichset.R.suicide.duration.ID <- factor(c(as.character(RCT.ID),as.character(enrich.ID)))

### The data frame of the augmented RCT = enrichset.R.suicide

enrichset.R.suicide.duration <- SOHO.R[which(SOHO.R$patient.ID %in% enrichset.R.suicide.duration.ID),]

### The mean and sd of the CGIS change (from baseline to 3 months) in the augmented RCTs

mean.sd.enrichset.R.suicide.duration[1,i,l] <- mean(enrichset.R.suicide.duration$CGIS.3month,na.rm=T)

mean.sd.enrichset.R.suicide.duration[2,i,l] <- sd(enrichset.R.suicide.duration$CGIS.3month,na.rm=T)

## MODEL FITTING

enrichset.R.suicide.duration$CGIS.3month2 =as.factor(enrichset.R.suicide.duration$CGIS.3month)

opr.enrichset.R.suicide.duration <- polr(CGIS.3month2 ~ age + duration + BMI + compliance.physician + practice + gender + hospitalization + qualitylife.anxiety + qualitylife.vas + qualitylife.mobility + qualitylife.pain + qualitylife.selfcare + qualitylife.activity + country + work.base + house.base + social.base + spouse.base + negative.symptom + positive.symptom + cognitive.symptom + nb.admit + dosage.R.after + CGIS.baseline, data=enrichset.R.suicide.duration, method = "logistic", na.action=na.omit)

### Preparation of the new data = SOHO population

SOHO.R.dat <- data.frame(1,age=SOHO.R$age, duration=SOHO.R$duration, BMI=SOHO.R$BMI, compliance.physician=SOHO.R$compliance.physician, practice=SOHO.R$practice, gender=SOHO.R$gender, hospitalization=SOHO.R$hospitalization, qualitylife.anxiety=SOHO.R$qualitylife.anxiety, qualitylife.vas=SOHO.R$qualitylife.vas,qualitylife.mobility=SOHO.R$qualitylife.mobility, qualitylife.pain=SOHO.R$qualitylife.pain, qualitylife.selfcare=SOHO.R$qualitylife.selfcare, qualitylife.activity=SOHO.R$qualitylife.activity, country=SOHO.R$country, work.base=SOHO.R$work.base, house.base=SOHO.R$house.base, social.base=SOHO.R$social.base, spouse.base=SOHO.R$spouse.base, negative.symptom=SOHO.R$negative.symptom, positive.symptom=SOHO.R$positive.symptom, cognitive.symptom=SOHO.R$cognitive.symptom, nb.admit=SOHO.R$nb.admit, dosage.R.after=SOHO.R$dosage.R.after, CGIS.baseline=SOHO.R$CGIS.baseline)

### Prediction in SOHO using the regression model

estimate.coef.suicide.duration.opr <- as.numeric(suppressMessages(summary(opr.enrichset.R.suicide.duration))$coefficients[1:24,1])

estimate.Z.suicide.duration.opr<- as.matrix(SOHO.R.dat[,-1])%*% estimate.coef.suicide.duration.opr

predict.enrichset.R.suicide.duration[,i,l] <- ZtoY(estimate.Z.suicide.duration.opr, alpha=suppressMessages(summary(opr.enrichset.R.suicide.duration))$coefficients[25:30,1])

}

}

### Check the prediction MSE in each augmented RCT subpopulation

nb.subset.suicide.duration <- length(enrich.size)

mse.enrichset.suicide.duration <- sd.mse.enrichset.suicide.duration <- rep(0, nb.subset.suicide.duration)

mse.per.repeat.enrichset.suicide.duration <- matrix(0, nb.subset.suicide.duration, nb.repeat)

ci.mse.enrichset.suicide.duration <- matrix(0, nb.subset.suicide.duration, 2)

for(i in 1:nb.subset.suicide.duration)

{

if(dim(predict.enrichset.R.suicide.duration)[3] == 1){

mse.enrichset.suicide.duration[i] = mean((predict.enrichset.R.suicide.duration[,i,1] - SOHO.R$CGIS.3month)^2,na.rm=T)

sd.mse.enrichset.suicide.duration[i] = sd((predict.enrichset.R.suicide.duration[,i,1] - SOHO.R$CGIS.3month)^2,na.rm=T)

ci.mse.enrichset.suicide.duration[i,] = c(mse.enrichset.suicide.duration[i]-1.96*sd.mse.enrichset.suicide.duration[i],mse.enrichset.suicide.duration[i]+1.96*sd.mse.enrichset.suicide.duration[i])

} else {

mse.enrichset.suicide.duration[i] = mean((rowMeans(predict.enrichset.R.suicide.duration[,i,]) - SOHO.R$CGIS.3month)^2,na.rm=T)

mse.per.repeat.enrichset.suicide.duration[i,1] = mean((predict.enrichset.R.suicide.duration[,i,1] - SOHO.R$CGIS.3month)^2,na.rm=T)

for(j in 2:nb.repeat){

mse.per.repeat.enrichset.suicide.duration[i,j] = mean((rowMeans(predict.enrichset.R.suicide.duration[,i,1:j]) - SOHO.R$CGIS.3month)^2,na.rm=T)

}

sd.mse.enrichset.suicide.duration[i] = sd(mse.per.repeat.enrichset.suicide.duration[i,],na.rm=T)

ci.mse.enrichset.suicide.duration[i,] = c(mse.enrichset.suicide.duration[i]-1.96*sd.mse.enrichset.suicide.duration[i],mse.enrichset.suicide.duration[i]+1.96*sd.mse.enrichset.suicide.duration[i])

}

}

### Check the prediction bias in each augmented RCT subpopulation

bias.enrichset.suicide.duration <- sd.bias.enrichset.suicide.duration <- rep(0, nb.subset.suicide.duration)

bias.per.repeat.enrichset.suicide.duration <- matrix(0, nb.subset.suicide.duration, nb.repeat)

ci.bias.enrichset.suicide.duration <- matrix(0, nb.subset.suicide.duration, 2)

for(i in 1:nb.subset.suicide.duration)

{

if(dim(predict.enrichset.R.suicide.duration)[3] == 1){

bias.enrichset.suicide.duration[i] = mean((predict.enrichset.R.suicide.duration[,i,1] - SOHO.R$CGIS.3month),na.rm=T)

sd.bias.enrichset.suicide.duration[i] = sd((predict.enrichset.R.suicide.duration[,i,1] - SOHO.R$CGIS.3month),na.rm=T)

ci.bias.enrichset.suicide.duration[i,] = c(bias.enrichset.suicide.duration[i]-1.96*sd.bias.enrichset.suicide.duration[i],bias.enrichset.suicide.duration[i]+1.96*sd.bias.enrichset.suicide.duration[i])

} else {

bias.enrichset.suicide.duration[i] = mean((rowMeans(predict.enrichset.R.suicide.duration[,i,]) - SOHO.R$CGIS.3month),na.rm=T)

bias.per.repeat.enrichset.suicide.duration[i,1] = mean((predict.enrichset.R.suicide.duration[,i,1] - SOHO.R$CGIS.3month),na.rm=T)

for(j in 2:nb.repeat){

bias.per.repeat.enrichset.suicide.duration[i,j] = mean((rowMeans(predict.enrichset.R.suicide.duration[,i,1:j]) - SOHO.R$CGIS.3month),na.rm=T)

}

sd.bias.enrichset.suicide.duration[i] = sd(bias.per.repeat.enrichset.suicide.duration[i,],na.rm=T)

ci.bias.enrichset.suicide.duration[i,] = c(bias.enrichset.suicide.duration[i]-1.96*sd.bias.enrichset.suicide.duration[i],bias.enrichset.suicide.duration[i]+1.96*sd.bias.enrichset.suicide.duration[i])

}

}

##################################################################################

### SUBPOPULATION 7: Alcohol or Illness duration

### Definition of subpopulations of type X and Y: patients who have Illness duration 1-3 years or alcohol abuse

SUBPOP.X.DURATION.ALCOHOL.R$durationgroup <- cut(as.numeric(SUBPOP.X.DURATION.ALCOHOL.R$duration), breaks=c(0,1,3, seq(15, 55, 10),65), labels=c("0-1","1-3","3-15", "15-25", "25-35", "35-45", "45-55", "55-65"))

RCT.R$durationgroup <- cut(as.numeric(RCT.R$duration), breaks=c(0,1,3, seq(15, 55, 10),65), labels=c("0-1","1-3","3-15", "15-25", "25-35", "35-45", "45-55", "55-65"))

SOHO.R$durationgroup <- cut(as.numeric(SOHO.R$duration), breaks=c(0,1,3, seq(15, 55, 10),65), labels=c("0-1","1-3","3-15", "15-25", "25-35", "35-45", "45-55", "55-65"))

RCT.R.durationgroup.table <- c( length(which(is.na(RCT.R$duration))),table(RCT.R$durationgroup))

names(RCT.R.durationgroup.table)[1]="NA"

SUBPOP.X.DURATION.ALCOHOL.R.durationgroup.table <- c( length(which(is.na(SUBPOP.X.DURATION.ALCOHOL.R$duration))),table(SUBPOP.X.DURATION.ALCOHOL.R$durationgroup))

names(SUBPOP.X.DURATION.ALCOHOL.R.durationgroup.table)[1]="NA"

SOHO.R.durationgroup.table <- c( length(which(is.na(SOHO.R$duration))),table(SOHO.R$durationgroup))

names(SOHO.R.durationgroup.table)[1]="NA"

RCT.R.alcohol.table <- c( length(which(is.na(RCT.R$never.alcohol))),table(RCT.R$never.alcohol))

names(RCT.R.alcohol.table)[1]="NA"; names(RCT.R.alcohol.table)[2]="0";

SUBPOP.X.DURATION.ALCOHOL.R.alcohol.table <- c( length(which(is.na(SUBPOP.X.DURATION.ALCOHOL.R$never.alcohol))),table(SUBPOP.X.DURATION.ALCOHOL.R$never.alcohol))

names(SUBPOP.X.DURATION.ALCOHOL.R.alcohol.table)[1]="NA"; names(SUBPOP.X.DURATION.ALCOHOL.R.alcohol.table)[2]="1"; names(SUBPOP.X.DURATION.ALCOHOL.R.alcohol.table)[3]="0";

SOHO.R.alcohol.table <- c( length(which(is.na(SOHO.R$never.alcohol))),table(SOHO.R$never.alcohol))

names(SOHO.R.alcohol.table)[1]="NA"; names(SOHO.R.alcohol.table)[2]="1"; names(SOHO.R.alcohol.table)[3]="0";

### Augmentation procedure

sub.duration.alcohol.size <- dim(SUBPOP.X.DURATION.ALCOHOL.R)[1]

enrich.size <- seq(0,sub.duration.alcohol.size,max(round(sub.duration.alcohol.size/100),1))

predict.enrichset.R.duration.alcohol <- array(0, dim=c(length(SOHO.R$CGIS.change.3month), length(enrich.size), nb.repeat))

mean.sd.enrichset.R.duration.alcohol <- array(0, dim=c(2, length(enrich.size), nb.repeat))

enrichset.ID.R.duration.alcohol <- as.list(numeric(length(enrich.size)*nb.repeat)); dim(enrichset.ID.R.duration.alcohol) <- c(length(enrich.size), nb.repeat)

for(l in 1:nb.repeat){

if(l%%100 == 0) print(l)

for(i in 1:length(enrich.size))

### Prepare the patient ID in X and in the reference RCT

enrich.ID <- sample(SUBPOP.X.DURATION.ALCOHOL.R$patient.ID, enrich.size[i])

RCT.ID <- sample(RCT.R$patient.ID, size.RCT.target-enrich.size[i])

enrichset.ID.R.duration.alcohol[[i,l]] <- enrichset.R.duration.alcohol.ID <- factor(c(as.character(RCT.ID),as.character(enrich.ID)))

### The data frame of the augmented RCT = enrichset.R.duration

enrichset.R.duration.alcohol <- SOHO.R[which(SOHO.R$patient.ID %in% enrichset.R.duration.alcohol.ID),]

### The mean and sd of the CGIS change (from baseline to 3 months) in the augmented RCTs

mean.sd.enrichset.R.duration.alcohol[1,i,l] <- mean(enrichset.R.duration.alcohol$CGIS.3month,na.rm=T)

mean.sd.enrichset.R.duration.alcohol[2,i,l] <- sd(enrichset.R.duration.alcohol$CGIS.3month,na.rm=T)

## MODEL FITTING

enrichset.R.duration.alcohol$CGIS.3month2 =as.factor(enrichset.R.duration.alcohol$CGIS.3month)

opr.enrichset.R.duration.alcohol <- polr(CGIS.3month2 ~ age + duration + BMI + compliance.physician + practice + gender + hospitalization + qualitylife.anxiety + qualitylife.vas + qualitylife.mobility + qualitylife.pain + qualitylife.selfcare + qualitylife.activity + country + work.base + house.base + social.base + spouse.base + negative.symptom + positive.symptom + cognitive.symptom + nb.admit + dosage.R.after + CGIS.baseline, data=enrichset.R.duration.alcohol, method = "logistic", na.action=na.omit)

### Preparation of the new data = SOHO population

SOHO.R.dat <- data.frame(1,age=SOHO.R$age, duration=SOHO.R$duration, BMI=SOHO.R$BMI, compliance.physician=SOHO.R$compliance.physician, practice=SOHO.R$practice, gender=SOHO.R$gender, hospitalization=SOHO.R$hospitalization, qualitylife.anxiety=SOHO.R$qualitylife.anxiety, qualitylife.vas=SOHO.R$qualitylife.vas,qualitylife.mobility=SOHO.R$qualitylife.mobility, qualitylife.pain=SOHO.R$qualitylife.pain, qualitylife.selfcare=SOHO.R$qualitylife.selfcare, qualitylife.activity=SOHO.R$qualitylife.activity, country=SOHO.R$country, work.base=SOHO.R$work.base, house.base=SOHO.R$house.base, social.base=SOHO.R$social.base, spouse.base=SOHO.R$spouse.base, negative.symptom=SOHO.R$negative.symptom, positive.symptom=SOHO.R$positive.symptom, cognitive.symptom=SOHO.R$cognitive.symptom, nb.admit=SOHO.R$nb.admit, dosage.R.after=SOHO.R$dosage.R.after, CGIS.baseline=SOHO.R$CGIS.baseline)

### Prediction in SOHO using the regression model

estimate.coef.duration.alcohol.opr <- as.numeric(suppressMessages(summary(opr.enrichset.R.duration.alcohol))$coefficients[1:24,1])

estimate.Z.duration.alcohol.opr<- as.matrix(SOHO.R.dat[,-1])%*% estimate.coef.duration.alcohol.opr

predict.enrichset.R.duration.alcohol[,i,l] <- ZtoY(estimate.Z.duration.alcohol.opr, alpha=suppressMessages(summary(opr.enrichset.R.duration.alcohol))$coefficients[25:30,1])

}

}

### Check the prediction MSE in each augmented RCT subpopulation

nb.subset.duration.alcohol <- length(enrich.size)

mse.enrichset.duration.alcohol <- sd.mse.enrichset.duration.alcohol <- rep(0, nb.subset.duration.alcohol)

mse.per.repeat.enrichset.duration.alcohol <- matrix(0, nb.subset.duration.alcohol, nb.repeat)

ci.mse.enrichset.duration.alcohol <- matrix(0, nb.subset.duration.alcohol, 2)

for(i in 1:nb.subset.duration.alcohol)

{

if(dim(predict.enrichset.R.duration.alcohol)[3] == 1){

mse.enrichset.duration.alcohol[i] = mean((predict.enrichset.R.duration.alcohol[,i,1] - SOHO.R$CGIS.3month)^2,na.rm=T)

sd.mse.enrichset.duration.alcohol[i] = sd((predict.enrichset.R.duration.alcohol[,i,1] - SOHO.R$CGIS.3month)^2,na.rm=T)

ci.mse.enrichset.duration.alcohol[i,] = c(mse.enrichset.duration.alcohol[i]-1.96*sd.mse.enrichset.duration.alcohol[i],mse.enrichset.duration.alcohol[i]+1.96*sd.mse.enrichset.duration.alcohol[i])

} else {

mse.enrichset.duration.alcohol[i] = mean((rowMeans(predict.enrichset.R.duration.alcohol[,i,]) - SOHO.R$CGIS.3month)^2,na.rm=T)

mse.per.repeat.enrichset.duration.alcohol[i,1] = mean((predict.enrichset.R.duration.alcohol[,i,1] - SOHO.R$CGIS.3month)^2,na.rm=T)

for(j in 2:nb.repeat){

mse.per.repeat.enrichset.duration.alcohol[i,j] = mean((rowMeans(predict.enrichset.R.duration.alcohol[,i,1:j]) - SOHO.R$CGIS.3month)^2,na.rm=T)

}

sd.mse.enrichset.duration.alcohol[i] = sd(mse.per.repeat.enrichset.duration.alcohol[i,],na.rm=T)

ci.mse.enrichset.duration.alcohol[i,] = c(mse.enrichset.duration.alcohol[i]-1.96*sd.mse.enrichset.duration.alcohol[i],mse.enrichset.duration.alcohol[i]+1.96*sd.mse.enrichset.duration.alcohol[i])

}

}

### Check the prediction bias in each augmented RCT subpopulation

bias.enrichset.duration.alcohol <- sd.bias.enrichset.duration.alcohol <- rep(0, nb.subset.duration.alcohol)

bias.per.repeat.enrichset.duration.alcohol <- matrix(0, nb.subset.duration.alcohol, nb.repeat)

ci.bias.enrichset.duration.alcohol <- matrix(0, nb.subset.duration.alcohol, 2)

for(i in 1:nb.subset.duration.alcohol)

{

if(dim(predict.enrichset.R.duration.alcohol)[3] == 1){

bias.enrichset.duration.alcohol[i] = mean((predict.enrichset.R.duration.alcohol[,i,1] - SOHO.R$CGIS.3month),na.rm=T)

sd.bias.enrichset.duration.alcohol[i] = sd((predict.enrichset.R.duration.alcohol[,i,1] - SOHO.R$CGIS.3month),na.rm=T)

ci.bias.enrichset.duration.alcohol[i,] = c(bias.enrichset.duration.alcohol[i]-1.96*sd.bias.enrichset.duration.alcohol[i],bias.enrichset.duration.alcohol[i]+1.96*sd.bias.enrichset.duration.alcohol[i])

} else {

bias.enrichset.duration.alcohol[i] = mean((rowMeans(predict.enrichset.R.duration.alcohol[,i,]) - SOHO.R$CGIS.3month),na.rm=T)

bias.per.repeat.enrichset.duration.alcohol[i,1] = mean((predict.enrichset.R.duration.alcohol[,i,1] - SOHO.R$CGIS.3month),na.rm=T)

for(j in 2:nb.repeat){

bias.per.repeat.enrichset.duration.alcohol[i,j] = mean((rowMeans(predict.enrichset.R.duration.alcohol[,i,1:j]) - SOHO.R$CGIS.3month),na.rm=T)

}

sd.bias.enrichset.duration.alcohol[i] = sd(bias.per.repeat.enrichset.duration.alcohol[i,],na.rm=T)

ci.bias.enrichset.duration.alcohol[i,] = c(bias.enrichset.duration.alcohol[i]-1.96*sd.bias.enrichset.duration.alcohol[i],bias.enrichset.duration.alcohol[i]+1.96*sd.bias.enrichset.duration.alcohol[i])

}

}

##################################################################################

### SUBPOPULATION 8: Practice or Illness duration

### Definition of subpopulations of type X and Y: patients who have Illness duration 1-3 years or private practice

SUBPOP.X.DURATION.PRACTICE.R$durationgroup <- cut(as.numeric(SUBPOP.X.DURATION.PRACTICE.R$duration), breaks=c(0,1,3, seq(15, 55, 10),65), labels=c("0-1","1-3","3-15", "15-25", "25-35", "35-45", "45-55", "55-65"))

RCT.R$durationgroup <- cut(as.numeric(RCT.R$duration), breaks=c(0,1,3, seq(15, 55, 10),65), labels=c("0-1","1-3","3-15", "15-25", "25-35", "35-45", "45-55", "55-65"))

SOHO.R$durationgroup <- cut(as.numeric(SOHO.R$duration), breaks=c(0,1,3, seq(15, 55, 10),65), labels=c("0-1","1-3","3-15", "15-25", "25-35", "35-45", "45-55", "55-65"))

RCT.R.durationgroup.table <- c( length(which(is.na(RCT.R$duration))),table(RCT.R$durationgroup))

names(RCT.R.durationgroup.table)[1]="NA"

SUBPOP.X.DURATION.PRACTICE.R.durationgroup.table <- c( length(which(is.na(SUBPOP.X.DURATION.PRACTICE.R$duration))),table(SUBPOP.X.DURATION.PRACTICE.R$durationgroup))

names(SUBPOP.X.DURATION.PRACTICE.R.durationgroup.table)[1]="NA"

SOHO.R.durationgroup.table <- c( length(which(is.na(SOHO.R$duration))),table(SOHO.R$durationgroup))

names(SOHO.R.durationgroup.table)[1]="NA"

RCT.R.practice.table <- SUBPOP.X.DURATION.PRACTICE.R.practice.table <- SOHO.R.practice.table <- rep(0,4)

RCT.R.practice.table[c(2,4)] <- table(RCT.R$practice)

RCT.R.practice.table[1] <- length(which(is.na(RCT.R$practice)))

names(RCT.R.practice.table)[1]="NA"; names(RCT.R.practice.table)[2]="Public"; names(RCT.R.practice.table)[3]="Private"; names(RCT.R.practice.table)[4]="Combined";

SUBPOP.X.DURATION.PRACTICE.R.practice.table[2:4] <- table(SUBPOP.X.DURATION.PRACTICE.R$practice)

SUBPOP.X.DURATION.PRACTICE.R.practice.table[1] <- length(which(is.na(SUBPOP.X.DURATION.PRACTICE.R$practice)))

names(SUBPOP.X.DURATION.PRACTICE.R.practice.table)[1]="NA"; names(SUBPOP.X.DURATION.PRACTICE.R.practice.table)[2]="Public"; names(SUBPOP.X.DURATION.PRACTICE.R.practice.table)[3]="Private"; names(SUBPOP.X.DURATION.PRACTICE.R.practice.table)[4]="Combined";

SOHO.R.practice.table[2:4] <- table(SOHO.R$practice)

SOHO.R.practice.table[1] <- length(which(is.na(SOHO.R$practice)))

names(SOHO.R.practice.table)[1]="NA"; names(SOHO.R.practice.table)[2]="Public"; names(SOHO.R.practice.table)[3]="Private"; names(SOHO.R.practice.table)[4]="Combined";

### Augmentation procedure

sub.duration.practice.size <- dim(SUBPOP.X.DURATION.PRACTICE.R)[1]

enrich.size <- seq(0,sub.duration.practice.size,max(round(sub.duration.practice.size/100),1))

predict.enrichset.R.duration.practice <- array(0, dim=c(length(SOHO.R$CGIS.change.3month), length(enrich.size), nb.repeat))

mean.sd.enrichset.R.duration.practice <- array(0, dim=c(2, length(enrich.size), nb.repeat))

enrichset.ID.R.duration.practice <- as.list(numeric(length(enrich.size)*nb.repeat)); dim(enrichset.ID.R.duration.practice) <- c(length(enrich.size), nb.repeat)

for(l in 1:nb.repeat){

if(l%%100 == 0) print(l)

for(i in 1:length(enrich.size)){

### Prepare the patient ID in X and in the reference RCT

enrich.ID <- sample(SUBPOP.X.DURATION.PRACTICE.R$patient.ID, enrich.size[i])

RCT.ID <- sample(RCT.R$patient.ID, size.RCT.target-enrich.size[i])

enrichset.ID.R.duration.practice[[i,l]] <- enrichset.R.duration.practice.ID <- factor(c(as.character(RCT.ID),as.character(enrich.ID)))

### The data frame of the augmented RCT = enrichset.R.duration

enrichset.R.duration.practice <- SOHO.R[which(SOHO.R$patient.ID %in% enrichset.R.duration.practice.ID),]

### The mean and sd of the CGIS change (from baseline to 3 months) in the augmented RCTs

mean.sd.enrichset.R.duration.practice[1,i,l] <- mean(enrichset.R.duration.practice$CGIS.3month,na.rm=T)

mean.sd.enrichset.R.duration.practice[2,i,l] <- sd(enrichset.R.duration.practice$CGIS.3month,na.rm=T)

## MODEL FITTING

enrichset.R.duration.practice$CGIS.3month2 =as.factor(enrichset.R.duration.practice$CGIS.3month)

opr.enrichset.R.duration.practice <- polr(CGIS.3month2 ~ age + duration + BMI + compliance.physician + practice + gender + hospitalization + qualitylife.anxiety + qualitylife.vas + qualitylife.mobility + qualitylife.pain + qualitylife.selfcare + qualitylife.activity + country + work.base + house.base + social.base + spouse.base + negative.symptom + positive.symptom + cognitive.symptom + nb.admit + dosage.R.after + CGIS.baseline, data=enrichset.R.duration.practice, method = "logistic", na.action=na.omit)

### Preparation of the new data = SOHO population

SOHO.R.dat <- data.frame(1,age=SOHO.R$age, duration=SOHO.R$duration, BMI=SOHO.R$BMI, compliance.physician=SOHO.R$compliance.physician, practice=SOHO.R$practice, gender=SOHO.R$gender, hospitalization=SOHO.R$hospitalization, qualitylife.anxiety=SOHO.R$qualitylife.anxiety, qualitylife.vas=SOHO.R$qualitylife.vas,qualitylife.mobility=SOHO.R$qualitylife.mobility, qualitylife.pain=SOHO.R$qualitylife.pain, qualitylife.selfcare=SOHO.R$qualitylife.selfcare, qualitylife.activity=SOHO.R$qualitylife.activity, country=SOHO.R$country, work.base=SOHO.R$work.base, house.base=SOHO.R$house.base, social.base=SOHO.R$social.base, spouse.base=SOHO.R$spouse.base, negative.symptom=SOHO.R$negative.symptom, positive.symptom=SOHO.R$positive.symptom, cognitive.symptom=SOHO.R$cognitive.symptom, nb.admit=SOHO.R$nb.admit, dosage.R.after=SOHO.R$dosage.R.after, CGIS.baseline=SOHO.R$CGIS.baseline)

### Prediction in SOHO using the regression model

estimate.coef.duration.practice.opr <- as.numeric(suppressMessages(summary(opr.enrichset.R.duration.practice))$coefficients[1:24,1])

estimate.Z.duration.practice.opr<- as.matrix(SOHO.R.dat[,-1])%*% estimate.coef.duration.practice.opr

predict.enrichset.R.duration.practice[,i,l] <- ZtoY(estimate.Z.duration.practice.opr, alpha=suppressMessages(summary(opr.enrichset.R.duration.practice))$coefficients[25:30,1])

}

}

### Check the prediction MSE in each augmented RCT subpopulation

nb.subset.duration.practice <- length(enrich.size)

mse.enrichset.duration.practice <- sd.mse.enrichset.duration.practice <- rep(0, nb.subset.duration.practice)

mse.per.repeat.enrichset.duration.practice <- matrix(0, nb.subset.duration.practice, nb.repeat)

ci.mse.enrichset.duration.practice <- matrix(0, nb.subset.duration.practice, 2)

for(i in 1:nb.subset.duration.practice)

{

if(dim(predict.enrichset.R.duration.practice)[3] == 1){

mse.enrichset.duration.practice[i] = mean((predict.enrichset.R.duration.practice[,i,1] - SOHO.R$CGIS.3month)^2,na.rm=T)

sd.mse.enrichset.duration.practice[i] = sd((predict.enrichset.R.duration.practice[,i,1] - SOHO.R$CGIS.3month)^2,na.rm=T)

ci.mse.enrichset.duration.practice[i,] = c(mse.enrichset.duration.practice[i]-1.96*sd.mse.enrichset.duration.practice[i],mse.enrichset.duration.practice[i]+1.96*sd.mse.enrichset.duration.practice[i])

} else {

mse.enrichset.duration.practice[i] = mean((rowMeans(predict.enrichset.R.duration.practice[,i,]) - SOHO.R$CGIS.3month)^2,na.rm=T)

mse.per.repeat.enrichset.duration.practice[i,1] = mean((predict.enrichset.R.duration.practice[,i,1] - SOHO.R$CGIS.3month)^2,na.rm=T)

for(j in 2:nb.repeat){

mse.per.repeat.enrichset.duration.practice[i,j] = mean((rowMeans(predict.enrichset.R.duration.practice[,i,1:j]) - SOHO.R$CGIS.3month)^2,na.rm=T)

}

sd.mse.enrichset.duration.practice[i] = sd(mse.per.repeat.enrichset.duration.practice[i,],na.rm=T)

ci.mse.enrichset.duration.practice[i,] = c(mse.enrichset.duration.practice[i]-1.96*sd.mse.enrichset.duration.practice[i],mse.enrichset.duration.practice[i]+1.96*sd.mse.enrichset.duration.practice[i])

}

}

### Check the prediction bias in each augmented RCT subpopulation

bias.enrichset.duration.practice <- sd.bias.enrichset.duration.practice <- rep(0, nb.subset.duration.practice)

bias.per.repeat.enrichset.duration.practice <- matrix(0, nb.subset.duration.practice, nb.repeat)

ci.bias.enrichset.duration.practice <- matrix(0, nb.subset.duration.practice, 2)

for(i in 1:nb.subset.duration.practice)

{

if(dim(predict.enrichset.R.duration.practice)[3] == 1){

bias.enrichset.duration.practice[i] = mean((predict.enrichset.R.duration.practice[,i,1] - SOHO.R$CGIS.3month),na.rm=T)

sd.bias.enrichset.duration.practice[i] = sd((predict.enrichset.R.duration.practice[,i,1] - SOHO.R$CGIS.3month),na.rm=T)

ci.bias.enrichset.duration.practice[i,] = c(bias.enrichset.duration.practice[i]-1.96*sd.bias.enrichset.duration.practice[i],bias.enrichset.duration.practice[i]+1.96*sd.bias.enrichset.duration.practice[i])

} else {

bias.enrichset.duration.practice[i] = mean((rowMeans(predict.enrichset.R.duration.practice[,i,]) - SOHO.R$CGIS.3month),na.rm=T)

bias.per.repeat.enrichset.duration.practice[i,1] = mean((predict.enrichset.R.duration.practice[,i,1] - SOHO.R$CGIS.3month),na.rm=T)

for(j in 2:nb.repeat){

bias.per.repeat.enrichset.duration.practice[i,j] = mean((rowMeans(predict.enrichset.R.duration.practice[,i,1:j]) - SOHO.R$CGIS.3month),na.rm=T)

}

sd.bias.enrichset.duration.practice[i] = sd(bias.per.repeat.enrichset.duration.practice[i,],na.rm=T)

ci.bias.enrichset.duration.practice[i,] = c(bias.enrichset.duration.practice[i]-1.96*sd.bias.enrichset.duration.practice[i],bias.enrichset.duration.practice[i]+1.96*sd.bias.enrichset.duration.practice[i])

}

}

### Code used to simulate virtual RCTs and calculate comparative efficacy

###########################################################

#### Load data and imputed data

###########################################################

set.seed(42)

load("sohodata.RData")

load("imputedSOHObaseline_full.RData")

###############################################################

###Imputed dataset preparation

###############################################################

SOHO.patientID.RAE <- unique(mydata$patient.ID)[which(R.flag+AE.flag == 1)]

SOHO.index.RAE <- which(unique(mydata$patient.ID) %in% SOHO.patientID.RAE)

imputed.SOHO.mydata.baseline.RAE <- imputed.SOHO.baseline[SOHO.index.RAE, ]

imputed.SOHO.mydata.baseline.RAE$treatment <- R.flag[SOHO.patientID.RAE]

imputed.SOHO.baseline.RAE.R <- imputed.SOHO.mydata.baseline.RAE[imputed.SOHO.mydata.baseline.RAE$treatment == 1,]

imputed.SOHO.baseline.RAE.AE <- imputed.SOHO.mydata.baseline.RAE[imputed.SOHO.mydata.baseline.RAE$treatment == 0,]

imputed.SOHO.baseline.RAE.R$dosage <- imputed.SOHO.baseline.RAE.R$dosage.R.after

imputed.SOHO.baseline.RAE.AE$dosage <- imputed.SOHO.baseline.RAE.AE$dosage.AE.after

imputed.SOHO.mydata.baseline.RAE <- imputed.SOHO.baseline.RAE <- rbind(imputed.SOHO.baseline.RAE.R, imputed.SOHO.baseline.RAE.AE)

### Usefull function to remove intersection

outersect <- function(x, y) {

sort(c(setdiff(x, y),

setdiff(y, x)))

}

###############################################################

### Preparation of different datasets

### PS matching on the patients in RCT/RCT+X

### under drug R or AE without intersection

###############################################################

patient.ID.R.out.AE.SOHO <- outersect(SOHO.R$patient.ID, intersect(SOHO.R$patient.ID, SOHO.AE$patient.ID))

patient.ID.AE.out.R.SOHO <- outersect(SOHO.AE$patient.ID, intersect(SOHO.R$patient.ID, SOHO.AE$patient.ID))

patient.ID.R.out.AE <- outersect(RCT.R$patient.ID, intersect(RCT.R$patient.ID, RCT.AE$patient.ID))

patient.ID.AE.out.R <- outersect(RCT.AE$patient.ID, intersect(RCT.R$patient.ID, RCT.AE$patient.ID))

DURATION.ID.R.out.AE <- outersect(SUBPOP.X.DURATION.R$patient.ID, intersect(SUBPOP.X.DURATION.R$patient.ID, SUBPOP.X.DURATION.AE$patient.ID)); RCT.DURATION.R.out.AE <- c(patient.ID.R.out.AE, DURATION.ID.R.out.AE)

DURATION.ID.AE.out.R <- outersect(SUBPOP.X.DURATION.AE$patient.ID, intersect(SUBPOP.X.DURATION.R$patient.ID, SUBPOP.X.DURATION.AE$patient.ID)); RCT.DURATION.AE.out.R <- c(patient.ID.AE.out.R, DURATION.ID.AE.out.R)

PRACTICE.ID.R.out.AE <- outersect(SUBPOP.X.PRACTICE.R$patient.ID, intersect(SUBPOP.X.PRACTICE.R$patient.ID, SUBPOP.X.PRACTICE.AE$patient.ID)); RCT.PRACTICE.R.out.AE <- c(patient.ID.R.out.AE, PRACTICE.ID.R.out.AE)

PRACTICE.ID.AE.out.R <- outersect(SUBPOP.X.PRACTICE.AE$patient.ID, intersect(SUBPOP.X.PRACTICE.R$patient.ID, SUBPOP.X.PRACTICE.AE$patient.ID)); RCT.PRACTICE.AE.out.R <- c(patient.ID.AE.out.R, PRACTICE.ID.AE.out.R)

SUICIDE.ID.R.out.AE <- outersect(SUBPOP.X.SUICIDE.R$patient.ID, intersect(SUBPOP.X.SUICIDE.R$patient.ID, SUBPOP.X.SUICIDE.AE$patient.ID)); RCT.SUICIDE.R.out.AE <- c(patient.ID.R.out.AE, SUICIDE.ID.R.out.AE)

SUICIDE.ID.AE.out.R <- outersect(SUBPOP.X.SUICIDE.AE$patient.ID, intersect(SUBPOP.X.SUICIDE.R$patient.ID, SUBPOP.X.SUICIDE.AE$patient.ID)); RCT.SUICIDE.AE.out.R <- c(patient.ID.AE.out.R, SUICIDE.ID.AE.out.R)

ALCOHOL.ID.R.out.AE <- outersect(SUBPOP.X.ALCOHOL.R$patient.ID, intersect(SUBPOP.X.ALCOHOL.R$patient.ID, SUBPOP.X.ALCOHOL.AE$patient.ID)); RCT.ALCOHOL.R.out.AE <- c(patient.ID.R.out.AE, ALCOHOL.ID.R.out.AE)

ALCOHOL.ID.AE.out.R <- outersect(SUBPOP.X.ALCOHOL.AE$patient.ID, intersect(SUBPOP.X.ALCOHOL.R$patient.ID, SUBPOP.X.ALCOHOL.AE$patient.ID)); RCT.ALCOHOL.AE.out.R <- c(patient.ID.AE.out.R, ALCOHOL.ID.AE.out.R)

SUBSTANCE.ID.R.out.AE <- outersect(SUBPOP.X.SUBSTANCE.R$patient.ID, intersect(SUBPOP.X.SUBSTANCE.R$patient.ID, SUBPOP.X.SUBSTANCE.AE$patient.ID)); RCT.SUBSTANCE.R.out.AE <- c(patient.ID.R.out.AE, SUBSTANCE.ID.R.out.AE)

SUBSTANCE.ID.AE.out.R <- outersect(SUBPOP.X.SUBSTANCE.AE$patient.ID, intersect(SUBPOP.X.SUBSTANCE.R$patient.ID, SUBPOP.X.SUBSTANCE.AE$patient.ID)); RCT.SUBSTANCE.AE.out.R <- c(patient.ID.AE.out.R, SUBSTANCE.ID.AE.out.R)

DURATION.SUICIDE.ID.R.out.AE <- outersect(SUBPOP.X.SUICIDE.DURATION.R$patient.ID, intersect(SUBPOP.X.SUICIDE.DURATION.R$patient.ID, SUBPOP.X.SUICIDE.DURATION.AE$patient.ID)); RCT.DURATION.SUICIDE.R.out.AE <- c(patient.ID.R.out.AE, DURATION.SUICIDE.ID.R.out.AE)

DURATION.SUICIDE.ID.AE.out.R <- outersect(SUBPOP.X.SUICIDE.DURATION.AE$patient.ID, intersect(SUBPOP.X.SUICIDE.DURATION.R$patient.ID, SUBPOP.X.SUICIDE.DURATION.AE$patient.ID)); RCT.DURATION.SUICIDE.AE.out.R <- c(patient.ID.AE.out.R, DURATION.SUICIDE.ID.AE.out.R)

DURATION.ALCOHOL.ID.R.out.AE <- outersect(SUBPOP.X.DURATION.ALCOHOL.R$patient.ID, intersect(SUBPOP.X.DURATION.ALCOHOL.R$patient.ID, SUBPOP.X.DURATION.ALCOHOL.AE$patient.ID)); RCT.DURATION.ALCOHOL.R.out.AE <- c(patient.ID.R.out.AE, DURATION.ALCOHOL.ID.R.out.AE)

DURATION.ALCOHOL.ID.AE.out.R <- outersect(SUBPOP.X.DURATION.ALCOHOL.AE$patient.ID, intersect(SUBPOP.X.DURATION.ALCOHOL.R$patient.ID, SUBPOP.X.DURATION.ALCOHOL.AE$patient.ID)); RCT.DURATION.ALCOHOL.AE.out.R <- c(patient.ID.AE.out.R, DURATION.ALCOHOL.ID.AE.out.R)

DURATION.PRACTICE.ID.R.out.AE <- outersect(SUBPOP.X.DURATION.PRACTICE.R$patient.ID, intersect(SUBPOP.X.DURATION.PRACTICE.R$patient.ID, SUBPOP.X.DURATION.PRACTICE.AE$patient.ID)); RCT.DURATION.PRACTICE.R.out.AE <- c(patient.ID.R.out.AE, DURATION.PRACTICE.ID.R.out.AE)

DURATION.PRACTICE.ID.AE.out.R <- outersect(SUBPOP.X.DURATION.PRACTICE.AE$patient.ID, intersect(SUBPOP.X.DURATION.PRACTICE.R$patient.ID, SUBPOP.X.DURATION.PRACTICE.AE$patient.ID)); RCT.DURATION.PRACTICE.AE.out.R <- c(patient.ID.AE.out.R, DURATION.PRACTICE.ID.AE.out.R)

CGIS.change.SOHO.matchedR <- CGIS.change.RCT.matchedR <- CGIS.change.RCT.DURATION.matchedR <- CGIS.change.RCT.SUICIDE.matchedR <- CGIS.change.RCT.PRACTICE.matchedR <- CGIS.change.RCT.ALCOHOL.matchedR <- CGIS.change.RCT.SUBSTANCE.matchedR <- CGIS.change.RCT.DURATION.SUICIDE.matchedR <- CGIS.change.RCT.DURATION.PRACTICE.matchedR <- CGIS.change.RCT.DURATION.ALCOHOL.matchedR <- rep(0, nb.trial)

CGIS.change.SOHO.matchedAE <- CGIS.change.RCT.matchedAE <- CGIS.change.RCT.DURATION.matchedAE <- CGIS.change.RCT.SUICIDE.matchedAE <- CGIS.change.RCT.PRACTICE.matchedAE <- CGIS.change.RCT.ALCOHOL.matchedAE <- CGIS.change.RCT.SUBSTANCE.matchedAE <- CGIS.change.RCT.DURATION.SUICIDE.matchedAE <- CGIS.change.RCT.DURATION.PRACTICE.matchedAE <- CGIS.change.RCT.DURATION.ALCOHOL.matchedAE <- rep(0, nb.trial)

############# PS matching #####################################

nb.trial <- 1000

size.trial <- 250

for(i in 1:nb.trial){

imputed.SOHO.baseline.RAE$treat <- abs(1-imputed.SOHO.baseline.RAE$treatment)

test.size.SOHO <- test.size.RCT <- test.size.RCT.DURATION <- test.size.RCT.PRACTICE <- test.size.RCT.SUICIDE <- test.size.RCT.ALCOHOL <- test.size.RCT.SUBSTANCE <- test.size.RCT.DURATION.SUICIDE <- test.size.RCT.DURATION.ALCOHOL <- test.size.RCT.DURATION.PRACTICE <- size.trial

###################################

######### PS matching for RCT

###################################

#### Preparation of imputed dataset

imputed.RCT.RAE <- imputed.SOHO.baseline.RAE[which(imputed.SOHO.baseline.RAE$patient %in% patient.ID.R.out.AE | imputed.SOHO.baseline.RAE$patient %in% sample(patient.ID.AE.out.R,test.size.RCT)),]

#### PS matching

m.out.RCT.RAE.test <- matchit(treat ~ age + duration + BMI + compliance.physician + practice + gender + hospitalization + qualitylife.anxiety + qualitylife.vas + qualitylife.mobility + qualitylife.pain + qualitylife.selfcare + qualitylife.activity + country + work.base + house.base + social.base + spouse.base + negative.symptom + positive.symptom + cognitive.symptom + nb.admit + CGIS.baseline + dosage, data=imputed.RCT.RAE, method="nearest", distance="logit", ratio=1)

test.data.RCT.RAE <- match.data(m.out.RCT.RAE.test)

ps.sd.RCT.RAE <- sd(test.data.RCT.RAE$distance)

### The optimal caliper = 0.25 times sd of propensity score

m.out.RCT.RAE <- matchit(treat ~ age + duration + BMI + compliance.physician + practice + gender + hospitalization + qualitylife.anxiety + qualitylife.vas + qualitylife.mobility + qualitylife.pain + qualitylife.selfcare + qualitylife.activity + country + work.base + house.base + social.base + spouse.base + negative.symptom + positive.symptom + cognitive.symptom + nb.admit + CGIS.baseline + dosage, data=imputed.RCT.RAE, method="nearest", distance="logit", caliper=0.25*ps.sd.RCT.RAE, ratio=1)

m.out.RCT.RAE <- m.out.RCT.RAE.test

#### Matched sample

matched.RCT.RAE <- imputed.RCT.RAE$patient[which(m.out.RCT.RAE$weights == 1)]

matched.RCT.R.ID <- imputed.RCT.RAE$patient[which(m.out.RCT.RAE$weights == 1 & m.out.RCT.RAE$treat == 0)]

matched.RCT.AE.ID <- imputed.RCT.RAE$patient[which(m.out.RCT.RAE$weights == 1 & m.out.RCT.RAE$treat == 1)]

###################################

######### PS matching for RCT+CHRONICITY

###################################

#### Preparation of imputed dataset

imputed.RCT.DURATION.RAE <- imputed.SOHO.baseline.RAE[which(imputed.SOHO.baseline.RAE$patient %in% RCT.DURATION.R.out.AE | imputed.SOHO.baseline.RAE$patient %in% sample(RCT.DURATION.AE.out.R,test.size.RCT.DURATION)),]

#### PS matching

m.out.RCT.DURATION.RAE.test <- matchit(treat ~ age + duration + BMI + compliance.physician + practice + gender + hospitalization + qualitylife.anxiety + qualitylife.vas + qualitylife.mobility + qualitylife.pain + qualitylife.selfcare + qualitylife.activity + country + work.base + house.base + social.base + spouse.base + negative.symptom + positive.symptom + cognitive.symptom + nb.admit + CGIS.baseline + dosage, data=imputed.RCT.DURATION.RAE, method="nearest", distance="logit", ratio=1)

test.data.DURATION.RCT.RAE <- match.data(m.out.RCT.DURATION.RAE.test)

ps.sd.DURATION.RCT.RAE <- sd(test.data.DURATION.RCT.RAE$distance)

### The optimal caliper = 0.25 times sd of propensity score

m.out.RCT.DURATION.RAE <- matchit(treat ~ age + duration + BMI + compliance.physician + practice + gender + hospitalization + qualitylife.anxiety + qualitylife.vas + qualitylife.mobility + qualitylife.pain + qualitylife.selfcare + qualitylife.activity + country + work.base + house.base + social.base + spouse.base + negative.symptom + positive.symptom + cognitive.symptom + nb.admit + CGIS.baseline + dosage, data=imputed.RCT.DURATION.RAE, method="nearest", distance="logit", caliper=0.25*ps.sd.DURATION.RCT.RAE, ratio=1)

m.out.RCT.DURATION.RAE<- m.out.RCT.DURATION.RAE.test

#### Matched sample

matched.RCT.DURATION.RAE <- imputed.RCT.DURATION.RAE$patient[which(m.out.RCT.DURATION.RAE$weights == 1)]

matched.RCT.DURATION.R.ID <- imputed.RCT.DURATION.RAE$patient[which(m.out.RCT.DURATION.RAE$weights == 1 & m.out.RCT.DURATION.RAE$treat == 0)]

matched.RCT.DURATION.AE.ID <- imputed.RCT.DURATION.RAE$patient[which(m.out.RCT.DURATION.RAE$weights == 1 & m.out.RCT.DURATION.RAE$treat == 1)]

###################################

######### PS matching for RCT+PRACTICE

###################################

#### Preparation of imputed dataset

imputed.RCT.PRACTICE.RAE <- imputed.SOHO.baseline.RAE[which(imputed.SOHO.baseline.RAE$patient %in% RCT.PRACTICE.R.out.AE | imputed.SOHO.baseline.RAE$patient %in% sample(RCT.PRACTICE.AE.out.R,test.size.RCT.PRACTICE)),]

#### PS matching

m.out.RCT.PRACTICE.RAE.test <- matchit(treat ~ age + duration + BMI + compliance.physician + practice + gender + hospitalization + qualitylife.anxiety + qualitylife.vas + qualitylife.mobility + qualitylife.pain + qualitylife.selfcare + qualitylife.activity + country + work.base + house.base + social.base + spouse.base + negative.symptom + positive.symptom + cognitive.symptom + nb.admit + CGIS.baseline + dosage, data=imputed.RCT.PRACTICE.RAE, method="nearest", distance="logit", ratio=1)

test.data.PRACTICE.RCT.RAE <- match.data(m.out.RCT.PRACTICE.RAE.test)

ps.sd.PRACTICE.RCT.RAE <- sd(test.data.PRACTICE.RCT.RAE$distance)

### The optimal caliper = 0.25 times sd of propensity score

m.out.RCT.PRACTICE.RAE <- matchit(treat ~ age + duration + BMI + compliance.physician + practice + gender + hospitalization + qualitylife.anxiety + qualitylife.vas + qualitylife.mobility + qualitylife.pain + qualitylife.selfcare + qualitylife.activity + country + work.base + house.base + social.base + spouse.base + negative.symptom + positive.symptom + cognitive.symptom + nb.admit + CGIS.baseline + dosage, data=imputed.RCT.PRACTICE.RAE, method="nearest", distance="logit", caliper=0.25*ps.sd.PRACTICE.RCT.RAE, ratio=1)

m.out.RCT.PRACTICE.RAE<- m.out.RCT.PRACTICE.RAE.test

#### Matched sample

matched.RCT.PRACTICE.RAE <- imputed.RCT.PRACTICE.RAE$patient[which(m.out.RCT.PRACTICE.RAE$weights == 1)]

matched.RCT.PRACTICE.R.ID <- imputed.RCT.PRACTICE.RAE$patient[which(m.out.RCT.PRACTICE.RAE$weights == 1 & m.out.RCT.PRACTICE.RAE$treat == 0)]

matched.RCT.PRACTICE.AE.ID <- imputed.RCT.PRACTICE.RAE$patient[which(m.out.RCT.PRACTICE.RAE$weights == 1 & m.out.RCT.PRACTICE.RAE$treat == 1)]

###################################

######### PS matching for RCT+SUICIDE

##################################

#### Preparation of imputed dataset

imputed.RCT.SUICIDE.RAE <- imputed.SOHO.baseline.RAE[which(imputed.SOHO.baseline.RAE$patient %in% RCT.SUICIDE.R.out.AE | imputed.SOHO.baseline.RAE$patient %in% sample(RCT.SUICIDE.AE.out.R,test.size.RCT.SUICIDE)),]

#### PS matching

m.out.RCT.SUICIDE.RAE.test <- matchit(treat ~ age + duration + BMI + compliance.physician + practice + gender + hospitalization + qualitylife.anxiety + qualitylife.vas + qualitylife.mobility + qualitylife.pain + qualitylife.selfcare + qualitylife.activity + country + work.base + house.base + social.base + spouse.base + negative.symptom + positive.symptom + cognitive.symptom + nb.admit + CGIS.baseline + dosage, data=imputed.RCT.SUICIDE.RAE, method="nearest", distance="logit", ratio=1)

test.data.SUICIDE.RCT.RAE <- match.data(m.out.RCT.SUICIDE.RAE.test)

ps.sd.SUICIDE.RCT.RAE <- sd(test.data.SUICIDE.RCT.RAE$distance)

### The optimal caliper = 0.25 times sd of propensity score

m.out.RCT.SUICIDE.RAE <- matchit(treat ~ age + duration + BMI + compliance.physician + practice + gender + hospitalization + qualitylife.anxiety + qualitylife.vas + qualitylife.mobility + qualitylife.pain + qualitylife.selfcare + qualitylife.activity + country + work.base + house.base + social.base + spouse.base + negative.symptom + positive.symptom + cognitive.symptom + nb.admit + CGIS.baseline + dosage, data=imputed.RCT.SUICIDE.RAE, method="nearest", distance="logit", caliper=0.25*ps.sd.SUICIDE.RCT.RAE, ratio=1)

m.out.RCT.SUICIDE.RAE <- m.out.RCT.SUICIDE.RAE.test

matched.RCT.SUICIDE.RAE <- imputed.RCT.SUICIDE.RAE$patient[which(m.out.RCT.SUICIDE.RAE$weights == 1)]

matched.RCT.SUICIDE.R.ID <- imputed.RCT.SUICIDE.RAE$patient[which(m.out.RCT.SUICIDE.RAE$weights == 1 & m.out.RCT.SUICIDE.RAE$treat == 0)]

matched.RCT.SUICIDE.AE.ID <- imputed.RCT.SUICIDE.RAE$patient[which(m.out.RCT.SUICIDE.RAE$weights == 1 & m.out.RCT.SUICIDE.RAE$treat == 1)]

###################################

######### PS matching for RCT+ALCOHOL

###################################

#### Preparation of imputed dataset

imputed.RCT.ALCOHOL.RAE <- imputed.SOHO.baseline.RAE[which(imputed.SOHO.baseline.RAE$patient %in% RCT.ALCOHOL.R.out.AE | imputed.SOHO.baseline.RAE$patient %in% sample(RCT.ALCOHOL.AE.out.R,test.size.RCT.ALCOHOL)),]

#### PS matching

m.out.RCT.ALCOHOL.RAE.test <- matchit(treat ~ age + duration + BMI + compliance.physician + practice + gender + hospitalization + qualitylife.anxiety + qualitylife.vas + qualitylife.mobility + qualitylife.pain + qualitylife.selfcare + qualitylife.activity + country + work.base + house.base + social.base + spouse.base + negative.symptom + positive.symptom + cognitive.symptom + nb.admit + CGIS.baseline + dosage, data=imputed.RCT.ALCOHOL.RAE, method="nearest", distance="logit", ratio=1)

test.data.ALCOHOL.RCT.RAE <- match.data(m.out.RCT.ALCOHOL.RAE.test)

ps.sd.ALCOHOL.RCT.RAE <- sd(test.data.ALCOHOL.RCT.RAE$distance)

### The optimal caliper = 0.25 times sd of propensity score

m.out.RCT.ALCOHOL.RAE <- matchit(treat ~ age + duration + BMI + compliance.physician + practice + gender + hospitalization + qualitylife.anxiety + qualitylife.vas + qualitylife.mobility + qualitylife.pain + qualitylife.selfcare + qualitylife.activity + country + work.base + house.base + social.base + spouse.base + negative.symptom + positive.symptom + cognitive.symptom + nb.admit + CGIS.baseline + dosage, data=imputed.RCT.ALCOHOL.RAE, method="nearest", distance="logit", caliper=0.25*ps.sd.ALCOHOL.RCT.RAE, ratio=1)

m.out.RCT.ALCOHOL.RAE <- m.out.RCT.ALCOHOL.RAE.test

matched.RCT.ALCOHOL.RAE <- imputed.RCT.ALCOHOL.RAE$patient[which(m.out.RCT.ALCOHOL.RAE$weights == 1)]

matched.RCT.ALCOHOL.R.ID <- imputed.RCT.ALCOHOL.RAE$patient[which(m.out.RCT.ALCOHOL.RAE$weights == 1 & m.out.RCT.ALCOHOL.RAE$treat == 0)]

matched.RCT.ALCOHOL.AE.ID <- imputed.RCT.ALCOHOL.RAE$patient[which(m.out.RCT.ALCOHOL.RAE$weights == 1 & m.out.RCT.ALCOHOL.RAE$treat == 1)]

###################################

######### PS matching for RCT+SUBSTANCE

###################################

#### Preparation of imputed dataset

imputed.RCT.SUBSTANCE.RAE <- imputed.SOHO.baseline.RAE[which(imputed.SOHO.baseline.RAE$patient %in% RCT.SUBSTANCE.R.out.AE | imputed.SOHO.baseline.RAE$patient %in% sample(RCT.SUBSTANCE.AE.out.R,test.size.RCT.SUBSTANCE)),]

#### PS matching

m.out.RCT.SUBSTANCE.RAE.test <- matchit(treat ~ age + duration + BMI + compliance.physician + practice + gender + hospitalization + qualitylife.anxiety + qualitylife.vas + qualitylife.mobility + qualitylife.pain + qualitylife.selfcare + qualitylife.activity + country + work.base + house.base + social.base + spouse.base + negative.symptom + positive.symptom + cognitive.symptom + nb.admit + CGIS.baseline + dosage, data=imputed.RCT.SUBSTANCE.RAE, method="nearest", distance="logit", ratio=1)

test.data.SUBSTANCE.RCT.RAE <- match.data(m.out.RCT.SUBSTANCE.RAE.test)

ps.sd.SUBSTANCE.RCT.RAE <- sd(test.data.SUBSTANCE.RCT.RAE$distance)

### The optimal caliper = 0.25 times sd of propensity score

m.out.RCT.SUBSTANCE.RAE <- matchit(treat ~ age + duration + BMI + compliance.physician + practice + gender + hospitalization + qualitylife.anxiety + qualitylife.vas + qualitylife.mobility + qualitylife.pain + qualitylife.selfcare + qualitylife.activity + country + work.base + house.base + social.base + spouse.base + negative.symptom + positive.symptom + cognitive.symptom + nb.admit + CGIS.baseline + dosage, data=imputed.RCT.SUBSTANCE.RAE, method="nearest", distance="logit", caliper=0.25*ps.sd.SUBSTANCE.RCT.RAE, ratio=1)

m.out.RCT.SUBSTANCE.RAE <- m.out.RCT.SUBSTANCE.RAE.test

matched.RCT.SUBSTANCE.RAE <- imputed.RCT.SUBSTANCE.RAE$patient[which(m.out.RCT.SUBSTANCE.RAE$weights == 1)]

matched.RCT.SUBSTANCE.R.ID <- imputed.RCT.SUBSTANCE.RAE$patient[which(m.out.RCT.SUBSTANCE.RAE$weights == 1 & m.out.RCT.SUBSTANCE.RAE$treat == 0)]

matched.RCT.SUBSTANCE.AE.ID <- imputed.RCT.SUBSTANCE.RAE$patient[which(m.out.RCT.SUBSTANCE.RAE$weights == 1 & m.out.RCT.SUBSTANCE.RAE$treat == 1)]

#################################################

######### PS matching for RCT+CHRONICITY+SUICIDE

#################################################

#### Preparation of imputed dataset

imputed.RCT.DURATION.SUICIDE.RAE <- imputed.SOHO.baseline.RAE[which(imputed.SOHO.baseline.RAE$patient %in% RCT.DURATION.SUICIDE.R.out.AE | imputed.SOHO.baseline.RAE$patient %in% sample(RCT.DURATION.SUICIDE.AE.out.R,test.size.RCT.DURATION.SUICIDE)),]

#### PS matching

m.out.RCT.DURATION.SUICIDE.RAE.test <- matchit(treat ~ age + duration + BMI + compliance.physician + practice + gender + hospitalization + qualitylife.anxiety + qualitylife.vas + qualitylife.mobility + qualitylife.pain + qualitylife.selfcare + qualitylife.activity + country + work.base + house.base + social.base + spouse.base + negative.symptom + positive.symptom + cognitive.symptom + nb.admit + CGIS.baseline + dosage, data=imputed.RCT.DURATION.SUICIDE.RAE, method="nearest", distance="logit", ratio=1)

test.data.DURATION.SUICIDE.RCT.RAE <- match.data(m.out.RCT.DURATION.SUICIDE.RAE.test)

ps.sd.DURATION.SUICIDE.RCT.RAE <- sd(test.data.DURATION.SUICIDE.RCT.RAE$distance)

### The optimal caliper = 0.25 times sd of propensity score

m.out.RCT.DURATION.SUICIDE.RAE <- matchit(treat ~ age + duration + BMI + compliance.physician + practice + gender + hospitalization + qualitylife.anxiety + qualitylife.vas + qualitylife.mobility + qualitylife.pain + qualitylife.selfcare + qualitylife.activity + country + work.base + house.base + social.base + spouse.base + negative.symptom + positive.symptom + cognitive.symptom + nb.admit + CGIS.baseline + dosage, data=imputed.RCT.DURATION.SUICIDE.RAE, method="nearest", distance="logit", caliper=0.25*ps.sd.DURATION.SUICIDE.RCT.RAE, ratio=1)

m.out.RCT.DURATION.SUICIDE.RAE <- m.out.RCT.DURATION.SUICIDE.RAE.test

#### Matched sample

matched.RCT.DURATION.SUICIDE.RAE <- imputed.RCT.DURATION.SUICIDE.RAE$patient[which(m.out.RCT.DURATION.SUICIDE.RAE$weights == 1)]

matched.RCT.DURATION.SUICIDE.R.ID <- imputed.RCT.DURATION.SUICIDE.RAE$patient[which(m.out.RCT.DURATION.SUICIDE.RAE$weights == 1 & m.out.RCT.DURATION.SUICIDE.RAE$treat == 0)]

matched.RCT.DURATION.SUICIDE.AE.ID <- imputed.RCT.DURATION.SUICIDE.RAE$patient[which(m.out.RCT.DURATION.SUICIDE.RAE$weights == 1 & m.out.RCT.DURATION.SUICIDE.RAE$treat == 1)]

#################################################

######### PS matching for RCT+CHRONICITY+ALCOHOL

#################################################

#### Preparation of imputed dataset

imputed.RCT.DURATION.ALCOHOL.RAE <- imputed.SOHO.baseline.RAE[which(imputed.SOHO.baseline.RAE$patient %in% RCT.DURATION.ALCOHOL.R.out.AE | imputed.SOHO.baseline.RAE$patient %in% sample(RCT.DURATION.ALCOHOL.AE.out.R,test.size.RCT.DURATION.ALCOHOL)),]

#### PS matching

m.out.RCT.DURATION.ALCOHOL.RAE.test <- matchit(treat ~ age + duration + BMI + compliance.physician + practice + gender + hospitalization + qualitylife.anxiety + qualitylife.vas + qualitylife.mobility + qualitylife.pain + qualitylife.selfcare + qualitylife.activity + country + work.base + house.base + social.base + spouse.base + negative.symptom + positive.symptom + cognitive.symptom + nb.admit + CGIS.baseline + dosage, data=imputed.RCT.DURATION.ALCOHOL.RAE, method="nearest", distance="logit", ratio=1)

test.data.DURATION.ALCOHOL.RCT.RAE <- match.data(m.out.RCT.DURATION.ALCOHOL.RAE.test)

ps.sd.DURATION.ALCOHOL.RCT.RAE <- sd(test.data.DURATION.ALCOHOL.RCT.RAE$distance)

### The optimal caliper = 0.25 times sd of propensity score

m.out.RCT.DURATION.ALCOHOL.RAE <- matchit(treat ~ age + duration + BMI + compliance.physician + practice + gender + hospitalization + qualitylife.anxiety + qualitylife.vas + qualitylife.mobility + qualitylife.pain + qualitylife.selfcare + qualitylife.activity + country + work.base + house.base + social.base + spouse.base + negative.symptom + positive.symptom + cognitive.symptom + nb.admit + CGIS.baseline + dosage, data=imputed.RCT.DURATION.ALCOHOL.RAE, method="nearest", distance="logit", caliper=0.25*ps.sd.DURATION.ALCOHOL.RCT.RAE, ratio=1)

m.out.RCT.DURATION.ALCOHOL.RAE <- m.out.RCT.DURATION.ALCOHOL.RAE.test

#### Matched sample

matched.RCT.DURATION.ALCOHOL.RAE <- imputed.RCT.DURATION.ALCOHOL.RAE$patient[which(m.out.RCT.DURATION.ALCOHOL.RAE$weights == 1)]

matched.RCT.DURATION.ALCOHOL.R.ID <- imputed.RCT.DURATION.ALCOHOL.RAE$patient[which(m.out.RCT.DURATION.ALCOHOL.RAE$weights == 1 & m.out.RCT.DURATION.ALCOHOL.RAE$treat == 0)]

matched.RCT.DURATION.ALCOHOL.AE.ID <- imputed.RCT.DURATION.ALCOHOL.RAE$patient[which(m.out.RCT.DURATION.ALCOHOL.RAE$weights == 1 & m.out.RCT.DURATION.ALCOHOL.RAE$treat == 1)]

#################################################

######### PS matching for RCT+CHRONICITY+PRACTICE

#################################################

#### Preparation of imputed dataset

imputed.RCT.DURATION.PRACTICE.RAE <- imputed.SOHO.baseline.RAE[which(imputed.SOHO.baseline.RAE$patient %in% RCT.DURATION.PRACTICE.R.out.AE | imputed.SOHO.baseline.RAE$patient %in% sample(RCT.DURATION.PRACTICE.AE.out.R,test.size.RCT.DURATION.PRACTICE)),]

#### PS matching

m.out.RCT.DURATION.PRACTICE.RAE.test <- matchit(treat ~ age + duration + BMI + compliance.physician + practice + gender + hospitalization + qualitylife.anxiety + qualitylife.vas + qualitylife.mobility + qualitylife.pain + qualitylife.selfcare + qualitylife.activity + country + work.base + house.base + social.base + spouse.base + negative.symptom + positive.symptom + cognitive.symptom + nb.admit + CGIS.baseline + dosage, data=imputed.RCT.DURATION.PRACTICE.RAE, method="nearest", distance="logit", ratio=1)

test.data.DURATION.PRACTICE.RCT.RAE <- match.data(m.out.RCT.DURATION.PRACTICE.RAE.test)

ps.sd.DURATION.PRACTICE.RCT.RAE <- sd(test.data.DURATION.PRACTICE.RCT.RAE$distance)

### The optimal caliper = 0.25 times sd of propensity score

m.out.RCT.DURATION.PRACTICE.RAE <- matchit(treat ~ age + duration + BMI + compliance.physician + practice + gender + hospitalization + qualitylife.anxiety + qualitylife.vas + qualitylife.mobility + qualitylife.pain + qualitylife.selfcare + qualitylife.activity + country + work.base + house.base + social.base + spouse.base + negative.symptom + positive.symptom + cognitive.symptom + nb.admit + CGIS.baseline + dosage, data=imputed.RCT.DURATION.PRACTICE.RAE, method="nearest", distance="logit", caliper=0.25*ps.sd.DURATION.PRACTICE.RCT.RAE, ratio=1)

m.out.RCT.DURATION.PRACTICE.RAE <- m.out.RCT.DURATION.PRACTICE.RAE.test

#### Matched sample

matched.RCT.DURATION.PRACTICE.RAE <- imputed.RCT.DURATION.PRACTICE.RAE$patient[which(m.out.RCT.DURATION.PRACTICE.RAE$weights == 1)]

matched.RCT.DURATION.PRACTICE.R.ID <- imputed.RCT.DURATION.PRACTICE.RAE$patient[which(m.out.RCT.DURATION.PRACTICE.RAE$weights == 1 & m.out.RCT.DURATION.PRACTICE.RAE$treat == 0)]

matched.RCT.DURATION.PRACTICE.AE.ID <- imputed.RCT.DURATION.PRACTICE.RAE$patient[which(m.out.RCT.DURATION.PRACTICE.RAE$weights == 1 & m.out.RCT.DURATION.PRACTICE.RAE$treat == 1)]

###################################

######### PS matching for SOHO

###################################

#### Preparation of imputed dataset

imputed.SOHO.RAE <- imputed.SOHO.baseline.RAE[which(imputed.SOHO.baseline.RAE$patient %in% patient.ID.R.out.AE.SOHO | imputed.SOHO.baseline.RAE$patient %in% sample(patient.ID.AE.out.R.SOHO,test.size.SOHO)),]

#### PS matching

m.out.SOHO.RAE.test <- matchit(treat ~ age + duration + BMI + compliance.physician + practice + gender + hospitalization + qualitylife.anxiety + qualitylife.vas + qualitylife.mobility + qualitylife.pain + qualitylife.selfcare + qualitylife.activity + country + work.base + house.base + social.base + spouse.base + negative.symptom + positive.symptom + cognitive.symptom + nb.admit + CGIS.baseline+dosage, data=imputed.SOHO.RAE, method="nearest", distance="logit", ratio=1)

test.data.SOHO.RAE <- match.data(m.out.SOHO.RAE.test)

ps.sd.SOHO.RAE <- sd(test.data.SOHO.RAE$distance)

m.out.SOHO.RAE <- m.out.SOHO.RAE.test

### The optimal caliper = 0.25 times sd of propensity score

m.out.SOHO.RAE <- matchit(treat ~ age + duration + BMI + compliance.physician + practice + gender + hospitalization + qualitylife.anxiety + qualitylife.vas + qualitylife.mobility + qualitylife.pain + qualitylife.selfcare + qualitylife.activity + country + work.base + house.base + social.base + spouse.base + negative.symptom + positive.symptom + cognitive.symptom + nb.admit + CGIS.baseline + dosage, data=imputed.SOHO.RAE, method="nearest", distance="logit", caliper=0.25*ps.sd.SOHO.RAE, ratio=1)

m.out.RCT.DURATION.PRACTICE.RAE <- m.out.RCT.DURATION.PRACTICE.RAE.test

#### Matched sample

matched.SOHO.RAE <- imputed.SOHO.RAE$patient[which(m.out.SOHO.RAE$weights == 1)]

matched.SOHO.R.ID <- imputed.SOHO.RAE$patient[which(m.out.SOHO.RAE$weights == 1 & m.out.SOHO.RAE$treat == 0)]

matched.SOHO.AE.ID <- imputed.SOHO.RAE$patient[which(m.out.SOHO.RAE$weights == 1 & m.out.SOHO.RAE$treat == 1)]

############# END OF PS matching #####################################

### Computation of comparative efficacy

## RCT

CGIS.change.RCT.matchedR[i] <- mean(SOHO.R$CGIS.change.3month[which(SOHO.R$patient.ID %in% matched.RCT.R.ID)], na.rm=T)

CGIS.change.RCT.matchedAE[i] <- mean(SOHO.AE$CGIS.change.3month[which(SOHO.AE$patient.ID %in% matched.RCT.AE.ID)], na.rm=T)

## RCT + duraiton

CGIS.change.RCT.DURATION.matchedR[i] <- mean(SOHO.R$CGIS.change.3month[which(SOHO.R$patient.ID %in% matched.RCT.DURATION.R.ID)], na.rm=T)

CGIS.change.RCT.DURATION.matchedAE[i] <- mean(SOHO.AE$CGIS.change.3month[which(SOHO.AE$patient.ID %in% matched.RCT.DURATION.AE.ID)], na.rm=T)

## RCT + suicide

CGIS.change.RCT.SUICIDE.matchedR[i] <- mean(SOHO.R$CGIS.change.3month[which(SOHO.R$patient.ID %in% matched.RCT.SUICIDE.R.ID)], na.rm=T)

CGIS.change.RCT.SUICIDE.matchedAE[i] <- mean(SOHO.AE$CGIS.change.3month[which(SOHO.AE$patient.ID %in% matched.RCT.SUICIDE.AE.ID)], na.rm=T)

## RCT + practice

CGIS.change.RCT.PRACTICE.matchedR[i] <- mean(SOHO.R$CGIS.change.3month[which(SOHO.R$patient.ID %in% matched.RCT.PRACTICE.R.ID)], na.rm=T)

CGIS.change.RCT.PRACTICE.matchedAE[i] <- mean(SOHO.AE$CGIS.change.3month[which(SOHO.AE$patient.ID %in% matched.RCT.PRACTICE.AE.ID)], na.rm=T)

## RCT + alcohol

CGIS.change.RCT.ALCOHOL.matchedR[i] <- mean(SOHO.R$CGIS.change.3month[which(SOHO.R$patient.ID %in% matched.RCT.ALCOHOL.R.ID)], na.rm=T)

CGIS.change.RCT.ALCOHOL.matchedAE[i] <- mean(SOHO.AE$CGIS.change.3month[which(SOHO.AE$patient.ID %in% matched.RCT.ALCOHOL.AE.ID)], na.rm=T)

## RCT + substance

CGIS.change.RCT.SUBSTANCE.matchedR[i] <- mean(SOHO.R$CGIS.change.3month[which(SOHO.R$patient.ID %in% matched.RCT.SUBSTANCE.R.ID)], na.rm=T)

CGIS.change.RCT.SUBSTANCE.matchedAE[i] <- mean(SOHO.AE$CGIS.change.3month[which(SOHO.AE$patient.ID %in% matched.RCT.SUBSTANCE.AE.ID)], na.rm=T)

## RCT + duration + suicide

CGIS.change.RCT.DURATION.SUICIDE.matchedR[i] <- mean(SOHO.R$CGIS.change.3month[which(SOHO.R$patient.ID %in% matched.RCT.DURATION.SUICIDE.R.ID)], na.rm=T)

CGIS.change.RCT.DURATION.SUICIDE.matchedAE[i] <- mean(SOHO.AE$CGIS.change.3month[which(SOHO.AE$patient.ID %in% matched.RCT.DURATION.SUICIDE.AE.ID)], na.rm=T)

## RCT + duration + practice

CGIS.change.RCT.DURATION.PRACTICE.matchedR[i] <- mean(SOHO.R$CGIS.change.3month[which(SOHO.R$patient.ID %in% matched.RCT.DURATION.PRACTICE.R.ID)], na.rm=T)

CGIS.change.RCT.DURATION.PRACTICE.matchedAE[i] <- mean(SOHO.AE$CGIS.change.3month[which(SOHO.AE$patient.ID %in% matched.RCT.DURATION.PRACTICE.AE.ID)], na.rm=T)

## RCT + duration + alcohol

CGIS.change.RCT.DURATION.ALCOHOL.matchedR[i] <- mean(SOHO.R$CGIS.change.3month[which(SOHO.R$patient.ID %in% matched.RCT.DURATION.ALCOHOL.R.ID)], na.rm=T)

CGIS.change.RCT.DURATION.ALCOHOL.matchedAE[i] <- mean(SOHO.AE$CGIS.change.3month[which(SOHO.AE$patient.ID %in% matched.RCT.DURATION.ALCOHOL.AE.ID)], na.rm=T)

## SOHO

CGIS.change.SOHO.matchedR[i] <- mean(SOHO.R$CGIS.change.3month[which(SOHO.R$patient.ID %in% matched.SOHO.R.ID)], na.rm=T)

CGIS.change.SOHO.matchedAE[i] <- mean(SOHO.AE$CGIS.change.3month[which(SOHO.AE$patient.ID %in% matched.SOHO.AE.ID)], na.rm=T)

}

################################################

### Comparative efficacy and comparative effectiveness calculation ################################################

CGIS.change.RCT.matched <- CGIS.change.RCT.matchedR - CGIS.change.RCT.matchedAE

CGIS.change.RCT.DURATION.matched <- CGIS.change.RCT.DURATION.matchedR - CGIS.change.RCT.DURATION.matchedAE

CGIS.change.RCT.SUICIDE.matched <- CGIS.change.RCT.SUICIDE.matchedR - CGIS.change.RCT.SUICIDE.matchedAE

CGIS.change.RCT.PRACTICE.matched <- CGIS.change.RCT.PRACTICE.matchedR - CGIS.change.RCT.PRACTICE.matchedAE

CGIS.change.RCT.ALCOHOL.matched <- CGIS.change.RCT.ALCOHOL.matchedR - CGIS.change.RCT.ALCOHOL.matchedAE

CGIS.change.RCT.SUBSTANCE.matched <- CGIS.change.RCT.SUBSTANCE.matchedR - CGIS.change.RCT.SUBSTANCE.matchedAE

CGIS.change.RCT.DURATION.SUICIDE.matched <- CGIS.change.RCT.DURATION.SUICIDE.matchedR - CGIS.change.RCT.DURATION.SUICIDE.matchedAE

CGIS.change.RCT.DURATION.PRACTICE.matched <- CGIS.change.RCT.DURATION.PRACTICE.matchedR - CGIS.change.RCT.DURATION.PRACTICE.matchedAE

CGIS.change.RCT.DURATION.ALCOHOL.matched <- CGIS.change.RCT.DURATION.ALCOHOL.matchedR - CGIS.change.RCT.DURATION.ALCOHOL.matchedAE

CGIS.change.SOHO.matched <- CGIS.change.SOHO.matchedR - CGIS.change.SOHO.matchedAE

## References used in Supplemental Material

1. James Honaker, Gary King, Matthew Blackwell (2011). Amelia II: A Program for Missing Data. Journal of Statistical Software, 45(7), 1–47. URL http://www.jstatsoft.org/v45/i07/
2. <https://cran.r-project.org/web/packages/Amelia/index.html>
3. Rosenbaum PR, Rubin DB. Constructing a control group using multivariate matched sampling methods that incorporate the propensity score. The American Statistician 39[1], 34-38. 1985.
4. Ho DE, Imai K, King G, Stuart EA. MatchIt: Nonparametric Preprocessing for Parametric Causal Inference. Journal of Statistics Software 42[8], 1-28. 2011.
5. Lieberman JA, Stroup TS, McEvoy JP, Swartz MS, Rosenheck RA, Perkins DO, Keefe RS, Davis SM, Davis CE, Lebowitz BD, Severe J and Hsiao JK (2005). Clinical Antipsychotic Trials of Intervention Effectiveness (CATIE) Investigators Effectiveness of antipsychotic drugs in patients with chronic schizophrenia, New Engl J Med, 353(12): 1209-1223.
6. Kwon JS, Jang JH, Kang D-H, Yoo SY, Kim YK, Cho SJ and the APLUS study group (2009). Long-term efficacy and safety of aripiprazole in patients with schizophrenia, schizophreniform disorder, or schizoaffective disorder: 26-week prospective study, Psychiatry and Clinical Neurosciences, 63(1): 73-81.
7. Kahn RS, Fleischhacker WW, Boter H, Davidson M, Vergouwe Y, Keet IP, Gheo-rghe MD, Rybakowski JK, Galderisi S, Libiger J, Hummer M, Dollfus S, Lopez-Ibor JJ, Hranov LG, Gaebel W, Peuskens J, Lindefors N, Riecher-Rossler A, Grobbee DE; EUFEST study group. (2008). Effectiveness of antipsychotic drugs in first-episode schizophrenia and schizophreniform disorder: an open randomised clinical trial, Lancet, 371(9618): 1085-1097.
8. Umbricht D, Alberati D, Martin-Facklam M, Borroni E, Youssef EA, Ostland M, Wallace TL, Kno ach F, Doringer E, Wettstein JG1, Bausch A, Garibaldi G1, Santarelli L. (2014). Effect of bitopertin, a glycine reuptake inhibitor, on negative symptoms of schizophrenia: a randomized, double-blind, proof-of-concept study, JAMA Psychiatry, 71(6):637-646.
9. Cortesi PA, Mencacci C, Luigi F, Pirfo E, Berto P, Sturkenboom MC, Lopes FL, Giustra MG, Mantovani LG, Scalone L. (2013). Compliance, persistence, costs and quality of life in young patients treated with antipsychotic drugs: results from the COMETA study. BMC Psychiatry, 13(1):98.
10. Lipkovich I, Deberdt W, Csernansky JG, Buckley P, Peuskens J, Kollack-Walker S, Zhang Y, Liu-Seifert H, Houston JP. (2007). Predictors of risk for relapse in patients with schizophrenia or schizoaffective disorder during olanzapine drug therapy. J Psychiatr Res, 41(3-4):305-310.
